# High wax ester and triacylglycerol biosynthesis potential in coastal sediments of Antarctic and Subantarctic environments

**DOI:** 10.1371/journal.pone.0288509

**Published:** 2023-07-17

**Authors:** Virginia Galván, Federico Pascutti, Natalia E. Sandoval, Mariana P. Lanfranconi, Mariana Lozada, Ana L. Arabolaza, Walter P. Mac Cormack, Héctor M. Alvarez, Hugo C. Gramajo, Hebe M. Dionisi

**Affiliations:** 1 Instituto de Biología Molecular y Celular de Rosario (IBR-CONICET, FBIOyF–UNR), Rosario, Santa Fe, Argentina; 2 Instituto de Biociencias de la Patagonia (INBIOP-UNPSJB-CONICET), Comodoro Rivadavia, Chubut, Argentina; 3 Instituto de Biología de Organismos Marinos (IBIOMAR-CONICET), Puerto Madryn, Chubut, Argentina; 4 Instituto de Nanobiotecnología (NANOBIOTEC-UBA-CONICET), San Martín, Ciudad Autónoma de Buenos Aires, Argentina; 5 Instituto Antártico Argentino (IAA), San Martín, Buenos Aires, Argentina; 6 Centro para el Estudio de Sistemas Marinos (CESIMAR-CONICET), Puerto Madryn, Chubut, Argentina; Bristol-Myers Squibb Company, UNITED STATES

## Abstract

The wax ester (WE) and triacylglycerol (TAG) biosynthetic potential of marine microorganisms is poorly understood at the microbial community level. The goal of this work was to uncover the prevalence and diversity of bacteria with the potential to synthesize these neutral lipids in coastal sediments of two high latitude environments, and to characterize the gene clusters related to this process. Homolog sequences of the key enzyme, the wax ester synthase/acyl-CoA:diacylglycerol acyltransferase (WS/DGAT) were retrieved from 13 metagenomes, including subtidal and intertidal sediments of a Subantarctic environment (Ushuaia Bay, Argentina), and subtidal sediments of an Antarctic environment (Potter Cove, Antarctica). The abundance of WS/DGAT homolog sequences in the sediment metagenomes was 1.23 ± 0.42 times the abundance of 12 single-copy genes encoding ribosomal proteins, higher than in seawater (0.13 ± 0.31 times in 338 metagenomes). Homolog sequences were highly diverse, and were assigned to the Pseudomonadota, Actinomycetota, Bacteroidota and Acidobacteriota phyla. The genomic context of WS/DGAT homologs included sequences related to WE and TAG biosynthesis pathways, as well as to other related pathways such as fatty-acid metabolism, suggesting carbon recycling might drive the flux to neutral lipid synthesis. These results indicate the presence of abundant and taxonomically diverse bacterial populations with the potential to synthesize lipid storage compounds in marine sediments, relating this metabolic process to bacterial survival.

## Introduction

The ability of certain bacteria to accumulate neutral lipids, such as triacylglycerols (TAG) and wax esters (WE), as storage of carbon and energy has been known for several years. This property is considered to be an adaptation of some organisms to extreme environments in which nutrient supplies might be intermittent, giving the WE/TAG producers the ability to survive long periods of starvation [1]. The extremely hydrophobic properties of TAG and WE allow their accumulation in large amounts in cells without changing the osmolarity of the cytoplasm. In addition, their accumulation is physiologically advantageous because their oxidation produce the maximum yields of energy in comparison with other storage compounds such as carbohydrates or PHA, since the carbon atoms of the acyl moieties of these compounds are in their most reduced form [2]. Likely, TAG and/or WE may play a key role in resource allocation and survival strategies in microbial communities of marine environments. The storage of these compounds may allow microorganisms to decouple their metabolic activity for immediate resource supply, supporting more diverse microbial responses to environmental changes [3]. In this way, storage of TAG and/or WE may have a stabilizing influence on microbial communities exposed to fluctuating conditions, such as those occurring in coastal environments.

The accumulation of WE/TAG has been experimentally confirmed in a rather reduced group of bacteria, mainly in different genera of actinobacteria and in some gammaproteobacteria. However, the potential to synthesize these storage lipids has been considerably extended now to several other groups of prokaryotes, based in the analysis of genomes and the identification of sequences potentially coding for key enzymes involved in the synthesis of WE or TAG [4]. Wax ester synthases (WS, EC 2.3.1.75) are enzymes that catalyze the synthesis of WE by the condensation of a fatty alcohol and a fatty acyl-Coenzyme A (acyl-CoA), whereas diacylglycerol-*O*-acyltransferases (DGAT, EC 2.3.1.20) are the enzymes that catalyze the transesterification of acyl-CoA with diacylglycerol (DAG), to synthesize TAG (For reviews see [5, 6]). Several acyltransferases identified in bacteria are bifunctional (known as WS/DGAT enzymes), because they catalyze the synthesis of WE and/or TAG depending on the availability of metabolic precursors present in the cell [7–10]. Interestingly, the ability to accumulate WE, TAG or both compounds highly depends on the substrates provided through the host’s metabolism, and also on the environmental conditions. Therefore, in many cases, the *in vivo* accumulation of one compound or the other does not necessarily correlate with the enzymatic properties of the WS/DGAT present in the corresponding host [11].

WS/DGAT enzymes seem to be essential for the synthesis and accumulation of TAG and/or WE in prokaryotes, according to the following evidences: (i) WS/DGAT enzymes were detected in all TAG/WE-synthesizing bacteria, whereas they were absent in those bacteria unable to produce such neutral lipids; (ii) the overexpression of WS/DGAT enzymes in their native bacterial hosts promoted an increase of WE and/or TAG accumulation [7, 12–15]; and (iii) the heterologous expression of WS/DGAT enzymes from TAG/WE-synthesizing bacteria, such as those from *Acinetobacter baylyi* ADP1 or from *Streptomyces coelicolor* in *Escherichia coli*, conferred the ability to produce TAG in this bacterial host, which is not naturally able to synthesize these neutral lipids [16, 17]. Similarly, low amounts of TAG were produced when the enzyme AtfA from *A*. *baylyi* ADP1 was expressed in the cyanobacterium *Synechocystis* sp. PCC 6803 [18].

WS and DGAT acyltransferases are also found in plants, protists, fungi and animals. However, phylogenetic and evolutionary analyses of eukaryotic and prokaryotic genes demonstrated that WS/DGAT enzymes, largely present in prokaryotes, evolved separately with functional convergence during evolution [19]. The first prokaryotic WS/DGAT was reported in *A*. *baylyi* ADP1 [7]. A limited number of WS/DGAT enzymes have been functionally characterized, including those from *A*. *baylyi* [20], *Marinobacter hydrocarbonoclasticus* [21], *Marinobacter aquaeolei* [11], *Alkanivorax borkumensis* [20], *Psychrobacter cryohalolentis* [10], *S*. *coelicolor* [12], *Streptomyces avermitilis* [22], *Mycobacterium tuberculosis* [23], *Mycobacterium bovis* [24], *Rhodococcus opacus* [13, 14], *Rhodococcus jostii* [15], and *Thermomonospora curvata* [25].

Wang *et al*. [4] investigated the theoretical distribution patterns of energy reserves across bacterial taxa, including WE and TAG among other reserve materials. They concluded that specific metabolic pathways and enzymes are restricted to certain bacterial groups. The authors reported that WS/DGAT homolog sequences were identified in 673 out of the 8,282 analyzed bacterial species. These sequences were mainly present in members of Actinobacteria and Proteobacteria. Within the Pseudomonadota, most sequences were found in γ-, δ-, and ε-Proteobacteria. Some members of FCB (Fibrobacterota, Chlorobiota, Bacteroidota) and the PVC (Planctomycetes, Verrucomicrobia, Chlamydiae) groups also contained WS/DGAT homolog sequences, whereas none were detected in species belonging to the Bacillota phylum. The number of WS/DGAT homolog sequences identified in bacterial genomes seems to be a species/strain dependent feature, although in general, Gram-negative TAG/WE-producing bacteria, as well as some species of the *Streptomyces* genus, have a low number of *ws/dgat* genes in their genomes (1–3 copies). In contrast, mycolic acid-containing actinobacteria, such as members of *Mycobacterium* and *Rhodococcus* genera, have a higher copy number of *ws/dgat* in their genomes (5–17 isoenzymes).

The study of WE and TAG biosynthesis potential in isolated bacteria has increased our understanding on the taxonomic distribution of putative sequences of the key enzyme of this pathway, WS/DGAT [4]. Culture-independent studies can provide complementary information essential to get more insights on these processes at the microbial community level, without the cultivation biases that affect these approaches. Limited information is available on the enzymes potentially involved in WE and/or TAG biosynthesis in yet-to-be cultured microorganisms. Lanfranconi *et al*. [26] investigated the diversity of sequences encoding WS/DGAT homolog sequences in natural environments from Northeastern Patagonia (Argentina) using a PCR-based approach. This study showed that sequences retrieved from marine sediments were affiliated to marine OM60 clade (currently in the Cellvibrionales order of Gammaproteobacteria [27], while no putative *ws/dgat* genes could be amplified from seawater samples. In contrast, soils samples contained phylotypes only related to Gram positive actinobacteria, such as *Nocardioides*, *Kribbella*, *Actinomadura*, *Streptomyces*, *Rhodococcus*, *Dietzia* and *Thermomonospora* [26]. Microbial communities from coastal sediments of high latitude ecosystems are exposed to multiple sources of stress, including low temperatures and various anthropogenic impacts [28, 29]. We hypothesized that the capability to accumulate WE or TAG could contribute to their survival under these extreme conditions, and therefore, this trait would be common among members of these microbial communities.

The goals of this work were: (i) to uncover the prevalence of WE and/or TAG biosynthesis capability in microbial communities from Antarctic and Subantarctic sediments, (ii) to identify taxa with the potential to synthesize these storage compounds, (iii) to evaluate the diversity of WS/DGAT homologs, and (iv) to analyze the presence of other genes related with lipid metabolism in their genomic context.

## Materials and methods

### Study sites and metagenomic datasets

**Fig 1:**
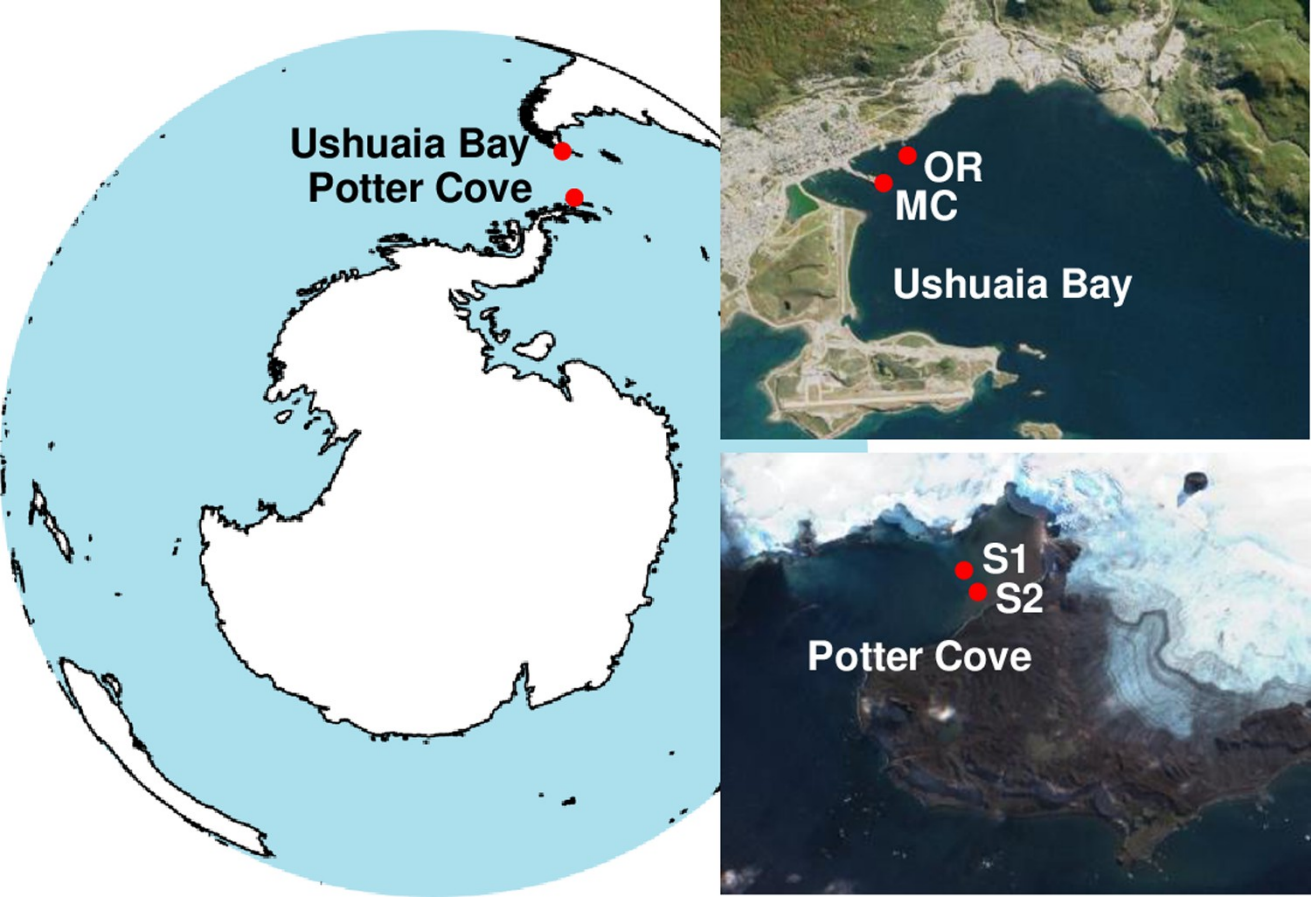
Geographic locations of the study sites. Ushuaia Bay is located on the South coast of Tierra del Fuego Island, Argentina, within the Beagle Channel, and Potter Cove is located on the Southwest coast of the 25 de Mayo (King George) Island, Antarctica. The top 5 cm of the subtidal sediments were sampled in triplicate, in two sites distanced approximately 500 m (bathymetry ranging between 9.5 and 23.45 m). At OR site, Ushuaia Bay, an intertidal sediment sample (0–3 cm) was also obtained, and used for the construction of a fosmid library, from which the dataset was generated by shotgun sequencing. Images: Ushuaia Bay (http://earthobservatory.nasa.gov/); Potter Cove (https://apps.sentinel-hub.com).

A dataset including six metagenomes from coastal sediments of Ushuaia Bay (Tierra del Fuego Island, Argentina) and six from Potter Cove (25 de Mayo/King George Island, South Shetland Islands, Antarctica) was used for this study (S1 Table). In each of these enclosed environments, triplicate samples of superficial (0–5 cm) subtidal sediments were obtained using cores from two sampling sites distanced approximately 500 m (Fig 1). The sediments collected in Ushuaia Bay and Potter Cove contained moderate and low levels of hydrocarbon pollution, respectively [30]. DNA extraction, sequencing (Illumina HiSeq 2000 platform) and functional annotation (Integrated Microbial Genomes & Microbiomes (IMG/M) pipeline, [31]) were similar for the six metagenomes, and were described in previous works [30, 32]. A second metagenomic dataset was obtained by sequencing the fosmids of a metagenomic library (Illumina HiSeq 1500 platform) constructed from intertidal sediments located near the OR sampling sites within Ushuaia Bay, as previously described [33–35].

### Identification of WS/DGAT homolog sequences in metagenomes and genomes

The wax ester synthase-like Acyl-CoA acyltransferase domain (PF03007) was used to search for WS/DGAT homolog sequences in the metagenomes of subtidal sediments of Ushuaia Bay (ARG01—ARG06) and Potter Cove (ANT01—ANT06), using Function Search in the IMG/M system. Both assembled and unassembled metagenomes were used for this analysis (S1 Table). In the case of sequences identified in the assembled dataset, the sequence number was corrected to take into account the scaffold read depth. The corrected values for the assembled metagenome and the number of identified sequences in the unassembled metagenomes are reported as the estimated number of WS/DGAT homolog sequences in the metagenome. The PF03007 domain was also used to search for WS/DGAT homolog sequences in the bacterial genomes of the IMG/M database (March 15, 2022). In the metagenomic dataset generated from a metagenomic library of intertidal sediments, the PF03007 domain was search using the HMMER software (E-value cutoff 10^−5^).

### Relative abundance of WS/DGAT homolog sequences

The relative abundance of sequences containing PF03007 domain in the metagenomes was assessed by calculating the ratio between the estimated number of WS/DGAT homolog sequences in the sediment metagenomes and the estimated number of sequences of each of 12 single copy genes coding for ribosomal proteins (S2 Table). Standard deviation values were calculated for the 12 ratios obtained for each metagenome (coefficient of variation values ranged between 0.11 and 0.18).

### Taxonomic binning of WS/DGAT homolog sequences

The classification of WS/DGAT homolog sequences identified in the six subtidal sediment metagenomes and in the metagenome of intertidal sediments of OR site (Ushuaia Bay), was performed using weighted lowest common ancestor (LCA) algorithm in Megan6 [36] after blastp analysis against the non-redundant NCBI database including the first 100 hits. For the taxonomic assignment of the scaffolds containing the WS/DGAT homolog sequences of the OR07 dataset, the nucleotide sequences were analyzed in the composition-based tool PhylopythiaS [37]. In addition, all the protein coding sequences predicted in the scaffold (MetaGeneMark, [38]) were subjected to taxonomic binning using Megan6, and the result was reported as a consensus assignment of both analyses.

### Clustering and ordination analyses

Sequences containing a PF03007 domain identified in the six subtidal sediment metagenomes were clustered *de novo* into OPUs (operational protein units) using CD-HIT [39], with a sequence identity cutoff value of 0.8. The ordination of metagenomes from subtidal sediments based on OPUs was performed using sequences from the assembled and the unassembled fractions, and the observed frequency was corrected to take into account the estimated gene abundances of sequences in assembled metagenomes. OPUs with low abundance were removed to avoid biases related to the short length of most sequences in this dataset. The analyzed dataset consisted of OPUs containing ≥ 10 sequences (1,027 OPUs). Ordination analyses were performed using nonmetric multidimensional scaling (NMDS), with the metaMDS function in the R package Vegan [40]. Bray-Curtis dissimilarity index, which takes into account the relative abundances of OPUs, was used for ordination and clustering. The OPU table was normalized by Wisconsin double standardization. No transformation of data was applied, to avoid favoring the contribution of very low abundance OPUs in the ordination.

### Statistical analyses

A Wilcoxon Rank Sum test corrected for multiple testing was used to compare relative abundances of OPUs between Antarctic and Subantarctic metagenomes, which was implemented in the R-script ANCOM (https://github.com/FrederickHuangLin/ANCOM). Spearman correlation, Mann-Whitney tests, as well as a Wilcoxon signed rank test to analyze differences in relative abundance of WS/DGAT homolog sequences between free‐living and particle-attached communities in deep sea, were performed in SPSS software (v. 15, SPSS Inc., Chicago, IL, USA).

### Phylogenetic analyses

The phylogenetic analyses included full-length or near full-length metagenomic WS/DGAT homolog sequences and genomic sequences identified in the NCBI and IMG/M databases that shared high identity values with them. First, the protein sequences were aligned using the ClustalW algorithm [41] using default parameters in Jalview [42]. After trimming the ends, the alignments were used to construct phylogenetic trees using the Maximum Likelihood algorithm in MEGAX software [43].

### Genomic context and shared synteny analyses

The genomic contexts of WS/DGAT homolog sequences were compared using the Trebol software (https://inf.imo-chile.cl/software/trebol.html). First, the Rapid Annotations using Subsystems Technology (RAST) software was used for the gene prediction and functional annotation of the scaffolds [44], followed by the manual curation of the results. The annotation of the scaffolds in GenBank format was opened in Trebol, as well as genomic fragments downloaded from GenBank [45]. The genomic and metagenomic fragments at the nucleotide level were compared using discontiguous megablast (dc-megablast) and reported in the figures as different shades of grey connecting regions of the analyzed fragments.

## Results

### WS/DGAT sequence identification

As WS/DGAT enzymes catalyze the key step in bacterial WS and/or TAG biosynthesis, which is exclusive for these pathways, we selected WS/DGAT homolog sequences as marker genes to assess the potential of marine microbial communities to synthesize these neutral lipids. Including both the assembled and the unassembled fractions of the metagenomes, 24,215 homologs were identified in Ushuaia Bay subtidal sediments (Tierra del Fuego, Argentina) and 23,823 homologs in Potter Cove sediments, Antarctica. The estimated gene counts were 28,725 and 31,117 for Ushuaia Bay and Potter Cove respectively, when correcting by assembly read depth (S1 Table). On the other hand, 164 sequences were identified in the metagenome OR07, the dataset constructed from intertidal sediments of Ushuaia Bay. Due to the different strategy used for constructing the two datasets [35], the majority of the sequences identified in the subtidal sediment metagenomes were partial (only 24 sequences were complete), while 74% of the sequences of the OR07 dataset were full length.

To have a frame of reference for the interpretation of the relative abundance of these genes in the metagenomes, we analyzed the number of PF03007-containing sequences in genomes of different bacterial taxa (S3 Table). This dataset includes metagenome-assembled genomes (MAGs) and genomes, the majority of them not complete. Therefore, the reported values might underestimate the number of genes per genomes for these prokaryotic groups. Although some genomes from members of the Actinomycetota and Pseudomonadota phyla had a high number of WS/DGAT paralogs, members of the Actinomycetota phylum had in average a higher number of paralogs compared to Pseudomonadota (S3 Table). Furthermore, almost 60% of the genomes from Actinomycetota contained at least one WS/DGAT homolog, while these genes were detected in only 11% of the genomes from Pseudomonadota. Organisms belonging to several phyla of the FCB group contained one or two WS/DGAT homolog sequences, with 3% of the genomes form Bacteroidota carrying WS/DGAT homologs. In the case of the Acidobacteriota phylum, 8.6% of the genomes contained at least one homolog.

### Relative abundance of WS/DGAT homolog sequences

#### Antarctic and Subantarctic coastal sediments

**Fig 2:**
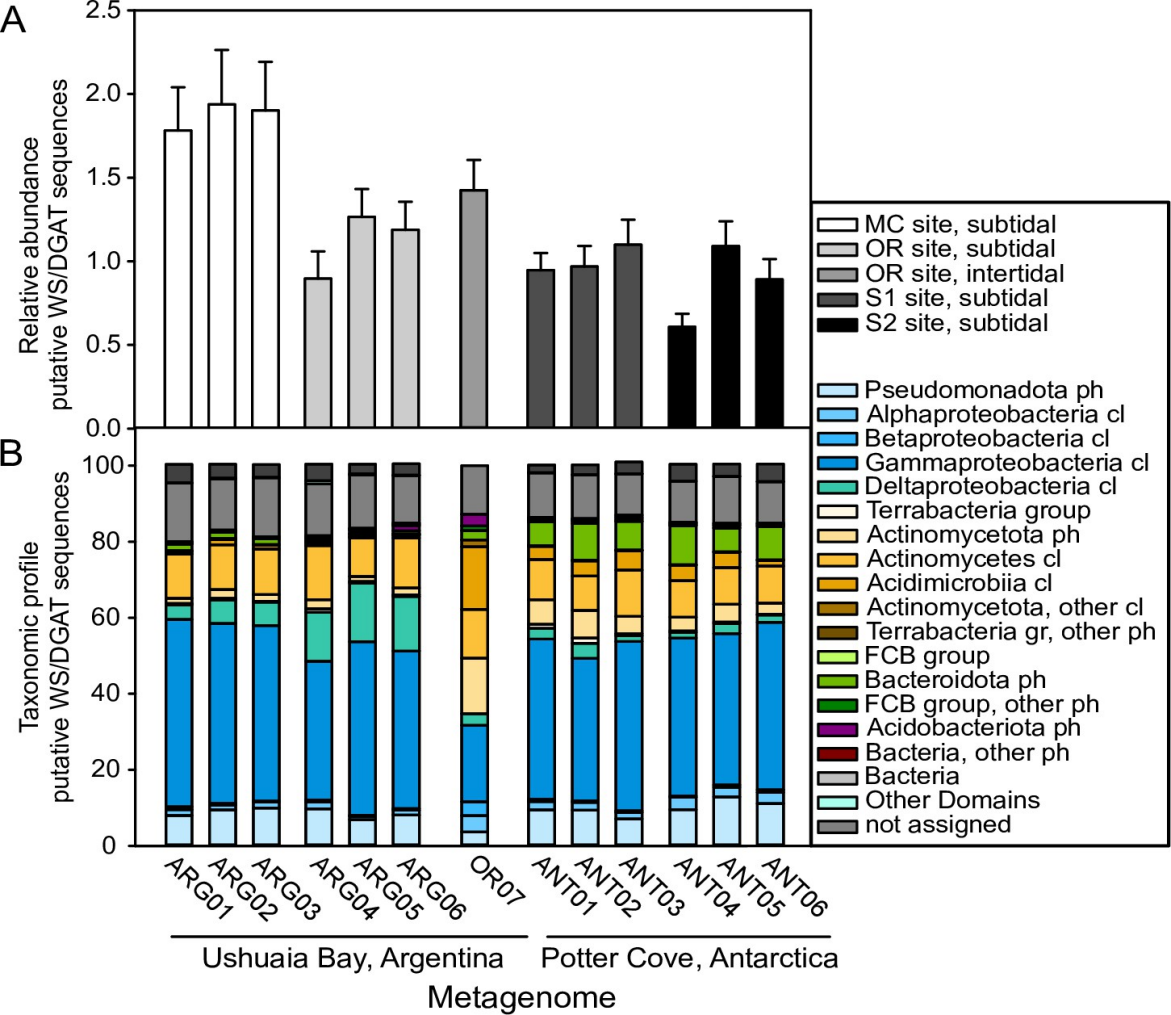
WS/DGAT homolog sequences from metagenomes of Antarctic and Subantarctic sediments. **(A)** Relative abundance of WS/DGAT homolog sequences in sediment metagenomes. The estimated copies of sequences containing the PF03007 domain (unassembled and assembled metagenomes) was normalized by dividing by the estimated copies of 12 selected single-copy genes (S2 Table). Average values ± standard deviation of the 12 calculated ratios are shown. (**B)** Taxonomic profile of WS/DGAT homolog sequences. The sequences were analyzed using blastp (nr, 100 hits), and the Megan6 weighted LCA algorithm was used to classify each sequence. In the case of sequences from the assembled metagenomes of ARG01-ARG06 and ANT01-ANT06, values were corrected based on the scaffold read depth, as indicated in the IMG/M system for sequences of the assembled metagenomes. When group or phylum is indicated, sequences were only assigned to this level; ph, phylum/phyla; cl, class/classes.

We analyzed the abundances of WS/DGAT homolog sequences relative to those of 12 single-copy genes coding for ribosomal proteins to take into consideration differences in the number of protein coding sequences among the metagenomes. The relative abundance of WS/DGAT homolog sequences was remarkably high in the 12 metagenomes of subtidal sediments, with average ratios between the abundance of the WS/DGAT homolog sequences and the single-copy genes ranging between 0.61 and 1.9 (Fig 2A). The relative abundance of sequences in the metagenome of intertidal sediments (OR07) was in agreement with the values obtained in subtidal sediment metagenomes. These values indicate a high potential for WE and/or TAG biosynthesis in these microbial communities, suggesting that this capability can be beneficial for the survival of the microorganisms in the challenging environmental conditions of the sediments of high latitude coastal environments. No statistically significant correlation were found between the relative abundance of WS/DGAT sequences in the 12 subtidal sediment metagenomes and previously reported [30] total hydrocarbon or total polycyclic aromatic hydrocarbon concentrations in the sediments (Spearman correlation, *p* = 0.377 and 0.649, respectively). One of the two sampling sites within Ushuaia Bay had the highest relative abundances among all analyzed metagenomes (MC site, samples ARG01, ARG02 and ARG03), which showed a significant difference with the abundance in OR site (samples ARG04, ARG05 and ARG06), although the analysis showed a weak *p* value (Mann-Whitney test, *p* = 0.05). Both sites suffer poor water quality, although MC site is most affected by untreated domestic wastewater and port activities, and OR site by chronic hydrocarbon pollution [30, 35, 46].

#### Other marine habitats

In order to compare the relative abundance of WS/DGAT homolog sequences in sediment metagenomes with those from other marine habitats and geographic locations, 558 additional marine metagenomes of the Integrated Microbial Genomes and Metagenomes (IMG/M) system were analyzed for their WE and/or TAG biosynthetic potential. These metagenomes were selected based on their classification as “ecosystem type” marine, and likewise containing assembled and unassembled fractions in order to increase the comparability of these datasets. Although there are additional sources of variability in this dataset when compared to the most homogenous processing in those from Subantarctic and Antarctic sediments, differences in relative abundances can be observed among habitats (S1A Fig and S4 Table). Sediment metagenomes presented significantly higher relative abundances of WS/DGAT homolog sequences than seawater samples (p < 0.001, Mann-Whitney test). When analyzing the metagenomes from deep ocean samples (2000–4000 m depth) from the Global Malaspina Expedition [47], a significant difference was observed in the relative abundance of WS/DGAT homolog sequences between free‐living (0.2–0.8 μm) and particle-attached (0.8–20 μm) microbial communities (Z = -4.432, p < 0.001, Wilcoxon signed rank test; S1B Fig). Overall, these results suggest that this trait could be selected in organisms with ecological niches related to an immobilized lifestyle, although it is not possible to know if this result is due to differences in the abundance of organisms carrying these genes and/or the presence of organisms with a higher number of putative *ws/dgat* genes in their genome.

### Taxonomic profile of WS/DGAT homolog sequences

We explored the potential taxonomic origin of the sequences identified in the Antarctic and Subantarctic sediment metagenomes, in order to gain insight into the diversity of taxa with WS and/or TAG biosynthesis potential in these microbial communities. Fig 2B shows the taxonomic profiles of the WS/DGAT homologs from metagenomes of subtidal sediments from Subantarctic and Antarctic environments (ARG01—ARG06 and ANT01—ANT06, respectively). The loss of information that occurs during assembly was considered in the calculation of the relative abundance of sequences assigned to the different taxonomic groups, as in previous analyses. Most of the sequences (79.3–87.2%) were assigned at the phylum level within the Bacteria domain.

The taxonomic binning of the WS/DGAT homolog sequences showed a predominance of sequences from members of the Pseudomonadota phylum in all the subtidal sediment metagenomes (Fig 2B). Gammaproteobacteria was the most abundant class, followed by Alphaproteobacteria and Betaproteobacteria. Within Gammaproteobacteria, sequences were mostly assigned to the orders Cellvibrionales, Pseudomonadales and Moraxellales, representing overall 20.9 ± 3.1 and 23.1 ± 3% of the estimated sequences in Subantarctic and Antarctic sediment metagenomes, respectively. Sequences assigned to the *Halioglobus* genus (Cellvibrionales order, Halieaceae family) were abundant in all subtidal sediment metagenomes, while those assigned to the *Oceanicoccus* genus (Cellvibrionales order, Spongiibacteraceae family) were more prevalent in Antarctic sediment metagenomes (S2 Fig). The number of WS/DGAT homolog sequences was remarkably high in the genomes of *Halioglobus* genus members (S3 Table), with up to 17 copies (*Halioglobus pacificus* RR3-57), suggesting that they could be major contributors to the high relative abundance of WS/DGAT homolog sequences in these metagenomes. Although WS/DGAT homologs were not abundant in genomes of *Oceanicoccus* spp., these sequences were present in 87% of the genomes from the Spongiibacteraceae family, with an average of 5.62 ± 3.35 copies per genome. Within the Pseudomonadales order, on the other hand, homolog sequences assigned to the *Pseudomonas* genus were identified in all sediment metagenomes, while sequences assigned to the *Psychrobacter* genus were more abundant in Antarctic sediment metagenomes, in accordance with its higher latitude (S2 Fig). When analyzing the genomes of members of these genera, WS/DGAT homolog sequences were detected in only 2.52% of the genomes from *Pseudomonas* spp., while 96% of the genomes from *Psychrobacter* contained these genes (S3 Table). The number of homologs per genome was usually low, with the exception of *Pseudomonas pohangensis* DSM 17875 [48], which contains 10 copies. On the other hand, WS/DGAT homolog sequences assigned to Alphaproteobacteria and Betaproteobacteria classes were present in the 12 metagenomes from subtidal sediments, although with lower abundances (Fig 2B). The orders Sphingomonadales and Burkholderiales, respectively, were represented in all the metagenomes of the dataset. WS/DGAT homolog sequences were identified in approximately 10 to 15% of the genomes from the three classes (S3 Table).

Sequences assigned to the Deltaproteobacteria class (Bacteria domain; delta/epsilon subdivisions) were significantly more abundant in metagenomes from Subantarctic sediments than in the ones from Antarctic sediments (p = 0.006, Mann-Whitney test; Fig 2B). The three subtidal sediment metagenomes from the OR sampling site (ARG04—ARG06), located near a fuel storage facility [30], showed a higher prevalence of sequences assigned to the Deltaproteobacteria class than those from the MC sampling site (14.24 ± 1.28% and 5.37 ± 1.39%, respectively). Most of these sequences were not classified at the order level. Up to eight WS/DGAT sequences were identified in MAGs from members of this class (S3 Table), such as Deltaproteobacteria bacterium ARS120 (assembled from metagenomic data from the Tara Ocean Expedition [49]), and Myxococcales bacterium SG8_38 (assembled from a marine sediment metagenome [50]).

Within the Terrabacteria group, WS/DGAT homolog sequences were binned mostly within the Actinomycetota phylum, with fewer sequences assigned to the Chloroflexota, Bacillota and Armatimonadota phyla (Fig 2B). The Actinomycetes class was dominant, and sequences were mostly assigned to the Mycobacteriales (formerly Corynebacteriales) order, and the *Mycobacterium* genus (S2 Fig). In the Acidimicrobiia class, WS/DGAT homologs assigned to the *Ilumatobacter* genus were abundant in Antarctic sediments (S2 Fig). An average of 3.6 WS/DGAT homologs per genome were identified in genomes of *Ilumatobacter* spp., and these sequences were present in all the analyzed genomes (S3 Table). On the other hand, the Microthrixaceae family and ‘*Candidatus* Microthrix’ were detected in nine of the metagenomes (S2 Fig), and sequences assigned to the classes Nitriliruptoria (Euzebyales order), Thermoleophilia (Solirubrobacterales order) and Rubrobacteria (Rubrobacterales order) were present at low abundances.

The FCB group was mainly represented in the dataset by the Bacteroidota phylum (Fig 2B), mostly assigned to the Flavobacteriia and Cytophagia classes. WS/DGAT homolog sequences assigned to these classes were more abundant in Antarctic than in Subantarctic sediment metagenomes (p = 0.004 and p = 0.016, for Flavobacteriia and Cytophagia respectively, Mann-Whitney test). WS/DGAT homolog sequences were identified in only a small proportion of the genomes from these classes (S3 Table). On the other hand, sequences assigned to the Acidobacteriota phylum were detected in all the metagenomes, with relative abundances of up to 1.3% of the identified sequences. More than 8% of the genomes from this phylum contain WS/DGAT sequences, with up to three copies, in the case of *Yimella* sp. cx-573 (S3 Table).

Similar taxa were detected in the intertidal sediment dataset (OR07), although with important differences in their relative abundances (Figs 2B and S2). For instance, the sequences assigned to the Actinomycetota phylum were more prevalent in the intertidal sediment dataset than in subtidal sediment metagenomes, in particular the Acidimicrobiia class (and the *Ilumatobacter* genus). Sequences assigned to the Pseudomonadota phylum, on the other hand, had a lower abundance with most sequences assigned only at the phylum or class level.

### Clustering and ordination analyses

**Fig 3:**
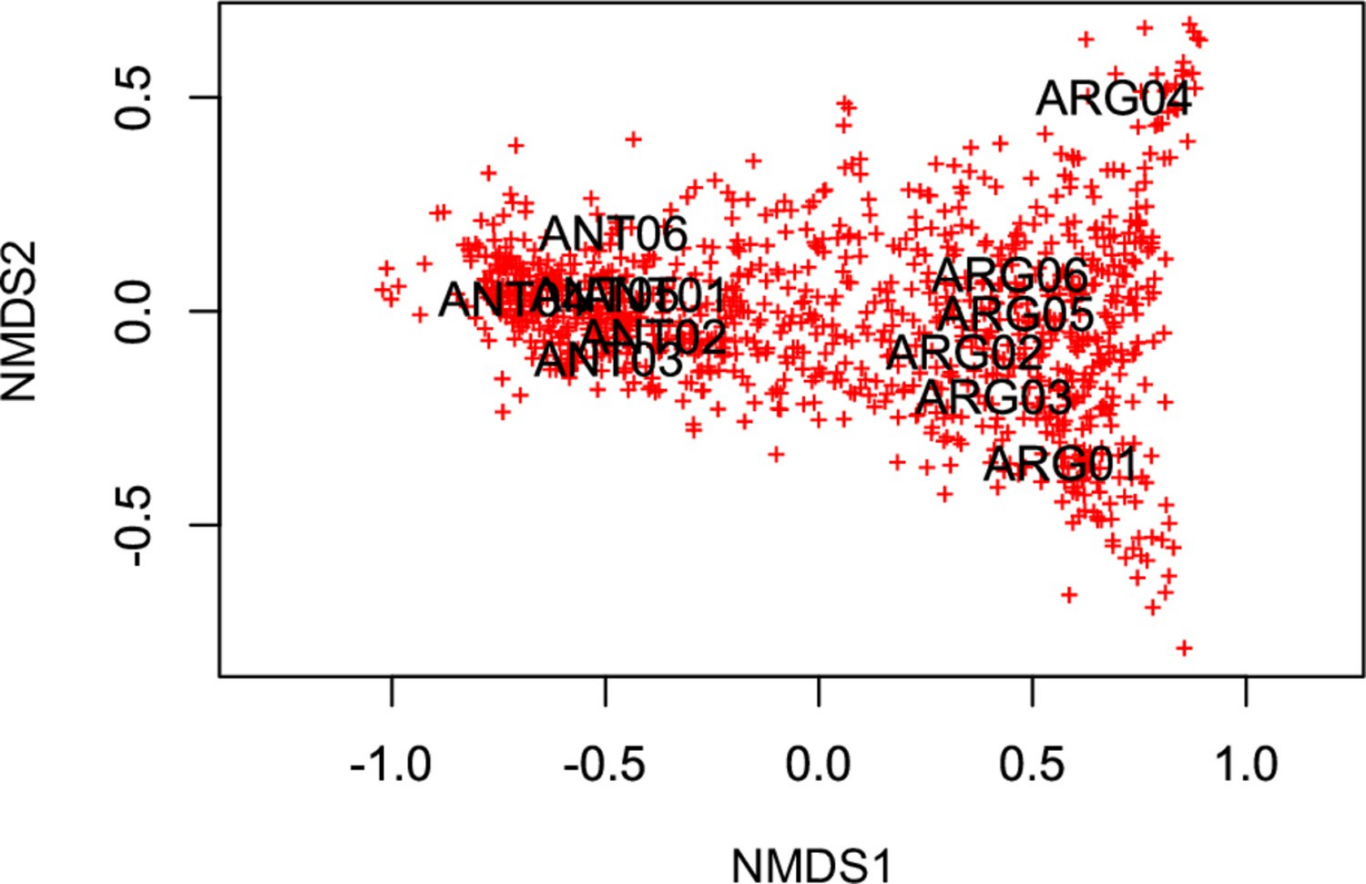
Nonmetric multidimensional scaling of metagenomes based on OPUs of WS/DGAT homolog sequences. The OPU table (OPUs with ≥ 10 sequences) was normalized by Wisconsin double standardization. No transformation of data was applied, in order not to favor the contribution of very low abundance OPUs to ordination. Stress 0.021.

The taxonomic profiles of WS/DGAT homolog sequences identified in the 12 subtidal sediment metagenomes were remarkably similar, with differences in the relative abundance of some taxa (Fig 2B). In order to evaluate the sequence diversity within and between the studied environments, the identified WS/DGAT homolog sequences (S1 Table) were clustered *de novo* into operational protein units (OPUs, sequence identity cutoff value of 0.8). The sequences were clustered into 12,129 OPUs (operational protein units). Potter Cove and Ushuaia Bay metagenomes (ANT01-ANT06 and ARG01-ARG06, respectively) clustered separately with high significance in an NMDS ordination plot based on the 1,022 OPUs containing ≥ 10 sequences (Fig 3, Stress = 0.021; ANOSIM R = 0.9944, p = 0.002). Most of the OPUs, shown in red, were associated with the metagenome grouping pattern, but a series of OPUs can be observed along the axis NMDS1, which contained sequences from both Subantarctic and Antarctic sediments (Fig 3).

A heatmap of the most abundant OPUs was plotted to further assess the contribution of sequences from each of the subtidal sediment metagenomes (S3 Fig). Significant differences (Wilcoxon Rank Sum test) were found between Antarctic and Subantarctic metagenomes in 16 of the 25 OPUs included in the heatmap, which resulted in a clear separation of two clusters. These OPUs contained sequences assigned to six different taxonomic groups, showing the high taxonomic diversity of the most abundant organisms with the capability to synthesize WE and/or TAG. Gammaproteobacteria showed the highest sequence diversity with 13 OPUs, followed by Deltaproteobacteria with five OPUs. S5 Table shows the complete list of OPUs where the abundances were significantly different between both environments. For 74% of the OPUs, the closest sequence from the NCBI nr database were from MAGs, the majority obtained from metagenomes from various marine habitats, including sediments. On the other hand, other OPUs were detected in high abundances in the metagenomes from both Antarctic and Subantarctic sediments. This is the case of OPU4 and OPU22, for which the closest sequences of the NCBI nr database were also uncultured microorganisms from marine sediments (GenBank accession numbers MBT8070888 and AIU93336, respectively).

### WS/DGAT homolog sequences from intertidal and subtidal sediments

As shown in Fig 2, WS/DGAT homolog sequences identified in the intertidal sediment metagenome (OR07) were present at similar relative abundances and mostly included the same taxonomic groups when compared with the sequences identified in the subtidal sediment dataset. The proportion of the different taxa, however, differed relative to subtidal sediment metagenomes, although experimental biases due to previous cloning into a fosmid vector in the OR07 dataset cannot be excluded. S4 Fig shows the relative abundance of sequences identified in subtidal sediment metagenomes clustered in OPUs (CD-HIT, 80% cut off identity, ≥ 10 estimated sequences) that contained sequences from the metagenome of intertidal sediments. Twenty of the OPUs contained sequences assigned to the Actinomycetota phylum, while 13 OPUs to the Pseudomonadota phylum, mostly from the Gammaproteobacteria class. Only one OPU was related to Acidobacteriota and one to Bacteroidota phyla (S4 Fig). Interestingly, the majority of the OPUs included sequences from both, Ushuaia Bay and Potter Cove sediment metagenomes. For instance, OPU2 included sequences from all 13 analyzed metagenomes. From this cluster, sequence 131150_S1-7520 shared 83.23% identity (100% coverage) with the WS/DGAT homolog from *Halioglobus japonicus* (CAA0122667), a mesophilic Gammaproteobacterium from the Cellvibrionales order that was isolated from seawater near Japan [51]. Similarly, OPU17 included sequences from both environments, and was abundant in Potter Cove sediments (S4 Fig). Sequence 344934_S1-313442 (OPU17) shares 76.67% identity (88% coverage) with a WS/DGAT homolog from *Ilumatobacter* sp. Bin SAT196 (MBC49189), a MAG from a TARA Ocean metagenome from the North Atlantic Ocean (Acidimicrobiia class).

### Phylogenetic and genomic context analyses

Contrary to the WS/DGAT homolog sequences identified in subtidal sediment metagenomes where the majority were highly fragmented, 74% of the sequences identified in the sequenced metagenomic library (OR07) were full-length and often contained in long scaffolds (S6 Table). This dataset was used to study the phylogenetic relationships and the genomic context of WS/DGAT homolog sequences assigned to different taxonomic groups, in order to gain further insight into their WE and/or TAG biosynthesis potential.

#### Actinomycetota

The WS/DGAT homolog sequences related to the Terrabacteria group were abundant in the OR07 metagenomic dataset, and almost 40% of the sequences of the dataset binned within the Actinomycetota phylum, or could be assigned at the class level within Actinomycetes, Acidimicrobiia or Nitriliruptoria (S6 Table). A phylogenetic analysis of these homologs and related sequences from IMG/M and NCBI databases (after clustering analysis using a 65% amino acid sequence identity cutoff) shows four clusters (S5 Fig, Clusters I to IV), with the majority of the metagenomic sequences included within Clusters I, II and III. The Mycobacteriales (such as Mycobacteriaceae and Nocardiaceae families), which usually have a high number of WS/DGAT paralogs, had at least one representative in each cluster indicating a non-monophyletic origin. Several other homolog sequences identified in the OR07 dataset were found scattered in clusters I-III, where characterized WS/DGAT enzymes from *S*. *coelicolor*, *R*. *opacus* PD630, *M*. *tuberculosis* and *T*. *curvata*, were also included. Interestingly, cluster IV includes WS/DGAT sequences reported to be associated with TAG accumulation in oleaginous Mycobacteriales. One sequence derived from the OR07 metagenomic dataset, 144528_S1-8331, clustered within this group.

**Fig 4:**
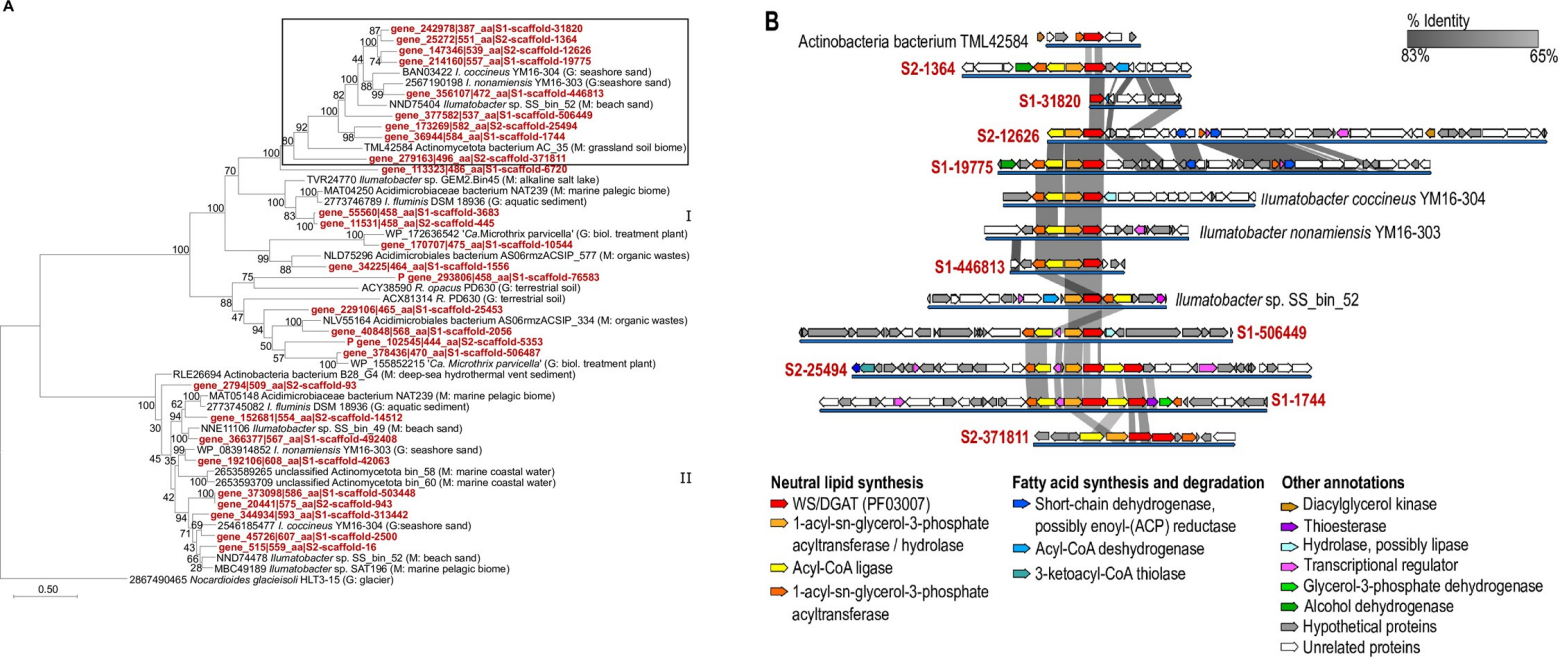
Phylogenetic analysis and genomic context of WS/DGAT homolog sequences assigned to the Actinomycetota phylum. **(A)** Maximum-likelihood tree of WS/DGAT homolog sequences assigned to the Actinomycetota phylum identified in the metagenomic dataset of intertidal sediments (OR07, in red), and related sequences from public databases (in black). Bootstrap values are based on 100 replicates. In parentheses, G sequence identified in a genome, M sequence identified in a metagenome-assembled genome, and environmental origin. The box indicates the sequences for which the genomic context and shared synteny is shown below. **(B)** Genomic context and shared synteny analysis for the cluster indicated in (A). Gray gradient represents % identity at the nucleotide level between scaffolds. References for potential gene function and shared percent identity are indicated in the figure. Pfam domains identified in the deduced amino acid sequences of genes located in the analyzed genomic contexts are shown in S7 Table.

Since many of the WS/DGAT homolog sequences of the OR07 dataset were included in clusters II and III, a new refined phylogenetic tree was generated to gain insights about these novel sequences (Fig 4A). These clusters were specifically chosen due to the existence of robust bootstrap values, as well as, the presence of sequences from organisms of interest, such as those corresponding to *Ilumatobacter* and ‘*Ca*. Microthrix’ genera. Genomic fragments from these organisms, in particular, showed the highest coverage and percent identity at the nucleotide level with the scaffolds assigned to Actinomycetota phylum, in particular in the cluster of genes related to TAG/WE biosynthesis (Fig 4B). Therefore, it is possible that closely related populations were represented within the Actinomycetota members of the microbial communities under study. The phylogenetic tree separated the WS/DGAT homolog sequences into two defined clusters (Fig 4A). Cluster I contains representatives of the Acidimicrobiales order, like ‘*Ca*. Microthrixaceae’ family (‘*Ca*. Microthrix’) and the Acidimicrobiaceae family (genus *Ilumatobacter*), as well as the Mycobacteriales order, Nocardiaceae family (genus *Rhodococcus*). Aft1 and Atf3 from *R*. *opacus* PD630 are the only sequences from terrestrial environments in the cluster, since the rest derived from aquatic environments. Cluster B contained closely related sequences from unclassified members of the Actinomycetota phylum from marine environments, and from members of the *Ilumatobacter* genus.

In the first cluster, two sequences from ‘*Ca*. Microthrix parvicella’ are closely related to metagenomic sequences assigned to the Acidimicrobiia class (Fig 4A). This filamentous actinobacteria, commonly found in activated sludge wastewater treatment plants, is well known for its ability to uptake large amounts of long-chain fatty acids (LCFA), which accumulate as neutral lipids under anaerobic conditions; on the base of this capability, it has been characterized as a specialized lipid consumer [52]. Moreover, metabolic models developed for ‘*Ca*. M. parvicella RN1’ strongly suggests that LCFA are stored as TAGs [53]. This distinctive feature is highlighted by its gene content: it has 10 WS/DGAT homolog sequences (S3 Table). Also within the Acidimicrobiia class, both clusters contain sequences from members of the *Ilumatobacter* genus, like *Ilumatobacter coccineus*, *Ilumatobacter* *nonamiensis*, and *Ilumatobacter fluminis*. These microorganisms have been isolated from *seashore* sand and from the sediment of an estuary [54, 55]. Their genomes have 5, 4 and 3 copies of WS/DGAT homolog genes, respectively (Fig 4). In addition, sequences from other members of this genus, without classification at the species level, were also included in this analysis. Interestingly, the presence of organisms affiliated with the *Ilumatobacter* genus was previously inferred by culture independent methods in Arctic and Antarctic marine sediments, as well as within bottom-dwelling crustacean microbiota and the water in which they live [56] [57], as well as in the subtidal sediments analyzed in this study.

The genomic contexts of the WS/DGAT homolog sequences from a selected cluster of the phylogenetic tree highlighted with a black frame were analyzed to identify other genes related to neutral lipid biosynthesis pathways (Fig 4B). A conserved arrangement displays the close proximity to the WS/DGAT (PF03007) of a 1-acyl-sn-glycerol-3-phosphate acyltransferase-AGPAT (PF01553) containing an additional domain associated to hydrolase activity (HAD, haloacid dehalogenase-like hydrolase, PF12710), an acyl-CoA synthase (PF00501, PF13193), and a typical 1-acyl-sn-glycerol-3-phosphate acyltransferase (PF01553). A similar gene arrangement was reported in *R*. *jostii* RHA1 for *atf9*, *plsB* and a *plsC-like* genes (encoding for a predicted fusion of AGPAT and HAD domains); the latter suggested to encode for the acyltransferases of the Kennedy pathway [15]. These genes are transcribed as an operon and *atf9* was the most abundant WS/DGAT transcript under conditions of N-excess [15]. As mentioned, most of these gene arrangements included a putative acyl-CoA synthase. This activity has a fundamental role as control point in activation of fatty acids for their further utilization. In addition to the presence of this highly conserved cluster of genes, many sequences involved in fatty acid biosynthesis and β-oxidation cycles, such as an acyl-CoA dehydrogenase, an enoyl-ACP reductase, and a 3-ketoacyl-CoA thiolase, were also identified in the proximity of the WS/DGAT homologs (Fig 4B). Related to these findings, it has been described a greater abundance of genes implicated in biosynthesis and degradation of lipids in oleaginous rhodococcal genomes, in comparison with non-oleaginous strains [58]. Other putative genes involved in enzymatic steps related to lipid metabolism were detected as well: diacylglycerol kinase, thioesterase, putative lipases, alcohol dehydrogenase and glycerol 3-phosphate dehydrogenase. The implication of glycerol 3-phosphate dehydrogenase activity in the homeostasis of glycerol-3-phosphate, key substrate for TAG biosynthesis, has been suggested [59].

#### Pseudomonadota

Together with Actinomycetota, one of the most abundant taxonomic assignments at the phylum level in the WS/DGAT sequence dataset was Pseudomonadota. At the class level, WS/DGAT sequences identified in the intertidal sediment metagenome were assigned to Gammaproteobacteria, Alphaproteobacteria, and Betaproteobacteria. Representatives of the Gammaproteobacteria have been frequently reported and studied for their ability to synthesize WE and/or TAG [2]. However, an in-depth analysis in other classes of this phylum has never been reported and this work acknowledges for the first time their WE and TAG biosynthetic potential.

**Fig 5:**
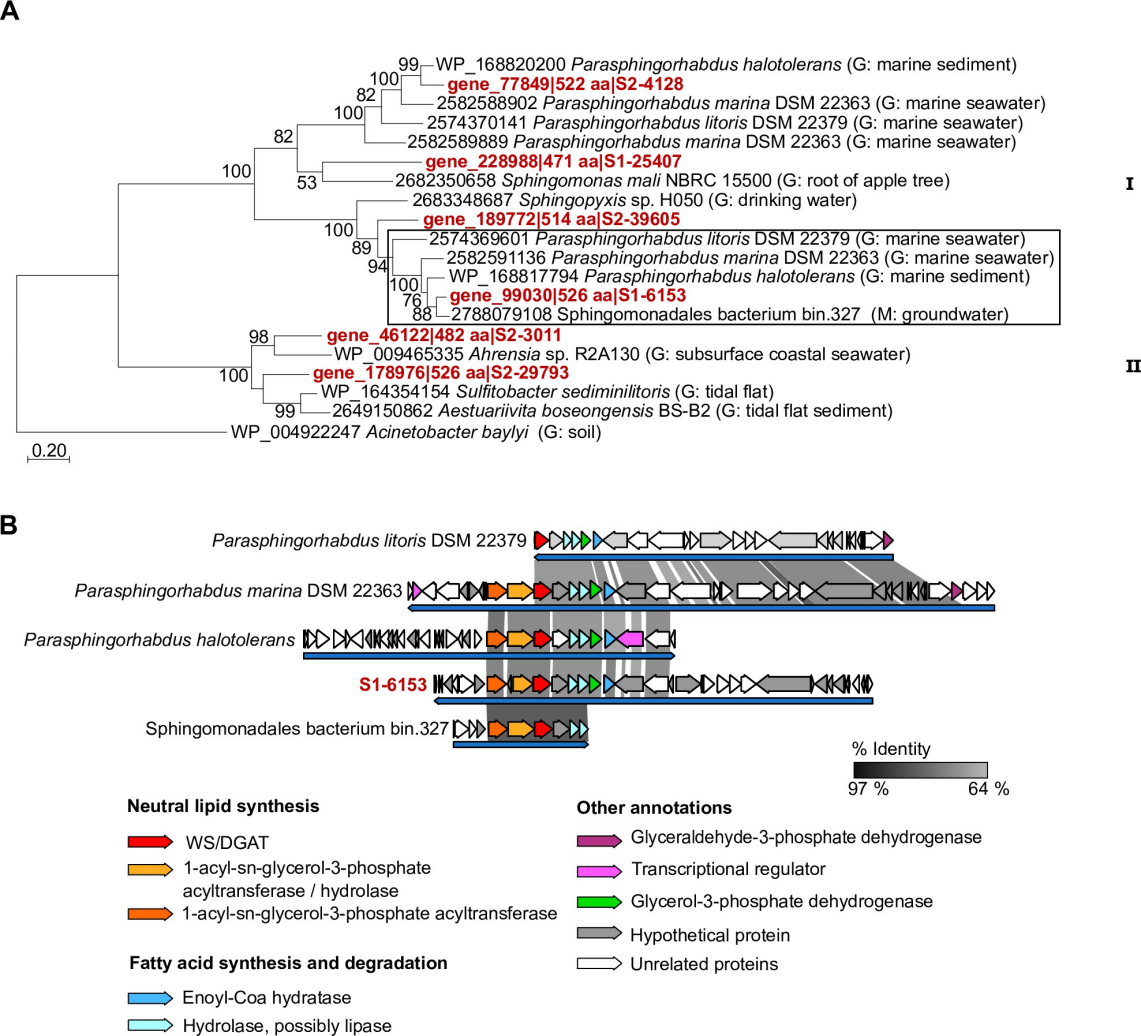
Phylogenetic analysis and genomic context of WS/DGAT homolog sequences assigned to the Alphaproteobacteria class. **(A)** Maximum-likelihood tree of WS/DGAT homolog sequences assigned to the Alphaproteobacteria class, identified in the metagenomic dataset of intertidal sediments (OR07, in red), and related sequences from public databases (in black). Bootstrap values (> 50%) are based on 100 replicates. For each sequence, the environmental origin is indicated: G, sequence identified in a genome; M, sequence identified in a metagenome assembled genome. The box indicates the sequences for which the shared synteny is shown below. **(B)** Genomic context and shared synteny analysis of the cluster indicated in (A). Gray gradient represents % identity at the nucleotide level between scaffolds. References for potential gene function and shared percent identity are indicated in the figure. Pfam domains identified in the deduced amino acid sequences of genes located in the analyzed genomic contexts are shown in S7 Table.

A phylogenetic analysis of WS/DGAT homolog sequences assigned to Alphaproteobacteria and close relatives from genomes showed two clusters, cluster I containing representatives of the Sphingomonadaceae family, whereas cluster II includes members of the Hyphomicrobiales y Rhodobacterales orders (Fig 5A). Among the former, *Parasphingorhabdus halotolerans* JK6, an isolate obtained from marine sediments [60], shared high identity values (70.2–81.2%) with three sequences of the metagenomic dataset. A complete set of genes encoding homolog sequences of Kennedy pathway enzymes, and genes involved in β-oxidation located adjacent to the *ws/dgat* homolog were found in scaffold S1-6153 of the OR07 metagenomic dataset (Fig 5B). A similar gene content and organization was found in *P*. *halotolerans* JK6. In scaffold S2-4128, the putative *ws/dgat* gene was identified followed by sequences related to enzymes involved in the degradation and/or synthesis of fatty acids (GenBank accession number OP731417), in a similar organization than in *P*. *halotolerans* JK6. Cluster II of the phylogenetic tree (Fig 5A) is represented by *Ahrensia* sp. R2A130 and *Sulfitobacter sediminilitoris* JBTF-M27 [61], which have a WS/DGAT homolog and a similar gene organization to S2-3011 and S2-29793, respectively (accession numbers OP731418 and OP731419). In both scaffolds, as well as in the genomes of the above-mentioned strains, this region contains genes mainly involved in PHA metabolism and its regulation.

A phylogenetic analysis of the six WS/DGAT homolog sequences assigned to the Betaproteobacteria class (S6 Table) showed their close relationship with sequences from members of the order Burkholderiales and their distribution in three different clusters (S6A Fig). The genomic context of the sequences in the first cluster (S6B Fig) showed a gene encoding a putative acyl-CoA binding protein (ACBP) adjacent to the *ws/dgat* homolog gene, which could increase efficiency of TAG accumulation [62]. In addition to ACBP, a putative short-chain dehydrogenase/reductase (SDR) oxidoreductase gene was found in scaffolds S2-55933 and S2-3704 (accession numbers OP731421 and OP731420), exhibiting identities of 59.2% and 58.8% at the amino acid level with MAQU_2507 (fatty acyl-CoA reductase), respectively. This enzyme is a fatty acyl-CoA reductase that provides fatty alcohol intermediates for WE synthesis in *M*. *aquaeolei VT8* [63]. Furthermore, these two scaffolds contain a sequence associated with the biosynthesis of PHA (polyhydroxyalkanoic acid system family protein). On the other hand, the scaffold S1-6207 contains a gene encoding a putative phasin involved in the PHA synthesis metabolism (S6B Fig). The scaffold S1-371 (accession number OP731423), which is close to a metagenome assembled genome of Comamonadaceae bacterium EBPR, contains sequences associated to the degradation of fatty acid, such as acyl-CoA dehydrogenase, enoyl-CoA hydratase and 3-hydroxyacyl-CoA dehydrogenase.

S7A Fig shows a phylogenetic tree of WS/DGAT homolog sequences assigned to Gammaproteobacteria, which formed four clusters. Clusters I and IV mostly include sequences from Cellvibrionales, while cluster II is more diverse. Cluster III showed sequences affiliated with Chromatiales and Xanthomonadales. Within these scaffolds, genes potentially coding for different enzymes related to fatty acid metabolism were observed: (i) β-oxidation such as enoyl-CoA hydratase, acetyl-CoA dehydrogenase, acetyl-CoA C-acyltransferase, (ii) enzymes associated to biosynthesis and/or degradation of fatty-acids, i.e. long- chain-fatty-acid-CoA- ligase, or (iii) associated to pentose phosphate pathway which frequently generates NADPH for lipid biosynthesis (S1-7812, S1-427102 and S1-11543, accession numbers OP731428, OP731429 and OP731430, respectively). The gene organization in the scaffolds from the intertidal sediment metagenome was compared to those from close relatives. Interestingly, the scaffolds S2-87379 and S1-7808 (OP731424 and OP731425, respectively) contain a complete set of genes for the Kennedy pathway arranged next to the *ws/dgat* homolog gene (S7B Fig). To our knowledge, this functional gene organization related to the biosynthesis of neutral lipids has not been reported in members of Gammaproteobacteria. In addition, the scaffold S1-87379 contained, next to a gene coding for a putative GPAT, a putative NAD(P)H-dependent glycerol-3-phosphate dehydrogenase that could provide glycerol residues for lipogenesis (S7B Fig). This scaffold shared several genes, including those of the Kennedy pathway, with *Arenicella xantha* DSM 24032. The scaffold S1-7808 had a similar gene organization near the *ws/dgat* homolog with a genome fragment of Unclassified Woeseiaceae bin JSS_woes1, an uncultured organism from marine sediments. Both, scaffold S1-7808 and *Woeseia* sp. StnF contained genes coding for a WS/DGAT and an alpha/beta hydrolase. The scaffold also shares genes coding for a GPAT, a putative lipase and hypothetical proteins with Unclassified Woeseiaceae bin JSS_woes1, and a PAP/AGPAT with *Woeseia* sp. StnF (S7B Fig). On the other hand, scaffold S1-7634 (accession number OP731427) contained genes coding for GPAT and AGPAT near the putative WS/DGAT, missing only a PAP to complete the Kennedy pathway. In addition, upstream to the putative *ws/dgat* gene, this scaffold contains a SDR oxidoreductase that shares the same pfam and shows 49.5% identity at the amino acid level to the enzyme MAQU_2507 (fatty acyl-CoA reductase) involved in WE synthesis in *M*. *aquaeolei* VT8 [63]. Similarly, a gene coding for a putative SDR oxidoreductase sharing 61.9% identity at the amino acid level with sequence MAQU_2507 is located adjacent to the *ws/dgat* homolog of scaffold S1-6186 (**S7C Fig**). As in some scaffolds assigned to Betaproteobacteria, a gene encoding a putative acyl-CoA binding protein (ACBP), with a potential role in neutral lipid biosynthesis is located near the *ws/dgat* homolog gene.

#### Deltaproteobacteria

Similar to Alphaproteobacteria, the relative abundance of WS/DGAT homolog sequences assigned to the Deltaproteobacteria class was low, and most sequences were partial. Interestingly, the sequences most closely related were from uncultured Deltaproteobacteria from marine environments (S8 Fig). The scaffolds containing the putative *ws/dgat* genes were too short or distantly related to annotated genomes to analyze their shared synteny. The synthesis and accumulation of WE and/or TAG have not been reported in members of the Deltaproteobacteria class.

#### Bacteroidota

The four sequences of the OR07 dataset assigned to the Bacteroidota phylum (sequence length 613 ± 15 amino acids) included a C-terminal domain, where a PF02036 domain (SCP-2 sterol transfer family) was detected in two of the identified sequences. WS/DGAT homolog sequences identified in the genomes from members of the *Polaribacter*, *Muricauda*, *Ulvibacter*, *Dokdonia*, *Winogradskyella*, *Lacinutrix* and in some species of the *Tenacibaculum* genera, as well as from several uncultured Bacteroidota, also had a similar length. This domain has been shown to present different roles, such as lipid binding and transfer and catalytic assistance, among others [64]. A maximum-likelihood tree was constructed with genomic and metagenomic sequences containing the PF02036 domain (S9A Fig). The metagenomic sequences were more closely related to WS/DGAT homologs from uncultured bacteria from the Bacteroidota phylum from various marine habitats. The genomic context of the identified sequences contained genes coding for, besides WS/DGAT homologs, a putative 1-acyl-sn-glycerol-3-phosphate acyltransferase/hydrolase, related to neutral lipid biosynthesis (S9B Fig).

## Discussion

In this study, we report a remarkably high relative abundance of sequences containing a PF03007 domain in Antarctic and Subantarctic sediment metagenomes, indicating a high potential for WS/DGAT-dependent TAG and/or WE biosynthesis in these microbial communities. Furthermore, high relative abundances of WS/DGAT homolog sequences were also observed in marine sediment metagenomes from other geographic locations, in agreement with those found in Antarctic and Subantarctic sediments. Sediments contain highly diverse microbial communities that play key roles in global ocean processes, although their metabolic potential is still poorly understood [65, 66]. As marine sediments cover approximately 70% of the Earth surface [67, 68], this capability might be widespread. Seawater microbial communities, on the other hand, presented lower bacterial (WS/DGAT dependent) WE and/or TAG biosynthesis potential. It is important to notice, however, that TAG seems to be an important sunlight driven storage compound in eukaryotic nanophytoplankton, but this process is catalyzed by enzymes not covered in this study, DGAT1 and DGAT2 [69].

Microbial communities from marine sediments and seawater share < 10% of their bacterial types (defined at 97% identity in 16S rRNA gene fragments [70]), and are subjected to different environmental constraints [68]. In a recent work, Rodríguez-Gijón and collaborators [71] reported that microbial communities from the Baltic Sea, a brackish environment, were remarkably distinct in its pelagic and benthic realms, differing not only in their taxonomy, but also in their genome size and metabolic potential. Copiotrophic microorganisms, often presenting a rapid growth and a broad substrate utilization, are abundant in nutrient rich environments such as coastal sediments [72, 73]. In contrast, oligotrophic species predominate in open-ocean waters, which are often nutrient-deficient, reaching lower cell densities and growth rates [73–75]. Nutrient availability is one of the main drivers in the structuring of microbial communities [72]. The higher abundance of bacteria with the potential to synthesize TAG and/or WE in marine sediments relative to seawater could be related to the nutritional conditions of these niches or micro-niches [76]. Storage compounds have been proposed to increase survival through different mechanisms, including the adaptation to fluctuations and unbalances in resource availability, reducing the availability of the resources for competing species, and having a protective effect against various stressors [3]. Interestingly, when analyzing the metagenomes from free-living and particle-attached fractions of deep ocean seawater samples (Malaspina expedition, [77]), the relative abundance of WS/DGAT homologs was higher in particle-attached than in free living communities. In marine sediments, the microbial communities also present mostly a particle-attached lifestyle [78]. These results suggest that the potential for TAG and/or WE biosynthesis in the microbial communities could be associated to an immobilized lifestyle/higher nutrient availability such as those predominant in sediments and other particle-attached communities, rather than particular conditions predominant in the sediments.

The WS/DGAT homologs identified in Antarctic and Subantarctic sediments were mostly assigned to various classes of the Pseudomonadota and Actinomycetota phyla, which include members known for their WE and/or TAG capability [2]. Bacteroidota and Acidobacteriota, phyla in which the biosynthesis and accumulation of these neutral lipids remain unexplored, were represented at lower abundances. The number of WS/DGAT homolog genes per genome varies in different taxonomic groups and even among species, and therefore the predominance of taxa with low or high *ws/dgat* gene copy number in the sediment microbial community will influence the relative abundance of these sequences in the metagenomes. Even with these limitations, when comparing the relative abundances of WS/DGAT homolog sequences assigned to different taxa with the microbial community structure of the same subtidal or intertidal sediment samples, not only these taxa were abundant in the community, but they also followed a similar trend [28, 30]. For instance, WS/DGAT sequences assigned to the Actinomycetota phylum were more abundant in OR07 metagenome than in subtidal sediment metagenomes, which is in agreement with the abundance of members of this phylum in the overall bacterial community (2.8 ± 2.6% in subtidal sediment samples, [28]; 14.5% in OR07 intertidal sediment sample, M. Lozada, personal communication). Similarly, members of the Deltaproteobacteria class were more abundant in Subantarctic sediments, while Flavobacteriia was more abundant in Antarctic sediments [28], as it was the case in the taxonomic profile of WS/DGAT homolog sequences. Differences in the relative abundance of homologs identified in Antarctic and Subantarctic sediments were also observed at the OPU level. Distanced almost 1,000 km, Ushuaia Bay and Potter Cove present differences in average seawater temperature, salinity and anthropogenic impact [28, 30, 35], which could be contributors in the selection of specific microbial populations. Furthermore, a potential dispersal barrier of the Antarctic Circumpolar Current is present between both environments [79]. For instance, OPUs significantly more abundant in Potter Cove included sequences that share high identity values with WS/DGAT homologs identified in *Psychrobacter* spp. (> 90% sequence identity at the amino acid level, OPU1, 15 and 104) from Arctic free-living and host-associated environments [80]. On the other hand, sequences from OPU4 were detected at high relative abundances in all subtidal sediment samples, and shared high identity values with sequences identified in other marine sediments from the Southern Hemisphere [81], suggesting a broad geographic distribution of organisms containing these genes.

The ability of Gammaproteobacteria to produce WE and/or TAG has been reported in several well-known genera, such as *Marinobacter* [11, 63, 82, 83], *Acinetobacter* [8, 84] and *Alcanivorax* [20, 85, 86]. This study showed that sequences that were abundant in the marine sediments were mostly affiliated with genera of Gammaproteobacteria yet to be characterized for their capability to accumulate TAG and/or WE. For instance, sequences assigned to the *Halioglobus* genus (Cellvibrionales order) were abundant in the subtidal sediment metagenomes, and this capability can also be observed in the genomes of members of this genus from strains isolated from different marine habitats [51, 87–89], which contain a high *ws/dgat* gene copy number. Similarly, WS/DGAT homolog sequences closely related to those from *A*. *xantha* DSM 24032 (Arenicellales order), isolated from marine sediments from the Sea of Japan [90], were identified in the intertidal sediment metagenome. Interestingly, the WS/DGAT homolog sequence 136191, from scaffold S1-7808, shared high identity (> 90% at the protein level) with sequences CR-521 (KM114034) and BB-121 (KM114026) identified in intertidal sediments from a site approximately 1,350 km north of Ushuaia Bay [26], suggesting a stable and a wide presence of these potentially TAG-accumulating microorganisms along Patagonian coasts (Argentina). Regarding the *Psychrobacter* (Moraxellales order) genus, which was abundant in Antarctic sediments, WE accumulation has been studied in *P*. *cryohalolentis* K5, and its WS/DGAT enzyme showed high specificity for C14-CoA, as well as for shorter acyl-CoA molecules [10].

Potential reservoirs of novel WS/DGAT enzymes were also detected in other classes of Pseudomonadota, including those affiliated to Alphaproteobacteria and Betaproteobacteria, although with lower relative abundances. The occurrence of WS/DGAT homolog sequences in some members of these classes has been reported by Kalscheuer and collaborators [91], and a more up to date revision revealed that the proportion of genomes containing WS/DGAT homolog sequences was similar in organisms from these classes, and in members of the more extensively characterized Gammaproteobacteria. Their ability to synthesize TAG, WE or PHA remains mostly unknown, with the exception of *Sphingomonas* sp. EGY1 from contaminated soil, which accumulates intracellular inclusions when growing under nitrogen limitation, with TAG as their main component [92]. WS/DGAT sequences related to those identified in members of the Sphingomonadales and Rhodobacterales orders were identified in the metagenomes analyzed in this study, which include marine hydrocarbon-degrading representatives [93]. Two scaffolds affiliated to *Sphingomonadaceae* contained WS/DGAT homolog sequences sharing high identity values with sequences from *P*. *halotolerans* JK6. Furthermore, the genomic contexts of both scaffolds resemble those found in the *Sphingorhabdus* strain. These results may indicate that closely related bacteria could be present in the Subantarctic sediments. In a similar context, the results of this study suggest that some members of Betaproteobacteria in marine sediments could also have the ability to accumulate WE and/or TAG. This is the case of sequences assigned to the Burkholderiales order, such as those included in scaffolds that were closely related to *Rhodoferax ferrireducens* T118, isolated from marine sediments [94], and *Ideonella* sp. A288, isolated from a floodplain area containing iron mineral [95].

The biosynthesis and accumulation of TAG in Actinomycetota has been extensively studied in soil organisms such as *R*. *opacus* PD630 [96] and *S*. *coelicolor* [12] as well as in pathogenic mycobacteria [97], but this capability has not been studied in marine Actinomycetota. A PCR-based analysis identified WS/DGAT homolog sequences related to those from Actinomycetota in soil samples, but not in coastal sediment samples [26]. At the class level, the Actinomycetes class was dominant in WS/DGAT homologs from subtidal sediment metagenomes, while in OR07 metagenome the homolog sequences assigned to Acidimicrobiia were more abundant. Acidimicrobiia is a deep-rooted lineage in the Actinomycetota phylum that includes difficult to culture organisms, present at high abundances in coastal and deep sea sediments [98–101]. *Ilumatobacter* was the main taxonomic assignment of WS/DGAT homologs at the genus level from this class, which was abundant in the intertidal sediment metagenome and in the dataset of Antarctic sediments. Few genomes are currently available from members of this genus, which carry up to five *ws/dgat* genes. From the same class, homolog sequences related to ‘*Ca*. Microthrix’ were also identified in intertidal sediments of Ushuaia Bay and in Antarctic sediments. Remarkably, the two available genomes from this genus to date contain ten WS/DGAT homolog sequences each. ‘*Ca*. Microthrix’ spp. have been identified in wastewater treatment plants around the world, and the capability to accumulate TAG has been confirmed [102]. Within the Actinomycetes class, this study identified WS/DGAT homolog sequences related to those present in well-known lipid-accumulating actinobacteria, such as *Rhodococcus*, *Mycobacterium*, and *Streptomyces* (reviewed in [5].

Another focus of this study was the analysis of the genomic contexts of the WS/DGAT homolog sequences identified in the sediments, and the shared synteny of the gene clusters. The metagenomic library dataset (OR07), although less faithfully representing the genuine microbial diversity in the community under study, allowed, based on the longer length of its scaffolds, the analyses of the genomic contexts associated with the sequences of interest. By examining the presence of gene clusters within closely related sequences, we identified a conserved gene arrangement harboring sequences related to the TAG biosynthesis pathway extended among Actinomycetota phylum, and the Alphaproteobacteria and Gammaproteobacteria classes. The conserved gene arrangement included a complete putative Kennedy pathway. Furthermore, in some Actinomycetota, Alphaproteobacteria and Gammaproteobacteria, the gene cluster included a typical 1-acyl-sn-glycerol-3-phosphate acyltransferase (AGPAT) located next to the WS/DGAT homolog and an AGPAT containing an extra domain related to haloacid dehalogenase-like hydrolase activity (HAD_like superfamily, PF012710). During TAG biosynthesis, the removal of the phosphate from the 1,2-diacylglycerol 3-phosphate molecule, to provide the diacylglycerol substrate for the WS/DGAT, is catalyzed by the phosphatidic acid phosphatase (PAP) activity [103, 104]. Nevertheless, no sequences with homology to PAP type-2 genes (PF01569) were identified in the proximity of the PF03007-containing sequences. On this regard, proteins containing domains belonging to the HAD-type hydrolase family have already been suggested to be involved in the generation of the diacylglycerol [15]. However, the functionality and the physiological relevance of such enzymes or that of these PlsC-like proteins containing both domains (AGPAT-HAD) remain to be elucidated.

## Conclusions

Overall, this study demonstrated that marine environments can be considered as a rich source of microorganisms with the capability to synthesize TAG and/or WE. Marine habitats are challenging environments for bacteria for their frequent fluctuations in nutrient availability. Storage lipids can mitigate nutrient imbalances across time to support survival through unfavorable conditions. However, WE and TAG could play different roles in the physiology of microorganisms in marine environments, which could have developed different biosynthetic machineries for the production of these compounds. Our knowledge of WE/TAG biosynthesis in bacteria is largely limited to few cultured species, not representative of the complex communities present in the environment. This study contributes to expanding our knowledge of ecologically-relevant microorganisms with the potential ability to produce these compounds, in remote Antarctic and Subantarctic environments. Further studies are necessary to empirically verify the synthesis of WE and/or TAG in these marine microorganisms, since a sequence-based inference of the biosynthetic capability cannot guarantee that the microorganisms produce these compounds. This study describes the sequence diversity of putative WS/DGAT sequences from marine microorganisms, which can be the basis for exploring structure-function relationships and substrate specificities of WS/DGAT enzymes, and for their application in biotechnological processes. Finally, results of this study could contribute to integrate storage bacterial physiology into microbial ecology in marine environments.

## Supporting information

S1 Table Sampling sites, metagenomes and number of identified WS/DGAT homolog sequences. (PDF)

S2 Table Pfam domains used to calculate the relative abundance of putative DGAT sequences in the metagenomes. (PDF)

S3 Table Number of copies of WS/DGAT homolog sequences in genomes of Bacteria and Archaea. (PDF)

S4 Table ID of the metagenomes included in this study and study names and DOIs (IMG/M system). (PDF)

S5 Table OPUs of WS/DGAT homolog sequences showing significant differences in their relative abundance between Antarctic (ANT01-ANT06) and Subantarctic (ARG01-ARG06) sediment metagenomes. (PDF)

S6 Table WS/DGAT homolog sequences identified in OR07 metagenome. (PDF)

S7 Table Pfam domains identified in the deduced amino acid sequences of genes located in the analyzed genomic contexts. (PDF)

S1 Fig Relative abundance of putative WS/DGAT sequences in marine metagenomes. (A) Relative abundance of putative WS/DGAT sequences in different environmental matrices. On top of each bar, the number of analyzed metagenomes is indicated, and the bars indicate the average value and the standard deviation of the ratio between the relative abundance of WS/DGAT homolog sequences and the relative abundance of twelve single-copy genes. (B) Relative abundance of putative WS/DGAT sequences in deep ocean samples (2000–4000 m depth) from the Global Malaspina Expedition (Salazar et al. 2016), and water temperature. The metagenome IDs (IMG/M system, https://img.jgi.doe.gov/) used in the analysis are indicated in S4 Table.
(PDF)

S2 Fig Relative abundance of the most abundant taxonomic assignment at the genus level of WS/DGAT homolog sequences (estimated sequences). (A) Gammaproteobacteria class. (B) Actinobacteria phylum. The sequences were analyzed using blastp (nr, 100 hits), and Megan6 LCA algorithm was used to classify each sequence at the class level. In the case of sequences from the assembled metagenome of ARG01-ARG06, values were corrected based on gene copy number, as indicated in the IMG/M system for sequences of the assembled metagenomes.
(PDF)

S3 Fig Heatmap showing the 25 most abundant OPUs in the dataset (estimated values) including metagenomes from Subantarctic and Antarctic sediments. The dataset contained 1,022 OPUs containing ≥ 10 sequences. Scale is arbitrary and goes from light (low relative abundance) to dark (high relative abundance). On the right, phylum or class of the representative sequence in the cluster is indicated. The symbol # next to the OPU number indicates clusters showing significantly differences between Antarctic and Subantarctic sediment metagenomes (Wilcoxon Rank Sum test corrected for multiple testing, as implemented in the R-script ANCOM).
(PDF)

S4 Fig Boxplot showing the relative abundance of clusters containing sequences identified in metagenomes of subtidal sediments. ANT01-06, gray; ARG01-06, black. Only clusters containing more than 10 estimated sequences and including sequences identified in intertidal sediments metagenome (OR07, right) are shown. Asterisk indicates > 1 sequence from OR07 in the cluster. On the right of the boxplot, colors indicate phylum or class, which are also indicated next to the sequence name: Alph cl, Alphaproteobacteria class; Bet cl, Betaproteobacteria class; Gam cl, Gammaproteobacteria class; Del cl, Deltaproteobacteria class; Act ph, Actinomycetota phylum; Act cl, Actinomycetes class; Aci cl, Acidimicrobiia class; Bac ph, Bacteroidota phylum.
(PDF)

S5 Fig Phylogenetic analysis of sequences assigned to the Actinomycetota phylum. Maximum likelihood tree of WS/DGAT homolog sequences assigned to the Actinomycetota phylum identified in the metagenomic dataset of intertidal sediments of Ushuaia Bay (OR07). Names of metagenomic sequences are shown in red, and related sequences from the IMG/M and NCBI databases, in black. The tree was built in MEGA X (Kumar et al. 2018). Bootstrap values are percent of 100 replications.
(PDF)

S6 Fig Phylogenetic analysis and genomic context of sequences assigned to the Betaproteobacteria class. (A) Maximum-Likelihood tree of WS/DGAT homolog sequences assigned to the Betaproteobacteria class, identified in the metagenomic dataset of intertidal sediments (OR07, in red) and related sequences from public databases (in black). GEN, sequence identified in a genome; MAG, sequence identified in a metagenome-assembled genome. Bootstrap values higher than 50% based on 100 replicates are shown. The box indicates the sequences for which the genomic context and shared synteny is shown below. (B) Genomic context and shared synteny of the cluster indicated above. Gene clusters, including WS/DGAT homolog sequences and other putative genes related to fatty acid metabolism pathway are shown. Gray gradient represents % identity at the nucleotide level between scaffolds.
(PDF)

S7 Fig Phylogenetic analysis and genomic context of sequences assigned to the Gammaproteobacteria class. (A) Maximum-Likelihood tree of WS/DGAT homolog sequences assigned to Gammaproteobacteria class, identified in the metagenomic dataset of intertidal sediments (OR07, in red) and related sequences from public databases (in black). GEN, sequence identified in a genome; MAG, sequence identified in a metagenome-assembled genome. Bootstrap values higher than 50% based on 100 replicates are shown. The box indicates the sequences for which the shared synteny is shown below. (B) Genomic context and shared synteny of the cluster indicated above. Gene clusters, including WS/DGAT homolog sequences and other putative enzymes of the Kennedy pathway are shown. (C) Representative gene clusters including WS/DGAT homolog sequences and other putative genes related to fatty acid metabolism pathway, and their shared synteny. Gray gradient represents percent identity at the nucleotide level between scaffolds.
(PDF)

S8 Fig Phylogenetic analysis of sequences assigned to the Deltaproteobacteria class. Maximum-Likelihood tree of WS/DGAT homolog sequences assigned to Deltaproteobacteria class, identified in the metagenomic dataset of intertidal sediments (OR07, in red) and related sequences from public databases (in black). GEN, sequence identified in a genome; MAG, sequence identified in a metagenome assembled genome. Bootstrap values higher than 50% based on 100 replicates are shown.
(PDF)

S9 Fig Phylogenetic analysis and genomic context of sequences assigned to the Bacteroidota phylum. (A) Maximum-Likelihood phylogenetic tree of WS/DGAT homolog sequences assigned to Bacteroidota identified in the metagenomic dataset of intertidal sediments (OR07, in red) and sequences from public databases from genomes of members of the Bacteroidota phylum containing a putative SCP-2 domain at the C-term (in black; ID with numbers, IMG/M; numbers and letters, NCBI). Only unique sequences are included in the tree. IS: isolate; MAG: metagenome assembled genome; Red: sequences identified in this study. Bootstrap values higher than 50% based on 100 replicates are shown. (B) Genomic context and shared synteny of scaffolds containing the WS/DGAT homologs included in the rectangle in the phylogenetic tree. Gray/black regions correspond to the percentage identity at the nucleotide level shown on the right.
(PDF)
