# Supplementary material for: High wax ester and triacylglycerol biosynthesis potential in coastal sediments of Antarctic and Subantarctic environments
Source: PLoS One. 2023 Jul 17;18(7):e0288509. doi: 10.1371/journal.pone.0288509 (PMC10351704; doi:10.1371/journal.pone.0288509)
Supplement: S1 Table — (PDF) [file pone.0288509.s001.pdf]

S1 Table. Sampling sites, metagenomes and number of identified WS/DGAT homolog sequences.

Sampling region	Sampling site		Environmental matrix	Sample name/ metagenome	Total hydrocarbon (µg/g wet sediment)	CDS assembled metagenomes	CDS unassembled metagenomes	Number of identified homolog sequences	Estimated number of homolog sequences
Ushuaia Bay, Tierra del Fuego Island, Argentina	MC	54° 48.656' S 68° 17.731' W	subtidal sediments	ARG01	0.100	139,000	23,700,000	1,879	2,459
				ARG02	0.102	174,000	76,700,000	7,351	7,946
				ARG03	0.091	474,000	69,500,000	3,877	5,066
	OR	54° 48.256' S 68° 17.296' W	subtidal sediments	ARG04	0.383	279,000	46,500,000	1,471	1,592
				ARG05	0.652	713,000	96,200,000	6,198	8,014
				ARG06	5.484	187,000	48,900,000	3,439	3,648
	OR	54º 48.278' S 68º 17.394' W	Intertidal sediments	OR07	78.29*	682,972	-	164	-
Potter Cove, 25 de Mayo Island, Antarctica	S1	62° 13.833' S 58° 39.367' W	subtidal sediments	ANT01	0.115	1,110,161	70,553,713	5,847	8,111
				ANT02	0.018	972,350	58,166,042	4,904	6,765
				ANT03	0.197	278,541	34,680,166	2,965	3,306
	S2	62° 13.917' S 58° 39.300' W	subtidal sediments	ANT04	0.048	657,494	32,497,297	1,686	2,650
				ANT05	0.038	467,252	59,120,163	4,449	5,932
				ANT06	0.051	100,652	75,922,867	3,972	4,353

CDS, protein coding sequences; THC, total hydrocarbon concentrations. *Expressed as µg/g dry sediment.
