# Supplementary material for: High wax ester and triacylglycerol biosynthesis potential in coastal sediments of Antarctic and Subantarctic environments
Source: PLoS One. 2023 Jul 17;18(7):e0288509. doi: 10.1371/journal.pone.0288509 (PMC10351704; doi:10.1371/journal.pone.0288509)
Supplement: S2 Table — (PDF) [file pone.0288509.s002.pdf]

S2 Table: Pfam domains used to calculate the relative abundance of putative DGAT sequences in the metagenomes

Acc. number	Name
PF00189	Ribosomal protein S3, C-terminal domain
PF00252	Ribosomal protein L16p/L10e
PF00453	Ribosomal protein L20
PF00542	Ribosomal protein L7/L12 C-terminal domain
PF00831	Ribosomal L29 protein
PF00886	Ribosomal protein S16
PF01016	Ribosomal L27 protein
PF01196	Ribosomal protein L17
PF01245	Ribosomal protein L19
PF01250	Ribosomal protein S6
PF01281	Ribosomal protein L9, N-terminal domain
PF01649	Ribosomal protein S20
