# Supplementary material for: High wax ester and triacylglycerol biosynthesis potential in coastal sediments of Antarctic and Subantarctic environments
Source: PLoS One. 2023 Jul 17;18(7):e0288509. doi: 10.1371/journal.pone.0288509 (PMC10351704; doi:10.1371/journal.pone.0288509)
Supplement: S3 Table — (PDF) [file pone.0288509.s003.pdf]

S3 Table. Number of copies of WS/DGAT homolog sequences in genomes of Bacteria and Archaea.

Group/Domain	Phylum	Class	Genus	Number of genomes in the database	Number (percentage) of genomes containing WS/DGAT homolog sequences	WS/DGAT homolog sequences per genome (average ± standard deviation)	Maximum number of WS/DGAT homolog sequences per genome
FCB group	Bacteroidota			4855	143 (2.95 %)	1.07 ± 0.41	5
		Flavobacteriia		1852	40 (2.16 %)	1.03 ± 0.16	2
		Cytophagia		462	2 (0.43 %)	1	1
	Gemmatimonadota			68	2 (2.94 %)	1	1
	"*Ca*. Hydrogenedentes"			15	1 (6.67 %)	1	1
	Rhodothermota			9	8 (88.89 %)	1.11 ± 0.33	2
	Pseudomonadota			40,131	4415 (11 %)		
		Alphaproteobacteria		7893	738 (9.35 %)	1.28 ± 0.75	12
		Betaproteobacteria		5367	550 (10.25 %)	1.53 ± 0.95	11
		Gammaproteobacteria		26,578	3127 (11.77 %)	1.42 ± 1.32	17
			Paraglaciecola	14	3 (21.43 %)	5.33 ± 2.52	8
			Halioglobus	7	7 (100%)	10.3 ± 5.3	17
			Oceanicoccus	2	1 (50%)	1	1
			Pseudomonas	2782	65 (2.52 %)	1.2 ± 1.15	10
			Psychrobacter	75	72 (96 %)	1.14 ± 0.35	2
		Zetaproteobacteria		233	0		
		Acidithiobacillia		41	0		
		"*Ca*. Lambdaproteobacteria"		5	0		
		"*Ca*. Muproteobacteria"		14	0		
	delta/epsilon subdivisions	Deltaproteobacteria		950	144 (15.16 %)	1.45 ± 1.24	8
		Epsilonproteobacteria		1902	0		
Terrabacteria group	Cyanobacteriota			1743	3 (0.17 %)	1	1
	Chloroflexota			503	32 (6.36 %)	1.38 ± 0.75	4
	Armatimonadota			39	1 (2.56 %)	2	2
	Actinomycetota			13,925	7999 (58.77 %)		
		Actinomycetes		13,530	7960 (58.83 %)	7.65 ± 5.20	31

			Mycobacterium	2980	2976 (99.87 %)	13.13 ± 2.22	31
		Acidimicrobiia		53	14 (26.42 %)	4.14 ± 3.16	10
			Ilumatobacter	5	5 (100 %)	3.6 ± 1.14	5
			"*Ca.* Microthrix"	2	2 (100 %)	10	10
		Thermoleophilia		21	19 (90.48 %)	4.05 ± 2.30	9
		Nitriliruptoria		7	6 (85.71 %)	3 ± 1.26	4
		Rubrobacteria		15	1 (6.67 %)	3	3
		Coriobacteriia		299	0		
	Acidobacteriota			198	17 (8.58 %)	1.41 ± 0.62	3
	Planctomycetota			445	1 (0.22 %)	1	1
	Spirochaetota			1062	1 (0.09 %)	3	3
Archaea domain				2156	14 (0.65 %)	1	1

Information retrieved from the IMG/M system on March 15, 2022
