# Supplementary material for: High wax ester and triacylglycerol biosynthesis potential in coastal sediments of Antarctic and Subantarctic environments
Source: PLoS One. 2023 Jul 17;18(7):e0288509. doi: 10.1371/journal.pone.0288509 (PMC10351704; doi:10.1371/journal.pone.0288509)
Supplement: S4 Table — (PDF) [file pone.0288509.s004.pdf]

S4 Table: ID of the metagenomes included in this study and study names and DOIs (IMG/M system)

Marine habitat	Metagenome ID
Belowground wetlands	3300004009, 3300003995, 3300004019, 3300004020, 3300004023, 3300004029, 3300004026, 3300004030, 3300004008, 3300004028, 3300004003, 3300004005, 3300004021, 3300004004, 3300004001, 3300004018, 3300004006, 3300003990, 3300004014, 3300004000, 3300004027, 3300004266, 3300004011, 3300003991, 3300004015, 3300004012, 3300004013, 3300003996, 3300003997, 3300003993, 3300003999, 3300003992, 3300003987, 3300003998, 3300003989, 3300003988, 3300004007, 3300004010, 3300004002, 3300004056, 3300004055, 3300004064, 3300004065, 3300005216, 3300004063, 3300004066, 3300004067, 3300004071, 3300004061, 3300004070, 3300004145, 3300004052, 3300004050, 3300004051, 3300004049, 3300004048, 3300004146, 3300004057, 3300004062, 3300004058, 3300004047, 3300005218, 3300004022, 3300003994, 3300004025, 3300004024, 3300005183, 3300005206, 3300005204, 3300005213, 3300005205, 3300005182, 3300005215, 3300005214, 3300005208, 3300005219, 3300004072, 3300004078, 3300004079, 3300004147, 3300004073, 3300004074, 3300004148, 3300004075, 3300004076, 3300004150, 3300004077, 3300000840, 3300000094, 3300000917, 3300000786, 3300000030, 3300000031, 3300000854, 3300000894
Sediments (this study)	3300000125, 3300000131, 3300000121, 3300000122, 3300000242, 3300000118, 3300000119, 3300000136, 3300000135, 3300000129, 3300000132, 3300000123
Other sediments	3300000133, 3300000127, 3300000128, 3300000120, 3300000130, 3300000243, 3300000792, 3300000134, 3300000124, 3300000241, 3300000126, 3300000511, 3300001136, 3300000444, 3300000403, 3300000790, 3300000515, 3300000426, 3300000409, 3300000892, 3300000463, 3300005601, 3300005612, 3300005600, 3300005590, 3300005609, 3300005920, 3300005588, 3300005589, 3300001752, 3300001854, 3300001751, 3300001855, 3300001753, 3300001706, 3300003541, 3300003432, 3300001279, 3300009499, 3300009149, 3300009529, 3300009788, 3300009488, 3300009139, 3300009030, 3300009102, 3300009150, 3300009528
Deep ocean, particle associated	3300001054, 3300000967, 3300001091, 3300001122, 3300001013, 3300000984, 3300001126, 3300000946, 3300001578, 3300000994, 3300001006, 3300001572, 3300000975, 3300001118, 3300000978, 3300000981, 3300001016, 3300001571, 3300001114, 3300001115, 3300000971, 3300001051, 3300001113, 3300001127, 3300001585, 3300000982
Deep ocean, free-living	3300001060, 3300000966, 3300001125, 3300001574, 3300001106, 3300001068, 3300000950, 3300001063, 3300000995, 3300001579, 3300001581, 3300001058, 3300001569, 3300000974, 3300001129, 3300001046, 3300001573, 3300000999, 3300001586, 3300001582, 3300001576, 3300001049, 3300001121, 3300001587, 3300001575,3300001123

Oil-seep	3300001750, 3300001685, 3300001749, 3300002180, 3300002225
Microbial mat of gas chimney	3300009702
Methane seep	3300008252, 3300008227, 3300008251, 3300008250, 3300008226, 3300008222, 3300008249, 3300008253, 3300008224, 3300008254, 3300008225, 3300008223
Seawater, pelagic or coastal	3300002753, 3300002525, 3300002527, 3300002751, 3300002754, 3300002533, 3300002524, 3300002537, 3300003247, 3300003428, 3300003268, 3300003269, 3300003270, 3300003271, 3300003263, 3300003264, 3300003265, 3300007363, 3300006863, 3300007234, 3300006637, 3300006027, 3300007236, 3300006805, 3300006037, 3300006029, 3300006803, 3300007229, 3300007231, 3300006030, 3300006641, 3300006917, 3300006875, 3300006025, 3300006874, 3300006867, 3300006869, 3300006026, 3300006870, 3300008012, 3300006868, 3300009002, 3300009080, 3300007555, 3300007552, 3300007553, 3300007554, 3300007557, 3300007692, 3300007681, 3300009024, 3300009086, 3300009003, 3300009079, 3300008999, 3300007558, 3300007559, 3300009026, 3300009059, 3300008996, 3300007655, 3300009058, 3300007718, 3300007647, 3300007543, 3300007636, 3300007708, 3300007622, 3300009056, 3300009051, 3300007544, 3300007639, 3300007670, 3300007627, 3300007661, 3300007625, 3300007716, 3300007620, 3300007653, 3300007546, 3300007621, 3300007547, 3300007545, 3300007624, 3300007642, 3300007549, 3300007548, 3300007550, 3300010312, 3300007551, 3300009052, 3300009141, 3300008961, 3300009142, 3300008964, 3300008995, 3300008950, 3300010309, 3300008052, 3300009057, 3300007618, 3300007632, 3300007617, 3300007629, 3300007634, 3300007658, 3300007644, 3300007706, 3300007630, 3300007651, 3300007637, 3300007981, 3300007962, 3300009055, 3300009050, 3300007665, 3300009049, 3300007667, 3300007560, 3300007649, 3300007562, 3300007561, 3300007590, 3300007585, 3300007597, 3300007593, 3300007603, 3300007600, 3300007606, 3300008021, 3300009054, 3300007715, 3300006484, 3300005941, 3300005942, 3300010368, 3300010316, 3300010370, 3300010354, 3300010299, 3300010318, 3300010297, 3300010300, 3300010296, 3300005567, 3300005608, 3300005658, 3300005551, 3300005599, 3300005594, 3300005606, 3300005593, 3300005592, 3300006166, 3300005596, 3300005433, 3300005595, 3300005516, 3300005597, 3300005422, 3300005510, 3300005423, 3300005424, 3300005509, 3300005408, 3300005426, 3300005429, 3300005432, 3300005425, 3300005398, 3300005401, 3300005404, 3300005603, 3300005604, 3300005427, 3300005605, 3300005430, 3300005402, 3300005431, 3300005520, 3300005428, 3300005521, 3300005522, 3300005508, 3300005400, 3300005514, 3300005523, 3300005399, 3300000101, 3300000116, 3300000115, 3300000117, 3300003617, 3300003427, 3300003908, 3300003909, 3300003410, 3300003409, 3300003216, 3300003346, 3300003621, 3300003345, 3300003596, 3300003580, 3300004110, 3300005838, 3300004109, 3300004111,

	3300003478, 3300003599, 3300003615, 3300003600, 3300003620, 3300003498, 3300003494, 3300003496, 3300003500, 3300003501, 3300003495, 3300003492, 3300003588, 3300003592, 3300003589, 3300003585, 3300003591, 3300003619, 3300003590, 3300003583, 3300003582, 3300003584, 3300003587, 3300003618, 3300003581, 3300003593, 3300003594, 3300003586, 3300003602, 3300003601, 3300003595, 3300004280, 3300004279, 3300004274, 3300004273, 3300004278, 3300004276, 3300004277, 3300006306, 3300002178, 3300002177, 3300002221, 3300002526, 3300002528, 3300002176, 3300002956, 3300002913, 3300002919, 3300002955, 3300002965, 3300003185, 3300006164, 3300006165, 3300006947, 3300006193, 3300006190, 3300006191, 3300006352, 3300009706, 3300009512, 3300009526, 3300009409, 3300009420, 3300009172, 3300009173, 3300009425, 3300009422, 3300009786, 3300009705, 3300009785, 3300000385, 3300000517, 3300000547, 3300000371, 3300000418, 3300000425, 3300000369, 3300000954, 3300009433, 3300009435, 3300009426, 3300009423, 3300009074, 3300009076, 3300009193, 3300009077, 3300009445, 3300009472, 3300009476, 3300009437, 3300009443, 3300009449, 3300009438, 3300009447, 3300009440, 3300009434, 3300009442, 3300009505, 3300009467, 3300009071, 3300009508, 3300009498, 3300009497, 3300009496, 3300009495, 3300009507, 3300003847, 3300004113, 3300004097, 3300001346, 3300001344, 3300001351, 3300001348, 3300001354, 3300001347, 3300001352, 3300001355, 3300001353, 3300001349, 3300006902, 3300006900, 3300005951, 3300005945, 3300005953, 3300005946, 3300005948, 3300005934, 3300007283, 3300007291, 3300008097
Volcanic	3300002481, 3300002532
Hydrothermal fluid	3300002481, 3300002532, 3300010330, 3300010264, 3300010332
**Award DOI**	**Study name, reference**
https://www.osti.gov/award-doi-service/biblio/10.46936/10.25585/60000748	Aqueous microbial communities from the Delaware River/Bay and Chesapeake Bay under freshwater to marine salinity gradient to study organic matter cycling in a time-series (Campbell 2014)
https://www.osti.gov/award-doi-service/biblio/10.46936/10.25585/60000621	Bacterial and archaeal communities from various locations to study Microbial Dark Matter (Phase II) (Stepanauskas 2013)
https://www.osti.gov/award-doi-service/biblio/10.46936/10.25585/60000616	Deep ocean microbial communities from the Global Malaspina Expedition (Acinas et al. 2021)
https://www.osti.gov/award-doi-service/biblio/10.46936/10.25585/60000930	Deep subsurface and oceanic microbial communities from Witwatersrand Basin, South Africa, and the Canadian and Fennoscandian shields and at the Lost City Hydrothermal Field (Anantharaman et al. 2018)
https://www.osti.gov/award-doi-service/biblio/10.46936/10.25585/60000621	Deep subsurface microbial communities from various oceans to uncover new lineages of life (NeLLi) (Nayfach et al. 2021)
https://www.osti.gov/award-doi-service/biblio/10.46936/10.25585/60007445	Environmental microbial communities from Fremont, CA and La Paraguera, Puerto Rico (Nayfach et al. 2021)
https://www.osti.gov/award-doi-service/biblio/10.46936/10.25585/60000867	Estuarine microbial communities from the Columbia River estuary, to analyze effect of nutrient fluxes, a time series (Simon et al. 2014)

https://www.osti.gov/award-doi-service/biblio/10.46936/10.25585/60000921	Marine archaeal communities from Monterey Bay, CA, that are ammonia-oxidizing (Reji et al. 2020)
https://www.osti.gov/award-doi-service/biblio/10.46936/10.25585/60007348	Marine microbial communities from chronically polluted sediments in four geographic locations (Espínola et al. 2018)
https://www.osti.gov/award-doi-service/biblio/10.46936/10.25585/60007339	Marine microbial communities from Delaware Coast (Ahmed et al. 2021)
https://www.osti.gov/award-doi-service/biblio/10.46936/10.25585/60000795	Marine microbial communities from expanding oxygen minimum zones in the northeastern subarctic Pacific Ocean (Wright et al. 2012)
https://www.osti.gov/award-doi-service/biblio/10.46936/10.25585/60000538	Marine microbial communities from oxygen minimum zone in the mesopelagic equatorial Pacific (Saito et al. 2020)
University of Hawaii	Marine microbial communities from the North Pacific Subtropical Gyre, ALOHA station (Mende et al. 2017)
https://www.osti.gov/award-doi-service/biblio/10.46936/10.25585/60007579	Marine microbial communities from the Santa Barbara Channel oil seeps (Hawley et al. 2014)
https://www.osti.gov/award-doi-service/biblio/10.46936/10.25585/60007622	Marine microbial communities from the Southern Atlantic Ocean affecting the dissolved organic carbon pool (Rinke et al. 2013)
https://www.osti.gov/award-doi-service/biblio/10.46936/10.25585/60001037	Marine microbial communities from the Southern Atlantic ocean transect to study dissolved organic matter and carbon cycling (Hallam 2014)
https://www.osti.gov/award-doi-service/biblio/10.46936/10.25585/60000544	Marine microbial communities from the West Antarctic Peninsula, for metatranscriptomic analysis (Grzymski et al. 2012)
https://www.osti.gov/award-doi-service/biblio/10.46936/10.25585/60000714	Marine microbial communities from western Arctic Ocean (Colatriano et al. 2018)
https://www.osti.gov/award-doi-service/biblio/10.46936/10.25585/60000564	Marine sediment microbial communities from the Atlantic coast under amendment with organic carbon and nitrate (Walsh 2015)
https://www.osti.gov/award-doi-service/biblio/10.46936/10.25585/60007583	Marine sediment microbial communities from White Oak River estuary, North Carolina (Seitz et al. 2016)
https://www.osti.gov/award-doi-service/biblio/10.46936/10.25585/60007671	Methane-oxidizing microbial communities from mesocosms in the Gulf of Mexico and Hudson Canyon, USA (Redmond 2015)
https://www.osti.gov/award-doi-service/biblio/10.46936/10.25585/60000893	Microbial and viral regulation of community carbon cycling across diverse low-oxygen zones (Bertagnolli et al. 2020)
https://www.osti.gov/award-doi-service/biblio/10.46936/10.25585/60000751	Microbial community impact on carbon sequestration in managed wetland Carbon farming (He et al. 2015)
https://www.osti.gov/award-doi-service/biblio/10.46936/10.25585/60000697	Natural and restored wetland microbial communities from the San Francisco Bay, California, USA, that impact long-term carbon sequestration (Theroux et al. 2014)
https://www.osti.gov/award-doi-service/biblio/10.46936/10.25585/60000792	Pelagic marine microbial communities from North Sea (Teeling et al. 2016)
https://www.osti.gov/award-doi-service/biblio/10.46936/10.25585/60001414	Wetland microbial communities from Twitchell Island in the Sacramento Delta (He et al. 2015)

References

Acinas SG, Sánchez P, Salazar G, Cornejo-Castillo FM, Sebastián M, Logares R, Royo-Llonch M, Paoli L, Sunagawa S, Hingamp P (2021) Deep ocean metagenomes provide insight into the metabolic architecture of bathypelagic microbial communities. Communications Biology 4:1-15. https://doi.org/10.1038/s42003-021-02112-2

Ahmed MA, Lim SJ, Campbell BJ (2021) Metagenomes, Metatranscriptomes, and Metagenome-Assembled Genomes from Chesapeake and Delaware Bay (USA) Water Samples. Microbiology Resource Announcements 10:e00262-21. https://doi.org/10.1128/MRA.00262-21

Anantharaman K, Hausmann B, Jungbluth SP, Kantor RS, Lavy A, Warren LA, Rappé MS, Pester M, Loy A, Thomas BC (2018) Expanded diversity of microbial groups that shape the dissimilatory sulfur cycle. The ISME Journal 12:1715-1728. https://doi.org/10.1038/s41396-018-0078-0

Bertagnolli AD, Konstantinidis KT, Stewart FJ (2020) Non-denitrifier nitrous oxide reductases dominate marine biomes. Environmental Microbiology Reports 12:681-692. https://doi.org/10.1111/1758-2229.12879

Campbell B (2014) Biogeochemical cycling links between terrestrial and marine systems. USDOE Joint Genome Institute (JGI), Berkeley, CA (United States). https://doi.org/10.25585/1488110

Colatriano D, Tran PQ, Guéguen C, Williams WJ, Lovejoy C, Walsh DA (2018) Genomic evidence for the degradation of terrestrial organic matter by pelagic Arctic Ocean Chloroflexi bacteria. Communications Biology 1:1-9. https://doi.org/10.1038/s42003-018-0086-7

Espínola F, Dionisi HM, Borglin S, Brislawn CJ, Jansson JK, Mac Cormack WP, Carroll J, Sjöling S, Lozada M (2018) Metagenomic analysis of subtidal sediments from polar and subpolar coastal environments highlights the relevance of anaerobic hydrocarbon degradation processes. Microbial Ecology 75:123-139. https://doi.org/10.1007/s00248-017-1028-5

Grzymski JJ, Riesenfeld CS, Williams TJ, Dussaq AM, Ducklow H, Erickson M, Cavicchioli R, Murray AE (2012) A metagenomic assessment of winter and summer bacterioplankton from Antarctica Peninsula coastal surface waters. The ISME Journal 6:1901-1915. https://doi.org/10.1038/ismej.2012.31

Hallam S (2014) Microbial engines driving organic matter transformations in the dark ocean: an integrated biological and chemical perspective. USDOE Joint Genome Institute (JGI), Berkeley, CA (United States). https://doi.org/10.25585/1488116

Hawley ER, Piao H, Scott NM, Malfatti S, Pagani I, Huntemann M, Chen A, Glavina del Rio T, Foster B, Copeland A (2014) Metagenomic analysis of microbial consortium from natural crude oil that seeps into the marine ecosystem offshore Southern California. Standards in Genomic Sciences 9:1259-1274. https://doi.org/10.4056/sigs.5029016

He S, Malfatti SA, McFarland JW, Anderson FE, Pati A, Huntemann M, Tremblay J, Glavina del Rio T, Waldrop MP, Windham-Myers L (2015) Patterns in wetland microbial community composition and functional gene repertoire associated with methane emissions. MBio 6:e00066-15. https://doi.org/10.1128/mbio.00066-15

Mende DR, Bryant JA, Aylward FO, Eppley JM, Nielsen T, Karl DM, DeLong EF (2017) Environmental drivers of a microbial genomic transition zone in the ocean's interior. Nature Microbiology 2:1367-1373. https://doi.org/10.1038/s41564-017-0008-3

Nayfach, S., Roux, S., Seshadri, R. et al. (2021) A genomic catalog of Earth's microbiomes. Nature Biotechnology 39:499-509. https://doi.org/10.1038/s41587-020-0718-6

Redmond M (2015) Metagenomic sequencing of methane-oxidizing mesocosms from the Gulf of Mexico and Hudson Canyon. USDOE Joint Genome Institute (JGI), Berkeley, CA (United States). https://doi.org/10.25585/1487995

Reji L, Tolar BB, Chavez FP, Francis CA (2020) Depth-differentiation and seasonality of planktonic microbial assemblages in the Monterey Bay upwelling system. Frontiers in Microbiology 11:1075. https://doi.org/10.3389/fmicb.2020.01075

Rinke C, Schwientek P, Sczyrba A, Ivanova NN, Anderson IJ, Cheng J-F, Darling A, Malfatti S, Swan BK, Gies EA (2013) Insights into the phylogeny and coding potential of microbial dark matter. Nature 499:431-437. https://doi.org/10.1038/nature12352

Salazar G, Cornejo-Castillo FM, Benítez-Barrios V, Fraile-Nuez E, Álvarez-Salgado XA, Duarte CM, Gasol JM, Acinas SG (2016) Global diversity and biogeography of deep-sea pelagic prokaryotes. The ISME Journal 10:596-608. https://doi.org/10.1038/ismej.2015.137

Saito MA, McIlvin MR, Moran DM, Santoro AE, Dupont CL, Rafter PA, Saunders JK, Kaul D, Lamborg CH, Westley M (2020) Abundant nitrite-oxidizing metalloenzymes in the mesopelagic zone of the tropical Pacific Ocean. Nature Geoscience 13:355-362. https://doi.org/10.1038/s41561-020-0565-6

Seitz KW, Lazar CS, Hinrichs K-U, Teske AP, Baker BJ (2016) Genomic reconstruction of a novel, deeply branched sediment archaeal phylum with pathways for acetogenesis and sulfur reduction. The ISME Journal 10:1696-1705. https://doi.org/10.1038/ismej.2015.233

Simon HM, Smith MW, Herfort L (2014) Metagenomic insights into particles and their associated microbiota in a coastal margin ecosystem. Frontiers in Microbiology 5:466. https://doi.org/10.3389/fmicb.2014.00466

Stepanauskas R (2013) Microbial Dark Matter project phase II-stepping deeper into unknown territory. USDOE Joint Genome Institute (JGI), Berkeley, CA (United States). https://www.osti.gov/biblio/1241219

Teeling H, Fuchs BM, Bennke CM, Krüger K, Chafee M, Kappelmann L, Reintjes G, Waldmann J, Quast C, Glöckner FO (2016) Recurring patterns in bacterioplankton dynamics during coastal spring algae blooms. eLife 5:e11888. https://doi.org/10.7554/eLife.11888

Theroux S, Hartman W, He S, Tringe S (2014) Microbial diversity and carbon cycling in San Francisco Bay wetlands. Lawrence Berkeley National Lab (LBNL), Berkeley, CA (United States) https://escholarship.org/uc/item/4mg7n68g

Walsh D (2015) Metagenomics of western Arctic Ocean microbial communities. USDOE Joint Genome Institute (JGI), Berkeley, CA (United States) https://genome.jgi.doe.gov/portal/ArcticOcean_MG_C_8_FD/ArcticOcean_MG_C_8_FD.info.html

Wright JJ, Konwar KM, Hallam SJ (2012) Microbial ecology of expanding oxygen minimum zones. Nature Reviews Microbiology 10:381-394. https://doi.org/10.1038/nrmicro2778.
