# Supplementary material for: High wax ester and triacylglycerol biosynthesis potential in coastal sediments of Antarctic and Subantarctic environments
Source: PLoS One. 2023 Jul 17;18(7):e0288509. doi: 10.1371/journal.pone.0288509 (PMC10351704; doi:10.1371/journal.pone.0288509)
Supplement: S5 Table — (PDF) [file pone.0288509.s005.pdf]

S5 Table. OPUs of WS/DGAT homolog sequences showing significant differences in their relative abundance between Antarctic (ANT01-ANT06) and Subantarctic (ARG01-ARG06) sediment metagenomes.

OPU	meanANT	sdANT	meanARG	sdARG	length	ID	Scientific name of first hit Blastp (max. score)	Accession number % identity % coverage
OPU0	**0.0654**	**0.0312**	0.0034	0.0018	465aa	KGI_S1_ANT03_95mDRAFT_10002395	Gammaproteobacteria bacterium (marine sediment metagenome, China: Bohai Sea)	NCF50312 74.73 % id 99 % cov
OPU1	**0.0551**	**0.0410**	0.0000	0.0000	475aa	KGI_S1_ANT02_95mDRAFT_100099224	*Psychrobacter* sp. NG25 (isolate, seawater, Arctic Ocean)	WP_194196028 93.05 % id 100 % cov
OPU3	**0.0572**	**0.0436**	0.0029	0.0019	613aa	KGI_S1_ANT01_95mDRAFT_100182042	*Arcticiflavibacter luteus* (isolate, arctic intertidal sand sample from the littoral zone of Kongsfjorden)	WP_194507792 84.18 % id 100 % cov
OPU6	**0.0627**	**0.0350**	0.0002	0.0002	510aa	KGI_S1_ANT01_95mDRAFT_100086573	Xanthomonadales bacterium (beach sand metagenome, Australia: Middle Park Beach, Port Phillip Bay site B)	NNK99515 70.78 % id 100 % cov
OPU8	**0.0549**	**0.0217**	0.0013	0.0016	483aa	KGI_S1_ANT01_95mDRAFT_100162061	Xanthomonadales bacterium (marine sediment metagenome, New Zealand: Hikurangi Margin)	NOR19411 90.57 % id 98 % cov
OPU12	**0.0230**	**0.0314**	0.0008	0.0010	333aa	KGI_S1_ANT02_95mDRAFT_100574981	Xanthomonadales bacterium (beach sand metagenome, Australia: Middle Park Beach, Port Phillip Bay site B)	NNK99515 72.07 % id 100 % cov
OPU15	**0.0138**	**0.0108**	0.0000	0.0000	482aa	KGI_S1_ANT01_95mDRAFT_100122882	*Psychrobacter* sp. P11G5 (isolate, ascidian Botryllus sp., Norway)	WP_068034115 97.20 % id 96 % cov
OPU25	**0.0160**	**0.0180**	0.0004	0.0007	401aa	KGI_S1_ANT01_95mDRAFT_100019749	Acidimicrobiales bacterium (beach sand metagenome, Australia: Middle Park Beach, Port Phillip Bay)	NNC80367 86.00 % id 99 % cov
OPU36	**0.0194**	**0.0122**	0.0005	0.0008	485aa	KGI_S1_ANT01_95mDRAFT_100027476	Acidimicrobiales bacterium (beach sand metagenome, Australia: Middle Park Beach, Port Phillip Bay)	NNC79469 **53.29 % id** 96 % cov
OPU41	**0.0038**	**0.0021**	0.0004	0.0004	608aa	KGI_S2_ANT04_2345mDRAFT_12554931	*Ilumatobacter nonamiensis* (isolate, soil)	WP_083914852 85.32 % id 100 % cov

OPU44	**0.0062**	**0.0038**	0.0010	0.0012	89aa	KGI_S2_ANT05_2345mDRAFT_12894631	*Zhongshania aliphaticivorans* (isolate, tidal flat)	WP_159238949<br>78.65 % id<br>100 % cov
OPU48	**0.0295**	**0.0200**	0.0012	0.0016	604aa	KGI_S1_ANT01_95mDRAFT_100101672	Gammaproteobacteria bacterium (marine sediment metagenome, China: Bohai Sea)	NCF50312<br>68.28 % id<br>85 % cov
OPU57	0.0003	0.0004	**0.0055**	**0.0033**	457aa	TDF_OR_ARG05_123mDRAFT_10191591	*Halioglobus pacificus* (isolate, seawater)	WP_076001792<br>69.45 % id<br>99 % cov
OPU58	**0.0100**	**0.0086**	0.0000	0.0000	250aa	KGI_S1_ANT02_95mDRAFT_100840231	*Ilumatobacter coccineus* (isolate, soil)	WP_015439844<br>**55.86 % id**<br>88 % cov
OPU59	0.0001	0.0002	**0.0066**	**0.0061**	149aa	TDF_MC_ARG03_113mDRAFT_10636131	*Parahaliea mediterránea* (isolate, seawater at 10 m depth)	WP_116368122<br>65.71 % id<br>93 % cov
OPU63	0.0002	0.0005	**0.0144**	**0.0189**	336aa	TDF_OR_ARG05_123mDRAFT_10252072	Myxococcales bacterium SG8_38_1 (sediment metagenome, USA: White Oak Rive Estuary, North Carolina)	KPK52189<br>76.79 % id<br>100 % cov
OPU76	0.0000	0.0000	**0.0231**	**0.0171**	430aa	TDF_MC_ARG03_113mDRAFT_10031642	Gammaproteobacteria bacterium BRH_c0 (rock porewater metagenome, Switzerland: Mt Terri URL, St-Ursanne)	KJS07349<br>**36.34 % id**<br>100 % cov
OPU82	0.0000	0.0000	**0.0035**	**0.0026**	127aa	TDF_OR_ARG05_123mDRAFT_10784972	Gammaproteobacteria bacterium MnB_17 (bioreactor metagenome)	NJD30897<br>62.90 % id<br>97 % cov
OPU85	**0.0129**	**0.0160**	0.0000	0.0000	296aa	KGI_S1_ANT02_95mDRAFT_100798501	Candidatus Microthrix parvicella (uncultured, activated sludge)	WP_020378206<br>**54.12 % id**<br>65 % cov
OPU87	**0.0076**	**0.0131**	0.0000	0.0000	379aa	KGI_S2_ANT06_2345mDRAFT_10222182	Woeseiaceae bacterium (marine sediment metagenome, New Zealand: Hikurangi Margin)	NOR37717<br>**48.17 % id**<br>100 % cov
OPU88	0.0001	0.0002	**0.0091**	**0.0090**	320aa	TDF_OR_ARG05_123mDRAFT_10039371	Myxococcales bacterium (beach sand metagenome, Australia: Middle Park Beach, Port Phillip Bay)	NNE19414<br>91.88 % id<br>100 % cov
OPU96	**0.0031**	**0.0029**	0.0001	0.0002	139aa	KGI_S1_ANT01_95mDRAFT_102805091	Gammaproteobacteria bacterium (marine sediment metagenome, China: Bohai Sea)	NCF64297<br>76.81 % id<br>99 % cov

OPU99	0.0004	0.0009	**0.0111**	**0.0217**	123aa	TDF_OR_ARG05_123mDRAFT_10797242	Deltaproteobacteria bacterium (marine sediment metagenome, Mexico: Guaymas Basin, Gulf of California)	RLB44376 90.24 % id 100 % cov
OPU100	0.0001	0.0002	**0.0046**	**0.0041**	114aa	TDF_MC_ARG03_113mDRAFT_11318301	Gammaproteobacteria bacterium (marine sediment metagenome, Mexico: Guaymas Basin, Gulf of California)	RLA54766 71.43 % id 98 % cov
OPU104	**0.0046**	**0.0045**	0.0000	0.0000	164aa	KGI_S2_ANT04_2345mDRAFT_10425512	*Psychrobacter* sp. N25K4-3-2 (isolate, seawater, Arctic Ocean)	WP_194620736 99.29 % id 85% cov
OPU105	0.0001	0.0002	**0.0035**	**0.0025**	149aa	TDF_MC_ARG03_113mDRAFT_10425282	Gammaproteobacteria bacterium (marine sediment metagenome, New Zealand: Hikurangi Margin)	NOR40796 **55.41 % id** 99 % cov
OPU111	**0.0037**	**0.0017**	0.0001	0.0001	290aa	KGI_S1_ANT02_95mDRAFT_100822201	Flavobacteriaceae bacterium (beach sand metagenome, Australia: Middle Park Beach, Port Phillip Bay)	NNC70101 69.90 % id 99 % cov
OPU120	**0.0024**	**0.0023**	0.0001	0.0002	136aa	KGI_S1_ANT01_95mDRAFT_102903211	*Spongiibacter* sp. KMU-166 (isolate, coastal seawater, Korea)	WP_168448680 **59.56 % id** 100 % cov
OPU125	**0.0032**	**0.0034**	0.0000	0.0000	84aa	KGI_S1_ANT01_95mDRAFT_30684658961	Actinobacteria bacterium (freshwater metagenome, Czech Republic: Rimov Reservoir)	MTA11731 **58.54 % id** 98 % cov
OPU129	**0.0172**	**0.0167**	0.0002	0.0005	220aa	KGI_S1_ANT01_95mDRAFT_101199441	Acidimicrobiales bacterium (beach sand metagenome, Australia: Melbourne, Middle Park Beach)	RZV41408 63.93 % id 99 % cov
OPU135	0.0003	0.0004	**0.0049**	**0.0036**	129aa	TDF_MC_ARG03_113mDRAFT_10922691	Spongiibacteraceae bacterium (marine metagenome, Indian Ocean)	MBN50440 74.42 % id 100 % cov
OPU136	0.0001	0.0001	**0.0022**	**0.0017**	110aa	TDF_OR_ARG05_123mDRAFT_11056121	*Halioglobus* sp. HI00S01 (isolate, Pacific Ocean)	WP_066051514 82.73 % id 100 % cov
OPU144	**0.0182**	**0.0265**	0.0001	0.0001	130aa	KGI_S1_ANT01_95mDRAFT_103124491	Armatimonadetes bacterium (metagenome marine ANAMMOX bioreactor)	KAA3640675 72.31 % id 100 % cov
OPU158	**0.0047**	**0.0053**	0.0001	0.0002	143aa	KGI_S2_ANT05_2345mDRAFT_11076751	Xanthomonadales bacterium (beach sand metagenome, Australia: Middle Park Beach, Port Phillip Bay site C)	NNJ65315 65.96 % cov 98 % cov

OPU163	0.0000	0.0000	**0.0058**	**0.0079**	173aa	TDF_OR_ARG05_123mDRAFT_10688762	Myxococcales bacterium SG8_38 (sediment metagenome, SA: White Oak River Estuary, North Carolina)	KPK17353<br>68.29 % id<br>94 % cov
OPU164	0.0001	0.0003	**0.0032**	**0.0031**	142aa	TDF_OR_ARG05_123mDRAFT_10508652	Deltaproteobacteria bacterium (sediment metagenome, USA: North Dakota, Cottonwood Lake Study Area)	RPJ82527<br>76.76 % id<br>100 % cov
OPU165	0.0000	0.0000	**0.0038**	**0.0055**	107aa	TDF_OR_ARG05_123mDRAFT_12872821	Desulfobacteraceae bacterium (cold seep metagenome, Atlantic Ocean: Gulf of Cadiz)	MBC2736927<br>88.79 % id<br>100 % cov
OPU167	0.0000	0.0001	**0.0043**	**0.0036**	91aa	TDF_MC_ARG02_113mDRAFT_10686741	*Zhongshania antarctica* (isolate, coastal fast ice)	WP_184461034<br>73.63 % id<br>100 % cov
OPU172	**0.0016**	**0.0012**	0.0000	0.0000	53aa	KGI_S1_ANT02_95mDRAFT_3357962781	Proteobacteria bacterium (cold seep metagenome, Pacific Ocean: South China Sea)	MBE9548527<br>80.39 % cov<br>96 % cov
OPU175	0.0001	0.0002	**0.0120**	**0.0229**	161aa	TDF_MC_ARG03_113mDRAFT_10538481	Gammaproteobacteria bacterium (marine sediment metagenome, China: Bohai Sea)	NCF64297<br>**47.80 % id**<br>98 % cov
OPU177	0.0004	0.0009	**0.0041**	**0.0051**	131aa	TDF_MC_ARG03_113mDRAFT_10876041	Proteobacteria bacterium (cold seep metagenome, Pacific Ocean: South China Sea)	MBE9540181<br>**57.14 % id**<br>94 % cov
OPU189	0.0000	0.0000	**0.0021**	**0.0013**	99aa	TDF_OR_ARG04_123mDRAFT_11327881	Deltaproteobacteria bacterium (soil metagenome, USA: Angelo Coast Range Reserve, CA)	TMB22854<br>71.72 % id<br>100 % cov
OPU200	0.0000	0.0001	**0.0014**	**0.0007**	82aa	TDF_OR_ARG06_123mDRAFT_3003194501	Myxococcales bacterium SG8_38 (sediment metagenome, USA: White Oak Rive Estuary, North Carolina)	KPK15390<br>**53.75 % id**<br>97 % cov
OPU206	**0.0020**	**0.0018**	0.0000	0.0001	110aa	KGI_S1_ANT02_95mDRAFT_103785561	Armatimonadetes bacterium (metagenome marine ANAMMOX bioreactor)	KAA3640675<br>86.36 % id<br>100 % cov
OPU212	**0.0012**	**0.0004**	0.0000	0.0000	79aa	KGI_S1_ANT01_95mDRAFT_30284900961	Pyrinomonadaceae bacterium (beach sand metagenome, Australia: Middle Park Beach, Port Phillip Bay)	NNE98286<br>81.01 % id<br>100 % cov
OPU241	**0.0047**	**0.0046**	0.0000	0.0000	135aa	KGI_S1_ANT03_95mDRAFT_10732291	*Halioglobus* sp. Bin NAT121 (marine water sample metagenome, South Atlantic Ocean)	MAT91988<br>62.22 % id<br>100 % cov

OPU243	0.0000	0.0000	**0.0043**	**0.0077**	110aa	TDF_MC_ARG03_113mDRAFT_10561801	Myxococcales bacterium (marine metagenome, Pacific Ocean: North Pacific Gyre)	NRA00267 **46.36 % id** 100 % cov
OPU252	**0.0096**	**0.0128**	0.0001	0.0002	107aa	KGI_S1_ANT01_95mDRAFT_100810951	*Parasphingopyxis lamellibrachiae* (isolate, Trophosome of annelid worm *Lamellibrachia satsuma*)	RED16357 97.20 % id 100 % cov
OPU272	**0.0017**	**0.0014**	0.0000	0.0000	79aa	KGI_S1_ANT01_95mDRAFT_30466941221	Actinobacteria bacterium (soil metagenome, temperate grassland biome)	TML91183 **58.33 % id** 91 % cov
OPU278	**0.0029**	**0.0031**	0.0000	0.0000	197aa	KGI_S2_ANT05_2345mDRAFT_10628451	*Parahaliea aestuarii* (isolate, sediment of the Asan Bay estuary)	WP_148067434 **44.21% id** 96 % cov
OPU302	0.0001	0.0001	**0.0152**	**0.0067**	471aa	TDF_MC_ARG01_113mDRAFT_10004642	*Haliea* sp. Bin 47 (marine sediment metagenome, China: Bohai Sea)	NCF19241 **37.71 % id** 99 % cov
OPU306	**0.0016**	**0.0015**	0.0000	0.0000	110aa	KGI_S1_ANT01_95mDRAFT_104319971	Proteobacteria bacterium (cold seep metagenome, Pacific Ocean: South China Sea)	MBE9548527 79.09 % id 100 % cov
OPU336	**0.0013**	**0.0011**	0.0000	0.0000	84aa	KGI_S1_ANT01_95mDRAFT_30602381861	*Parahaliea aestuarii* (isolate, sediment of the Asan Bay estuary)	WP_148068479 70.24 % id 100% cov
OPU358	**0.0022**	**0.0024**	0.0000	0.0000	102aa	KGI_S1_ANT01_95mDRAFT_102636262	bacterium BMS3Abin02 (sub-seafloor sulfide deposits metagenome)	GBD84161 66.67 % id 100 % cov
OPU359	**0.0056**	**0.0116**	0.0000	0.0000	91aa	KGI_S1_ANT01_95mDRAFT_30410616411	Proteobacteria bacterium (cold seep metagenome, Pacific Ocean: South China Sea)	MBE9548527 75.82 % id 100 cov
OPU413	0.0000	0.0000	**0.0164**	**0.0142**	464aa	TDF_OR_ARG05_123mDRAFT_10132603	*Haliea* sp. Bin 47 (marine sediment metagenome, China: Bohai Sea)	NCF19241 **37.93 % id** 100 % cov
OPU415	**0.0083**	**0.0104**	0.0000	0.0000	331aa	KGI_S1_ANT01_95mDRAFT_100621161	Gammaproteobacteria bacterium 42_54_T18 (metagenome of sea water enriched with oil for 18 days, USA: Gulf of Mexico)	OUR89724 **54.19 % id** 100 % cov
OPU454	0.0000	0.0000	**0.0025**	**0.0017**	177aa	TDF_MC_ARG03_113mDRAFT_10434601	Gammaproteobacteria bacterium BRH_c0 (rock porewater metagenome, Switzerland: Mt Terri URL, St-Ursanne)	KJS07349 **34.66 % id** 98 % cov

Bold fonts indicate significantly higher relative abundance values.
