# Supplementary material for: High wax ester and triacylglycerol biosynthesis potential in coastal sediments of Antarctic and Subantarctic environments
Source: PLoS One. 2023 Jul 17;18(7):e0288509. doi: 10.1371/journal.pone.0288509 (PMC10351704; doi:10.1371/journal.pone.0288509)
Supplement: S6 Table — (PDF) [file pone.0288509.s006.pdf]

S6 Table. WS/DGAT homolog sequences identified in OR07 metagenome.

WS/DGAT homolog sequence				Scaffold		
**Sequence name**	**Sequence length**	**Taxonomic binning**	**First match Blastp against nr NCBI database**	**Scaffold name**	**Scaffold length (bp)**	**Scaffold taxonomic assignment**
gene_347862|476_aa|+|14232|15662_S1-350763	476	Acidobacteriota	RMH16941, 61.75% id, 100% cov, Acidobacteria bacterium bin J023 (Acidobaceria phylum), hot springs metagenome	>S1_scaffold-350763	35,597	Acidobacteriota
gene_25122|479_aa|-|18337|19776_S2-1349	479	Acidobacteriota	NNE98286, 77.87% id, 100% cov, Pyrinomonadaceae bacterium SS_bin_32 (Acidobacteria; Blastocatellia), beach sand metagenome	>S2_scaffold-1349	23,097	Bacteria
gene_265015|480_aa|+|1617|3059_S1-46305	480	Acidobacteriota	NNE98286, 79.66% id, 98% cov, Pyrinomonadaceae bacterium SS_bin_32 (Acidobacteria; Blastocatellia), beach sand metagenome	>S1_scaffold-46305	3,202	Bacteria
gene_283693|496_aa|+|15782|17272_S2-500566	496	Acidobacteriota	NNE98286, 78.22% id, 95% cov, Pyrinomonadaceae bacterium SS_bin_32 (Acidobacteria; Blastocatellia), beach sand metagenome	>S2_scaffold-500566	34,024	Bacteria
P_gene_260578|479_aa|+|1|1437_S2-139233	479	Acidobacteriota	NNE98286, 78.29% id, 100% cov, Pyrinomonadaceae bacterium SS_bin_32 (Acidobacteria; Blastocatellia), beach sand metagenome	>S2_scaffold-139233	1,439	Bacteria
gene_269914|593_aa|-|3239|5020_S1-50732	593	Bacteria	PYT05365, 43.13% id, 79% cov, Acidobacteria bacterium bin gp4 AA12 (Acidobacteria phylum), soil metagenome	>S1_scaffold-50732	5,880	Bacteria
gene_255112|480_aa|+|289|1731_S1-38999	480	Bacteria	RMH16941, 52.46% id, 100% cov, Acidobacteria bacterium bin J023 (Acidobacteria phylum), hot springs metagenome	>S1_scaffold-38999	2,029	Bacteria
gene_94858|345_aa|-|2132|3169_S1-5992	345	Bacteria	MBT8164997, 70.35% id, 99% cov, Acidimicrobiia bacterium SS_bin_3 (Actinobacteria; Acidimicrobiia), beach sand metagenome	>S1_scaffold-5992	35,086	Bacteria
gene_288172|472_aa|-|13078|14496_S2 -571919	472	Bacteria	REK19812, 80.73% id, 98% cov, Actinobacteria bacterium bin MA_ANB_1 (Actinobacteria phylum), biofilter metagenome	>S2_scaffold-571919	34,112	Bacteria
gene_17561|476_aa|+|23376|24806_S2-756	476	Bacteria	NQY57642, 59.70% id, 96% cov, lumatobacteraceae bacterium bin DT_55 (Actinobacteria; Acidimicrobiia), marine metagenome	>S2_scaffold-756	26,514	Bacteria
P_gene_206873|439_aa|+|208|1524_ S2-56901	439	Bacteria	REK19812, 65.83% id, 100% cov, Actinobacteria bacterium bin MA_ANB_1 (Actinobacteria phylum), biofilter metagenome	>S2_scaffold-56901	1,526	Bacteria
P_gene_339809|530_aa|+|1088|2677_S1-257946	530	Bacteria	NNF53608, 91.29% id, 99% cov, Acidimicrobiales bacterium SS_bin_9 (Actinobacteria; Acidimicrobiia), beach sand metagenome	>S1_scaffold-257946	2,677	Bacteria
gene_90115|456_aa|+|16910|18280_S1-5812	456	Bacteria	MBL8778193, 45.05%, 97%, Acidimicrobiales bacterium new MAG-69 (Actinobacteria; Acidimicrobiia), bioreactor metagenome	>S1_scaffold-5812	36,791	Actinomycetota phylum
gene_117505|476_aa|-|10877|12307_S2-6630	476	Bacteria	NQY57642, 59.27% id, 96% cov, Ilumatobacteraceae bacterium (Actinobacteria; Acidimicrobiia), marine metagenome	>S2_scaffold-6630	35,928	Actinomycetota phylum
gene_147862|493_aa|+|1695|3176_S2-12741	493	Bacteria	MSO38604, 49.69% id, 97% cov, Acidimicrobiia bacterium bin Baikal-deep-G23 (Actinobacteria; Acidimicrobiia), freshwater metagenome	>S2_scaffold-12741	4,841	Actinomycetota phylum

gene_295736|465_aa|-|30071|31468_S2-612579	465	Bacteria	MBA2326156, 43.01% id, 98% cov, Actinobacteria bacterium MGR_bin273 (Actinobacteria phylum), soil metagenome	>S2_scaffold-612579	34,919	Actinomycetes class
gene_25382|472_aa|-|4168|5586_S1-1032	472	Bacteria	REK19812, 80.73% id, 98% cov, Actinobacteria bacterium bin MA_ANB_1 (Actinobacteria phylum), RO biofilter metagenome	>S1_scaffold-1032	11,273	Actinomycetes class
gene_285621|457_aa|+|14281|15654_S2-535623	457	Bacteria	HAS12345, 66.37% id, 99% cov, Acidimicrobiaceae bacterium bin UBA9382 (Actinobacteria; Acidimicrobiia), marine metagenome	>S2_scaffold-535623	37,162	Actinomycetes class
P_gene_301916|373_aa|+|293|1411_S1-89338	373	Bacteria	MBC8340401, 40.06% id, 93% cov, Proteobacteria bacterium bin NIOZ-UU67, marine metagenome	>S1_scaffold-89338	1,413	Actinomycetota phylum
P_gene_212465|283_aa|-|3|851_S1-19187	283	Bacteria	MBM4243484, 41.58% id, 95% cov, Deltaproteobacteria bacterium K_Offshore_80m_m2_211, freshwater metagenome	>S1_scaffold-19187	4,954	Actinomycetota phylum
gene_218987|464_aa|+|2516|3910_S1-21622	464	Actinomycetota phylum	HAS12345, 66.85% id, 99% cov, Acidimicrobiaceae bacterium bin UBA9382 (Actinobacteria; Acidimicrobiia), marine metagenome	>S1_scaffold-21622	40,795	Actinomycetota phylum
gene_165527|514_aa|+|29090|30634_S2-20677	514	Actinomycetota phylum	MBK5288739, 44.23% id, 98% cov, Acidimicrobiia bacterium 3_4_m_bin14 (Actinobacteria; Acidimicrobiia), permafrost sediment metagenome	>S2_scaffold-20677	31,551	Actinomycetota phylum
gene_144528|458_aa|+|16142|17518_S1-8331	458	Actinomycetota phylum	MBW3663268, 58.13% id, 99% cov, Actinobacteria bacterium 0813_bin37, desert regolith metagenome	>S1_scaffold-8331	30,627	Actinomycetota phylum
gene_152681|554_aa|-|15092|16756_S2-14512	554	Actinomycetota phylum	MAT05148, 73.62% id, 88% cov, Acidimicrobiaceae bacterium bin NAT239 (Actinobacteria; Acidimicrobiia), marine metagenome	>S2_scaffold-14512	25,560	Actinomycetota phylum
gene_229106|465_aa|+|893|2290_S1-25453	465	Actinomycetota phylum	MBV8959391, 60.09% id, 99% cov, Actinobacteria bacterium CP_BM_ER_R9_11, rhizosphere metagenome	>S1_scaffold-25453	19,239	Actinomycetota phylum
gene_40848|568_aa|-|1418|3124_S1-2056	568	Actinomycetota phylum	WP_219941651, 77.59% id, 812% cov, Iamia sp. SCSIO 61187 (Actinobacteria; Acidimicrobiia), isolate	>S1_scaffold-2056	18,429	Actinomycetota phylum
gene_282186|576_aa|-|2346|4076_S2-462764	576	Actinomycetota phylum	NNF53608, 83.78% id, 99% cov, Acidimicrobiales bacterium SS_bin_9 (Actinobacteria; Acidimicrobiia), beach sand metagenome	>S2_scaffold-462764	5,243	Actinomycetota phylum
gene_301529|502_aa|-|18|1526_S1-88733	502	Actinomycetota phylum	MSO86604, 65.34% id, 94% cov, Acidimicrobiia bacterium bin Baikal-deep-G25 (Actinobacteria; Acidimicrobiia), freshwater metagenome	>S1_scaffold-88733	1,808	Actinomycetota phylum
gene_374107|465_aa|-|24405|25802_S1-504327	465	Actinomycetota phylum	NQY56454, 45.68% id, 99% cov, Ilumatobacteraceae bacterium bin DT_55 (Actinobacteria; Acidimicrobiia), marine metagenome	>S1_scaffold-504327	28,736	Bacteria
gene_367250|576_aa|-|9139|10869_S1-494581	576	Actinomycetota phylum	NNF53608, 83.78% id, 99% cov, Acidimicrobiales bacterium SS_bin_9 (Actinobacteria; Acidimicrobiia), beach sand metagenome	>S1_scaffold-494581	12,368	Bacteria
P_gene_102545|444_aa|-|2|1333_S2-5353	444	Actinomycetota phylum	MBL8776107, 56.47% id, 99% cov, Acidimicrobiales bacterium new MAG-69 (Actinobacteria; Acidimicrobiia), bioreactor metagenome	>S2_scaffold-5353	3,561	Actinomycetota phylum
P_gene_334223|360_aa|-|1|1080_S1-201397	360	Actinomycetota phylum	HAS12345, 64.25% id, 99% cov, Acidimicrobiaceae bacterium bin UBA9382 (Actinobacteria; Acidimicrobiia), marine metagenome	>S1_scaffold-201397	1,419	Actinomycetota phylum

P_gene_211649|350_aa|-|690|1742_S2-62153	350	Actinomycetota phylum	NNC80367, 46.74% id, 97% cov, Acidimicrobiales bacterium SS_bin_103 (Actinobacteria; Acidimicrobiia), beach sand metagenome	>S2_scaffold-62153	1,744	Actinomycetota phylum
P_gene_268510|323_aa|-|1|969_S1-49302	323	Actinomycetota phylum	MSO38604, 49.85% id, 100% cov, Acidimicrobiia bacterium bin Baikal-deep-G23 (Actinobacteria; Acidimicrobiia), freshwater metagenome	>S1_scaffold-49302	3,385	Bacteria
P_gene_318645|182_aa|+|1030|1575_S1-126867	182	Actinomycetota phylum	MSO86549, 63.89% id, 79% cov, Acidimicrobiia bacterium CP_BM_RX_R9_22 (Actinobacteria; Acidimicrobiia), rhizosphere metagenome	>S1_scaffold-126867	1,577	Actinomycetota phylum
P_gene_350953|180_aa|+|472|1011_S1-393327	180	Actinomycetota phylum	NNE11106, 84.44% id, 100% cov, Ilumatobacter sp. SS_bin_49 (Actinobacteria; Acidimicrobiia), beach sand metagenome	>S1_scaffold-393327	1,011	Actinomycetota phylum
P_gene_250078|159_aa|-|3|479_S2-110208	159	Actinomycetota phylum	MBT5753879, 91.10% id, 91% cov, Acidimicrobiaceae bacterium SI072_bin49 (Actinobacteria; Acidimicrobiia), hypoxic seawater metagenome	>S2_scaffold-110208	1,332	Actinomycetota phylum
gene_173269|582_aa|+|18267|20015_S2-25494	582	Actinomycetota phylum	NNE75090, 60.04% id, 82% cov, Acidimicrobiales bacterium SS_bin_33 (Actinobacteria; Acidimicrobiia), beach sand metagenome	>S2_scaffold-25494	36,533	Actinomycetota phylum
gene_173271|513_aa|+|21607|23148_S2-25494	513	Actinomycetota phylum	MBT6444723, 69.15%, 96%, Acidimicrobiaceae bacterium SI073_bin64 (Actinobacteria; Acidimicrobiia), hypoxic seawater metagenome			
P_gene_15091|354_aa|-|1|1062_S1-572	354	Actinomycetes class	WP_152177379, 70.62% id, 100% cov, *Tetrasphaera* sp. F2B08 (Actinobacteria; Micrococcales), marine sediment isolate	>S1_scaffold-572	39,949	Actinomycetota phylum
gene_15092|469_aa|-|1072|2481_S1-572	469	Actinomycetota phylum	NQY57642, 70.72% id, 98% cov, Ilumatobacteraceae bacterium (Actinobacteria; Acidimicrobiia), marine metagenome			
gene_36942|496_aa|-|9478|10968_S1-1744	496	Actinomycetes class	MBT6444723, 68.21% id, 99% cov, Acidimicrobiaceae bacterium SI073_bin64 (Actinobacteria; Acidimicrobiia), marine metagenome	>S1_scaffold-1744	35,820	Actinomycetota phylum
gene_36944|584_aa|-|12863|14614_S1-1744	584	Actinomycetota phylum	NND75404, 61.80% id, 82% cov, Ilumatobacter sp. SS_bin_52 (Actinobacteria; Acidimicrobiia), beach sand metagenome			
gene_113317|443_aa|-|10876|12207_S1-6720	443	Acidimicrobiia class	RZV41408, 64.81% id, 93% cov, Acidimicrobiales bacterium MetaBAT.bin.19 (Actinobacteria; Acidimicrobiia), beach sand metagenome	>S1_scaffold-6720	23,289	Actinomycetota phylum
gene_113323|486_aa|-|16493|17953_S1-6720	486	Actinomycetota phylum	NNC79469, 52.24% id, 95% cov, Acidimicrobiales bacterium SS_bin_103 (Actinobacteria; Acidimicrobiia), beach sand metagenome			
gene_279162|518_aa|+|6323|7879_S2-371811	518	Actinomycetota phylum	HAY66693, 61.52% id, 96% cov, Acidimicrobiaceae bacterium bin UBA10347 (Actinobacteria; Acidimicrobiia), marine metagenome	>S2_scaffold-371811	13,340	Actinomycetota phylum
gene_279163|496_aa|+|7876|9366_S2-371811	496	Actinomycetota phylum	MBT5850753, 55.03% id, 94% cov, Acidimicrobiaceae bacterium SI072_bin141 (Actinobacteria; Acidimicrobiia), marine metagenome			
gene_166297|456_aa|-|3091|4461_S2-21143	456	Acidimicrobiia class	MBK9972909, 53.49% id, 99% cov, Acidimicrobiaceae bacterium bin Skiv_18-Q3-R9-52_BAT3C.106, activated sludge metagenome	>S2_scaffold-21143	13,166	Actinobacteria class

gene_366377|567_aa|+|6617|8320_S1-492408	567	Acidimicrobiia class	MAT05148, 72.20% id, 91% cov, Acidimicrobiaceae bacterium bin NAT239 (Actinobacteria; Acidimicrobiia), seawater metagenome	>S1_scaffold-492408	40,186	Actinomycetota phylum
gene_147346|539_aa|+|2902|4521_S2_scaffold-12626	539	Acidimicrobiia class	MBT5276300, 84.02% id, 90% cov, Ilumatobacter sp. SI074_bin20 (Actinobacteria; Acidimicrobiia), hypoxic seawater metagenome	>S2_scaffold-12626	39,873	Actinomycetota phylum
gene_373098|586_aa|-|6847|8607_S1-503448	586	Acidimicrobiia class	MBT4984432, 70.91% id, 90% cov, Ilumatobacter sp. SI075_bin120 (Actinobacteria; Acidimicrobiia), hypoxic seawater metagenome	>S1_scaffold-503448	38,556	Actinomycetota phylum
gene_192106|608_aa|-|22385|24211_S2-42063	608	Acidimicrobiia class	WP_083914852, 80.70% id, 89% cov, *Ilumatobacter nonamiensis* (Actinobacteria; Acidimicrobiia), isolate seashore sand	>S2_scaffold-42063	38,074	Actinomycetota phylum
gene_344934|593_aa|+|31732|33513_S1-313442	593	Acidimicrobiia class	MBT4984432, 77.63% id, 88% cov, Ilumatobacter sp. SI075_bin120 (Actinobacteria; Acidimicrobiia), hypoxic seawater metagenome	>S1_scaffold-313442	35,395	Actinomycetota phylum
gene_378436|470_aa|-|6112|7524_S1-506487	~~470~~467	Acidimicrobiia class	WP_155852215, 86.35% id, 99% cov, Candidatus Microthrix parvicella (Actinobacteria; Acidimicrobiia), activated sludge	>S1_scaffold-506487	35,301	Actinomycetota phylum
gene_55560|458_aa|-|10095|11471_S1-3683	458	Acidimicrobiia class	TVR24770, 72.65% id, 99% cov, Ilumatobacter sp. GEM2.Bin45 (Actinobacteria; Acidimicrobiia), soda lake metagenome	>S1_scaffold-3683	35,221	Actinomycetota phylum
gene_214160|557_aa|+|6763|8436_S1-19775	557	Acidimicrobiia class	MBT5276300, 80.82% id, 87% cov, Ilumatobacter sp. SI074_bin20 (Actinobacteria; Acidimicrobiia), hypoxic seawater metagenome	>S1_scaffold-19775	34,683	Actinomycetota phylum
gene_377582|537_aa|-|10221|11834_S1-506449	537	Acidimicrobiia class	MBT6444724, 86.61% id, 91% covAcidimicrobiaceae bacterium SI073_bin64 (Actinobacteria; Acidimicrobiia), hypoxic seawater metagenome	>S1_scaffold-506449	34,395	Actinomycetota phylum
gene_11531|458_aa|-|900|2276_S2-445	458	Acidimicrobiia class	MAT04250, 72.71% id, 99% cov, Acidimicrobiaceae bacterium bin NAT239 (Actinobacteria; Acidimicrobiia), marine metagenome	>S2_scaffold-445	33,040	Actinomycetota phylum
gene_20441|575_aa|+|9273|11000_S2-943	575	Acidimicrobiia class	NND74478, 72.43% id, 90% cov, Ilumatobacter sp. SS_bin_52 (Actinobacteria; Acidimicrobiia), beach sand metagenome	>S2_scaffold-943	32,262	Actinomycetota phylum
gene_170707|475_aa|+|12337|13764_S1-10544	475	Acidimicrobiia class	MBK6969467, 75.52% id, 100% cov, Candidatus Microthrix sp. Bjer_18-Q3-R1-45_BATAC.226 (Actinobacteria; Acidimicrobiia), activated sludge metagenome	>S1_scaffold-10544	31,626	Actinomycetota phylum
gene_2794|509_aa|+|20025|21554_S2-93	509	Acidimicrobiia class	RLE26694, 68.17% id, 99% cov, Actinobacteria bacterium bin B28_G4 (Actinobacteria phylum), marine sediment metagenome	>S2_scaffold-93	27,288	Actinomycetota phylum
gene_25272|551_aa|+|9746|11401_S2-1364	551	Acidimicrobiia class	MBT5554502, 80.25% id, 88% cov, Ilumatobacter sp. SI073_bin179 (Actinobacteria; Acidimicrobiia), marine metagenome	>S2_scaffold-1364	18,225	Actinomycetota phylum
gene_219394|456_aa|-|10020|11390_S1-21734	456	Acidimicrobiia class	MBK9972909, 53.49% id, 99% cov, Acidimicrobiaceae bacterium bin Skiv_18-Q3-R9-52_BAT3C.106, activated sludge metagenome	>S1_scaffold-21734	12,645	Actinomycetota phylum
gene_45726|607_aa|-|3236|5059_S1-2500	607	Acidimicrobiia class	MBT4984432, 79.25% id, 87% cov, Ilumatobacter sp. Bin SI075_bin120 (Actinobacteria; Acidimicrobiia), hypoxic seawater metagenome	>S1_scaffold-2500	10,683	Actinomycetota phylum

gene_356107|472_aa|+|5883|7301_S1-446813	472	Acidimicrobiia class	WP_158412221, 74.68% id, 99% cov, *Ilumatobacter nonamiensis* (Actinobacteria; Acidimicrobiia), isolate	>S1_scaffold-446813	9,091	Actinomycetota phylum
gene_515|559_aa|+|30569|32245_S2-16	559	Acidimicrobiia class	MBT4984432, 80.83% id, 94% cov, Ilumatobacter sp. SI075_bin120 (Actinobacteria; Acidimicrobiia), hypoxic seawater metagenome	>S2_scaffold-16	33,677	Actinomycetota phylum
gene_242978|387_aa|-|6094|7257_S1-31820	387	Acidimicrobiia class	MBT5276300, 79.07% id, 88% cov, Ilumatobacter sp. SI074_bin20 (Actinobacteria; Acidimicrobiia), marine metagenome	>S1_scaffold-31820	7,324	Actinomycetota phylum
gene_188610|450_aa|+|13060|14412_S1-13028	450	Acidimicrobiia class	MBT8240663, 78.97% id, 99% cov, Acidimicrobiales bacterium HDS-22 (Actinobacteria; Acidimicrobiia), beach sand metagenome	>S1_scaffold-13028	41,843	Bacteria
P_gene_204784|365_aa|-|3|1097_S2-54757	365	Actinomycetes class	NCF16877, 39.68% id, 99% cov, Haliea sp. bin.47 (Gammaproteobacteria; Cellvibrionales), marine sediment metagenome	>S2_scaffold-54757	1,099	Bacteria
P_gene_343055|338_aa|-|3|1016_S1-292897	338	Acidimicrobiia class	MBT8240663, 79.23% id, 99% cov, Acidimicrobiia bacterium HDS-22 (Actinobacteria; Acidimicrobiia), beach sand metagenome	>S1_scaffold-292897	1,016	Bacteria
P_gene_54038|276_aa|+|1|828_S1-3488	276	Acidimicrobiia class	MBT4984432, 82.61% id, 100% cov, Ilumatobacter sp. SI075_bin120 (Actinobacteria; Acidimicrobiia), hypoxic seawater metagenome	>S1_scaffold-3488	1,451	Actinomycetota phylum
P_gene_294063|237_aa|-|1|711_S1-76997	237	Acidimicrobiia class	MBT8240663, 76.27% id, 99% cov, Acidimicrobiia bacterium HDS-22 (Actinobacteria; Acidimicrobiia), beach sand metagenome	>S1_scaffold-76997	1,758	Actinomycetota phylum
P_gene_176360|189_aa|-|3|569_S2-27818	189	Acidimicrobiia class	MBC49189, 91.01% id, 100% cov, Ilumatobacter sp. SI075_bin120 (Actinobacteria; Acidimicrobiia), marine metagenome	>S2_scaffold-27818	8,926	Actinomycetota phylum
P_gene_189124|154_aa|+|3761|4222_S2-38922	154	Acidimicrobiia class	WP_083914852, 83.77% id, 100% cov, *Ilumatobacter nonamiensis* (Actinobacteria; Acidimicrobiia), isolate	>S2_scaffold-38922	4,222	Actinomycetota phylum
gene_181105|481_aa|-|32895|34340_S2-13156	481	Actinomycetes class	WP_126333966, 59.03% id, 98% cov, *Mycolicibacterium chitae* (Actinobacteria; Corynebacteriales), isolate	>S1_scaffold-11827	40,919	Actinobacteria class
gene_149256|467_aa|-|21836|23239_S1-1838	467	Actinomycetes class	WP_091193045, 93.68% id, 98% cov, *Nocardioides alpinus* (Actinobacteria; Propionibacteriales), isolate alpine glacier cryoconite	>S2_scaffold-13156	33,650	Actinobacteria class
gene_38160|473_aa|+|7057|8478_S1-1838	473	Actinomycetes class	RLE22872, 63.50% id, 97% cov, Actinobacteria bacterium bin B28_G4 (Actinobacteria phylum), marine sediment metagenome	>S1_scaffold-1838	22,345	Actinobacteria class
gene_117001|955_aa|-|6334|9201_S1-6880	955	Actinomycetes class	NNE72226, 37.36%, 98%, Acidimicrobiales bacterium SS_bin_33 (Actinobacteria; Acidimicrobiia), beach sand metagenome	>S1_scaffold-6880	41,774	Actinomycetota phylum
gene_34225|464_aa|-|19038|20432_S1-1556	464	Actinomycetes class	WP_216998409, 79.07% id, 97% cov, *Aquihabitans* sp. G128 (Actinobacteria; Acidimicrobiia), isolate	>S1_scaffold-1556	31,869	Actinomycetota phylum
gene_226601|486_aa|+|16130|17590_S1-24639	486	Actinomycetes class	MBN4047461, 55.31% id, 99% cov, Acidimicrobiaceae bacterium AH-315-P05 (Actinobacteria; Acidimicrobiia), Atlantic Ocean single cell	>S1_scaffold-24639	31,626	Actinomycetota phylum
gene_364766|479_aa|-|28697|30136_S1-488279	479	Actinomycetes class	TMM17552, 45.55% id, 97% cov, Actinobacteria bacterium bin AC_9 (Actinobacteria phylum), soil metagenome	>S1_scaffold-488279	31,456	Actinomycetota phylum

gene_268271|345_aa|-|27690|28727_S1-49058	345	Actinomycetes class	NIR38689, 74.78% id, 100% cov, Actinobacteria bacterium bin KS3-K058 (Actinobacteria phylum), marine sediment metagenome	>S1_scaffold-49058	28,757	Actinomycetota phylum
gene_127014|465_aa|+|14537|15934_S1-7310	465	Actinomycetes class	WP_118396048, 84.98% id, 100% cov, *Aeromicrobium* sp. A1-2 (Actinobacteria; Propionibacteriales), isolate Southern Ocean	>S1_scaffold-7310	26,525	Actinomycetota phylum
gene_22831|473_aa|-|221|1642_S2-1134	473	Actinomycetes class	RLE22872, 63.50% id, 97% cov, Actinobacteria bacterium bin B28_G4 (Actinobacteria phylum), marine sediment metagenome	>S2_scaffold-1134	25,871	Actinomycetota phylum
gene_268611|405_aa|-|14002|15219_S2-184116	405	Actinomycetes class	MBG7603314, 64.99% id, 97% cov, Actinobacteria bacterium bin Sva-07 (Actinobacteria phylum), marine sediment metagenome	>S2_scaffold-184116	25,706	Actinomycetota phylum
gene_230413|483_aa|+|14616|16067_S1-25986	483	Actinomycetes class	WP_152177379, 72.52% id, 97% cov, *Tetrasphaera* sp. F2B08 (Actinobacteria; Micrococcales), isolate	>S1_scaffold-25986	19,775	Actinomycetota phylum
gene_28143|472_aa|-|5693|7111_S2-1718	472	Actinomycetes class	WP_141787473, 64.33% id, 99% cov, *Oryzihumus leptocrescens* (Actinobacteria; Micrococcales), isolate soil sample	>S2_scaffold-1718	8,931	Actinomycetota phylum
gene_207262|447_aa|-|5692|7035_S2-57419	447	Actinomycetes class	HBX79251, 64.80% id, 99% cov, Acidimicrobiaceae bacterium bin UBA11034 (Actinobacteria; Acidimicrobiia), wastewater metagenome	>S2_scaffold-57419	36,104	Bacteria
P_gene_117651|233_aa|-|1|699_S2-6654	233	Actinomycetes class	TML42584, 78.40% id, 91% cov, Actinobacteria bacterium bin AC_35 (Actinobacteria phylum), soil metagenome	>S2_scaffold-6654	3,433	Actinomycetota phylum
P_gene_263831|415_aa|-|2|1246_S1-45393	415	Actinomycetes class	RLE22872, 62.53% id, 100% cov, Actinobacteria bacterium bin B28_G4 (Actinobacteria phylum), marine sediment metagenome	>S1_scaffold-45393	1,921	Actinomycetota phylum
P_gene_293806|458_aa|-|1|1374_S1-76583	458	Actinomycetes class	MBK6312425, 51.99% id, 98% cov, Candidatus Microthrix sp. AalE_18-Q3-R2-46_BAT3C.202 (Actinobacteria; Acidimicrobiia), activated sludge metagenome	>S1_scaffold-76583	1,853	Actinomycetota phylum
P_gene_243543|90_aa|-|1|270_S2-98520	90	Actinomycetes class	TML91691, 48.84% id, 95% cov, Actinobacteria bacterium bin AC_14 (Actinobacteria phylum), soil metagenome	>S2_scaffold-98520	1,250	Bacteria
gene_372289|487_aa|-|34144|35607_S1-502773	487	Nitriliruptoria class	MBW3664163, 55.25%, 95%, Actinobacteria bacterium 0813_bin37, desert regolith metagenome	>S1_scaffold-502773	43,176	Actinomycetota phylum
gene_377903|492_aa|-|8619|10097_S1-506464	492	Nitriliruptoria class	WP_052666990, 50.00% id, 95% cov, *Nitriliruptor alkaliphilus* (Actinobacteria; Nitriliruptoria), isolate soda lake sediments	>S1_scaffold-506464	42,771	Actinomycetota phylum
gene_22128|471_aa|+|9383|10798_S1-871	471	Nitriliruptoria class	MBY5163556, 59.66%, 98%, Nitriliruptoria bacterium AS10 (Actinobacteria; Nitriliruptoria), isolate from a solar saltern	>S1_scaffold-871	38,790	Actinomycetota phylum
gene_179340|373_aa|+|2340|3461_S2-30188	373	Bacteria	MBK9712637, 40.79% id, 100% cov, Kouleothrix sp. Ribe_18-Q3-R11-54_BAT3C.183 (Chloroflexi; Chloroflexia), activated sludge metagenome	>S2_scaffold-30188	6,127	Actinomycetota phylum
P_gene_152201|234_aa|+|2921|3622_S1-8911	234	Bacteria	TMG60811, 40.84% id, 97%, Chloroflexi bacterium bin CF_6 (Terrabacteria group, Chloroflexi phylum), soil metagenome	>S1_scaffold-8911	3,622	Bacteria
gene_115949|606_aa|-|36783|38603_S1-6835	606	Bacteroidota phylum	MBT8234049, 62.15% id, 99% cov, Bacteroidia bacterium HDS-19 (Bacteroidetes; Bacteroidia), beach sand metagenome	>S1_scaffold-6835	39,973	Bacteroidota

gene_104362|637_aa|-|20748|22661_S2-5481	637	Bacteroidota phylum	HFA49006, 76.97% id, 93% cov, Bacteroidetes bacterium bin HyVt-532 (Bacteroidetes phylum), hydrothermal vent metagenome	>S2_scaffold-5481	32,725	Bacteroidota
gene_208455|606_aa|+|25789|27609_S2-58737	606	Flavobacteriia class	NNC70101, 78.78% id, 100% cov, Flavobacteriaceae bacterium SS_bin_99 (Bacteroidetes; Flavobacteriia), beach sand metagenome	>S2_scaffold-58737	31,801	Bacteroidota
gene_99361|606_aa|+|829|2649_S1-6167	606	Flavobacteriia class	NNC70101, 78.78% id, 100% cov, Flavobacteriaceae bacterium SS_bin_99 (Bacteroidetes; Flavobacteriia), beach sand metagenome	>S1_scaffold-6167	21,543	Bacteroidota
gene_290928|453_aa|+|13267|14628_S2-595999	453	Rhodothermia class (Rhodothermaeota phylum)	NNF58984, 66.14 % id, 98 % cov, Rhodothermaceae bacterium SS_bin_10 (Bacteroidetes; Rhodothermaceae), beach sand metagenome	>S2_scaffold-595999	29,212	Bacteroidota
P_gene_266192|299_aa|-|3|899_S2-167603	299	Gemmatimonadetes class (Gemmatimonadetes phylum)	MBK6423075, 45.86% id, 95% cov, Gemmatimonadetes bacterium AalE_18-Q3-R2-46_MAXAC.133 (Gemmatimonadetes phylum), activated sludge metagenome	>S2_scaffold-167603	1,500	Bacteria
gene_239810|516_aa|+|5323|6873_S1-30231	516	Bacteria	MSQ99261, 48.84% id, 99% cov, Xanthomonadales bacterium Baikal-deep-G148 (Gammaproteobacteria; Xanthomonadales), freshwater metagenome	>S1_scaffold-30231	42,159	Bacteria
P_gene_147748|408_aa|+|8240|9463_S1-8550	408	Bacteria	MSQ99261, 54.77% id, 99% cov, Xanthomonadales bacterium Baikal-deep-G148 (Gammaproteobacteria; Xanthomonadales), freshwater metagenome	>S1_scaffold-8550	9,463	Proteobacteria
P_gene_299546|329_aa|+|3|992_S1-84846	329	Bacteria	NNL65465, 40.83% id, 97% cov, Myxococcales bacterium bin Site_A26 (Deltaproteobacteria; Myxococcales), beach sand metagenome	>S1_scaffold-84846	1,535	Bacteria
gene_198874|473_aa|+|973|2394_S1-15041	473	Proteobacteria phylum	HIG72131, 39.19% id, 99% cov, Myxococcales bacterium bin UWMA-0315 (Deltaproteobacteria; Myxococcales), Hydrothermal plume metagenome	>S1_scaffold-15041	4,840	Bacteria
gene_91648|476_aa|-|1477|2907_S2-4722	476	Pseudomonadota phylum	MBV6629737, 53.14% id, 99% cov, Oceanococcus sp. MS8 (Gammaproteobacteria; Chromatiales), marine metagenome	>S2_scaffold-4722	6,224	Proteobacteria
gene_107505|505_aa|+|8811|10328_S1-6486	505	Pseudomonadota phylum	MBI3783179, 53.27% id, 95% cov, Deltaproteobacteria bacterium NC_groundwater_1022_Pr1_S-0.65um_62_10, groundwater metagenome	>S1_scaffold-6486	37,294	Pseudomonadota
gene_10315|531_aa|+|28831|30426_S1-371	531	Pseudomonadota phylum	MBY0464809, 74.70% id, 95% cov, Burkholderiales bacterium co.spades.DASTOOL.1kb_001 (Betaproteobacteria; Burkholderiales), drinking water metagenome	>S1_scaffold-371	36,618	Betaproteobacteria
gene_152785|491_aa|-|997|2472_S1-8959	491	Pseudomonadota phylum	MAE93893, 45.15% id, 96% cov, Deltaproteobacteria bacterium bin ARS66 (Proteobacteria; Deltaproteobacteria), marine metagenome	>S1_scaffold-8959	22,759	Alphaproteobacteria
gene_152786|492_aa|-|2506|3984_S1-8959	492	Gammaproteobacteria class	NCF17158, 56.40% id, 98% cov, Haliea sp. bin.47 (Gammaproteobacteria; Cellvibrionales), marine sediment metagenome			
gene_228988|471_aa|-|1860|3275_S1-25407	471	Alphaproteobacteria class	WP_066590590, 57.42% id, 100% cov, *Sphingomonas pruni* (Alphaproteobacteria; Sphingomonadales), isolate roots of *Prunus persica*	>S1_scaffold-25407	4,765	Bacteria

P_gene_228989|493_aa|-|3282|4763_S1-25407	493	Bacteria	MSO38604, 49.69% id, 96% cov, Acidimicrobiia bacterium Baikal-deep-G23 (Actinobacteria; Acidimicrobiia), freshwater metagenome			
gene_173292|492_aa|+|9506|10984_S2-25503	492	Gammaproteobacteria class	WP_195777436, 57.64% id, 98% cov, *Pseudomonas* sp. N040 (Gammaproteobacteria; Pseudomonadales), isolate	>S2_scaffold-25503	12,661	Pseudomonadota
gene_173293|493_aa|+|11025|12506_S2-25503	493	Pseudomonadota phylum	MAE93893, 44.03% id, 95% cov, Deltaproteobacteria bacterium bin ARS66 (Proteobacteria; Deltaproteobacteria), marine metagenome			
gene_99030|526_aa|-|28589|30169_S1-6153	526	Alphaproteobacteria class	NQV94142, 88.40% id, 100% cov, Sphingomonadales bacterium bin.327 (Alphaproteobacteria; Sphingomonadales), aquatic metagenome	>S1_scaffold-6153	39,220	Alphaproteobacteria
gene_77849|522_aa|-|20642|22210_S2-4128	522	Alphaproteobacteria class	WP_168820200, 79.17% id, 100% cov, *Parasphingorhabdus halotolerans* (Alphaproteobacteria; Sphingomonadales), marine sediment isolate	>S2_scaffold-4128	33,798	Alphaproteobacteria
P_gene_113451|405_aa|+|2|1219_S1-6726	405	Alphaproteobacteria class	WP_160604585, 87.65% id, 80% cov, *Pontixanthobacter aquaemixtae* (Alphaproteobacteria; Sphingomonadales), isolate aquatic environment	>S1_scaffold-6726	33,025	Alphaproteobacteria
gene_46122|482_aa|-|5381|6829_S2-3011	482	Alphaproteobacteria class	WP_009465335, 64.12% id, 98% cov, *Ahrensia* sp., marine environment isolate	>S2_scaffold-3011	33,009	Alphaproteobacteria
gene_178976|526_aa|-|1451|3031_S2-29793	526	Alphaproteobacteria class	WP_164354154, 65.77% id, 91% cov, *Sulfitobacter* sp. JBTF-M27 (Alphaproteobacteria; Rhodobacterales), tidal flat isolate	>S2_scaffold-29793	8,962	Alphaproteobacteria
gene_189772|514_aa|+|414|1958_S2-39605	514	Alphaproteobacteria class	WP_168817794, 70.18% id, 99% cov, *Sphingorhabdus* sp. JK6 (Alphaproteobacteria; Sphingomonadales), marine sediment isolate	>S2_scaffold-39605	2,552	Alphaproteobacteria
gene_66031|539_aa|-|36181|37800_S2-3704	539	Betaproteobacteria class	MBP9931277, 89.47% id, 88% cov, Rhodoferax sp. Gw_SlAct_bin_131 (Betaproteobacteria; Burkholderiales), wastewater metagenome	>S2_scaffold-3704	39,154	Betaproteobacteria
gene_205927|510_aa|+|3612|5144_S2-55933	510	Betaproteobacteria class	MBP6852791, 77.06% id, 100% cov, Rhodoferax sp. Go_SlAct_bin_313 (Betaproteobacteria; Burkholderiales), wastewater metagenome	>S2_scaffold-55933	35,518	Betaproteobacteria
gene_100398|510_aa|-|26882|28414_S1-6207	510	Betaproteobacteria class	CAG1019444, 69.64% id, 92% cov, Burkholderiaceae bacterium BURC (Betaproteobacteria; Burkholderiales),	>S1_scaffold-6207	35,146	Betaproteobacteria
gene_172841|566_aa|+|1753|3453_S2-25268	566	Betaproteobacteria class	MBK7529925, 84.30% id, 85% cov, Piscinibacter sp. Ejby_18-Q3-R6-50_BATAC.337 (Betaproteobacteria; Burkholderiales), activated sludge metagenome	>S2_scaffold-25268	8,827	Betaproteobacteria
P_gene_256400|348_aa|+|2|1045_S2-125317	348	Betaproteobacteria class	WP_164885059, 64.93% id, 99% cov *Aquincola rivuli* (Betaproteobacteria; Burkholderiales), isolate freshwater stream	>S2_scaffold-125317	1,047	Betaproteobacteria
P_gene_194001|316_aa|+|4673|5620_S1-14041	316	Betaproteobacteria class	MBP9931277, 85.32% id, 79% cov, Rhodoferax sp. Gw_SlAct_bin_131 (Betaproteobacteria; Burkholderiales) wastewater metagenome	>S1_scaffold-14041	5,620	Betaproteobacteria
gene_114652|459_aa|-|26344|27723_S1-6780	459	Gammaproteobacteria class	WP_104228648, 52.16% id, 99% cov, *Solimonas fluminis* (Gammaproteobacteria; Nevskiales), freshwater isolate	>S1_scaffold-6780	43,396	Gammaproteobacteria
gene_235529|554_aa|-|4708|6372_S2-87379	554	Gammaproteobacteria class	WP_113954689, 75.20% id, 88% cov, *Arenicella xantha* (Gammaproteobacteria; Arenicellales), marine sediment isolate	>S2_scaffold-87379	41,755	Gammaproteobacteria

gene_133207|470_aa|+|38843|40255_S1-7634	470	Gammaproteobacteria class	MBT8138215, 92.08% id, 99% cov, Gammaproteobacteria bacterium HDS-12, beach sand metagenome	>S1_scaffold-7634	41,244	Gammaproteobacteria
gene_56930|457_aa|+|18067|19440_S2-3390	457	Gammaproteobacteria class	WP_083561842, 50.00% id, 98% cov, *Oceanococcus atlanticus* (Gammaproteobacteria; Chromatiales), deep sea sediment metagenome	>S2_scaffold-3390	41,121	Gammaproteobacteria
gene_131150|483_aa|+|24431|25882_S1-7520	483	Gammaproteobacteria class	CAA0122667, 83.23% id, 100% cov, *Halioglobus japonicus* (Gammaproteobacteria; Cellvibrionales), seawater isolate	>S1_scaffold-7520	39,669	Gammaproteobacteria
gene_135191|523_aa|+|18977|20548_S2-9385	523	Gammaproteobacteria class	MBU2677936, 79.68% id, 95% cov, Gammaproteobacteria bacterium, beach sand metagenome	>S2_scaffold-9385	39,168	Gammaproteobacteria
gene_353922|510_aa|-|17207|18739_S1-427102	510	Gammaproteobacteria class	MBT8138177, 69.67% id, 95% cov, Gammaproteobacteria bacterium HDS-12, beach sand metagenome	>S1_scaffold-427102	38,523	Gammaproteobacteria
gene_102890|540_aa|-|29233|30855_S2-5375	540	Gammaproteobacteria class	WP_113954548, 79.83% id, 88% cov, *Arenicella xantha* (Gammaproteobacteria; Arenicellales), marine sediment isolate	>S2_scaffold-5375	37,997	Gammaproteobacteria
gene_58530|492_aa|+|35464|36942_S2-3445	492	Gammaproteobacteria class	MBT8139966, 93.27% id, 99% cov, Gammaproteobacteria bacterium HDS-12, beach sand metagenome	>S2_scaffold-3445	37,566	Gammaproteobacteria
gene_148498|517_aa|+|15931|17484_S2-12909	517	Gammaproteobacteria class	WP_008252074, 70.32% id, 91% cov, *Zhongshania aliphaticivorans* (Gammaproteobacteria; Cellvibrionales), marine sediment metagenome	>S2_scaffold-12909	35,894	Gammaproteobacteria
gene_150478|527_aa|-|355|1938_S1-8768	527	Gammaproteobacteria class	TVS17608, 46.26% id, 92% cov, Gammaproteobacteria bacterium PLM2.Bin7 (Gammaproteobacteria class), soda lake metagenome	>S1_scaffold-8768	35,113	Gammaproteobacteria
gene_303445|556_aa|+|9840|11510_S1-91938	556	Gammaproteobacteria class	MBU2677936, 69.79% id, 84% cov, Gammaproteobacteria bacterium, beach sand metagenome	>S1_scaffold-91938	32,805	Gammaproteobacteria
gene_179006|479_aa|+|3120|4559_S1-11543	479	Gammaproteobacteria class	WP_148067433, 49.90% id, 100% cov, *Parahaliea aestuarii* (Gammaproteobacteria; Cellvibrionales), surface seawater isolate	>S1_scaffold-11543	31,749	Gammaproteobacteria
gene_99879|482_aa|+|21798|23246_S1-6186	482	Gammaproteobacteria class	NND57794, 63.71% id, 97% cov, Xanthomonadales bacterium SS_bin_66 (Gammaproteobacteria; Xanthomonadales), beach sand metagenome	>S1_scaffold-6186	31,379	Gammaproteobacteria
gene_136242|499_aa|-|12237|13736_S1-7812	499	Gammaproteobacteria class	MBT8138177, 81.04% id, 100% cov, Gammaproteobacteria bacterium HDS-12, beach sand metagenome	>S1_scaffold-7812	30,876	Gammaproteobacteria
gene_136191|487_aa|+|25194|26657_S1-7808	487	Gammaproteobacteria class	MBT8103023, 94.05% id, 100% cov, Gammaproteobacteria bacterium IDS-33, beach sand metagenome	>S1_scaffold-7808	30,481	Gammaproteobacteria
gene_194946|480_aa|-|494|1936_S1-14240	480	Gammaproteobacteria class	WP_149612921, 44.75% id, 97% cov, *Halioglobus* sp. NY5 (Gammaproteobacteria; Cellvibrionales), isolate	>S1_scaffold-14240	24,309	Gammaproteobacteria
gene_174966|499_aa|+|8411|9910_S1-11037	499	Gammaproteobacteria class	RZA20950, 84.80% id, 100% cov, Xanthomonadaceae bacterium bin PMG_228 (Gammaproteobacteria; Xanthomonadales), phyllosphere metagenome	>S1_scaffold-11037	20,207	Gammaproteobacteria
gene_152338|467_aa|+|387|1790_S1-8921	467	Gammaproteobacteria class	MBT8138215, 86.51% id, 100% cov, Gammaproteobacteria bacterium HDS-12, beach sand metagenome	>S1_scaffold-8921	6,833	Gammaproteobacteria
gene_229156|459_aa|-|279|1658_S2-79535	459	Gammaproteobacteria class	MBK7539081, 55.92% id, 99% cov, Myxococcales bacterium Ejby_18-Q3-R6-50_BATAC.353 (Deltaproteobacteria; Myxococcales), activated sludge metagenome	>S2_scaffold-79535	1,674	Gammaproteobacteria

gene_93495|476_aa|+|18194|19624_S1-5938	476	Gammaproteobacteria class	MBJ20470, 85.23% id, 99% cov, Deltaproteobacteria bacterium (Proteobacteria; Deltaproteobacteria), marine metagenome	>S1_scaffold-5938	38,629	Bacteria
gene_186232|456_aa|+|1301|2671_S2-36063	456	Gammaproteobacteria class	TAM33335, 36.76% id, 99% cov, Sinobacteraceae bacterium FW106_bin.9 (Gammaproteobacteria; Nevskiales), groundwater metagenome	>S2_scaffold-36063	4,119	Bacteria
gene_217045|464_aa|-|1012|2406_S1-20831	464	Gammaproteobacteria class	HIG39735, 76.51% id, 100% cov, Gammaproteobacteria bacterium bin UWMA-0282 (Gammaproteobacteria class), Hydrothermal plume metagenome	>S1_scaffold-20831	4,959	Pseudomonadota
gene_108008|517_aa|+|7271|8824_S1-6508	517	Gammaproteobacteria class	MBK6738559, 56.07% id, 99% cov, Haliea sp. Aved_18-Q3-R54-62_BATAC.558 (Gammaproteobacteria; Cellvibrionales), activated sludge metagenome	>S1_scaffold-6508	30,047	Pseudomonadota
P_gene_248337|385_aa|-|1|1155_S2-106716	385	Gammaproteobacteria class	WP_189399168, 73.89% id, 99% cov, *Arenicella chitinivorans* (Gammaproteobacteria; Arenicellales), isolate sea urchin	>S2_scaffold-106716	1,629	Pseudomonadota
P_gene_297863|399_aa|+|77|1273_S1-82317	339	Gammaproteobacteria class	TXH05882, 59.11% id, 100% cov, Sinobacteraceae bacterium Bin_35_3 (Gammaproteobacteria; Nevskiales), wastewater metagenome	>S1_scaffold-82317	1,275	Gammaproteobacteria
P_gene_287484|335_aa|+|1|1008_S1-68215	335	Gammaproteobacteria class	WP_205527129, 60.26% id, 93% cov, *Solimonas* sp. K1W22B-7 (Gammaproteobacteria; Nevskiales), isolate	>S1_scaffold-68215	1,307	Gammaproteobacteria
P_gene_106318|331_aa|-|1|993_S2-5618	331	Gammaproteobacteria class	MBT8139966, 85.20% id, 100% cov, Gammaproteobacteria bacterium HDS-12 (Proteobacteria; Gammaproteobacteria), beach sand metagenome	>S2_scaffold-5618	1,199	Gammaproteobacteria
P_gene_258027|248_aa|+|482|1225_S1-40974	248	Gammaproteobacteria class	MBR9909518, 44.07% id, 95% cov, Gammaproteobacteria bacterium P03SST1bin.25 (Proteobacteria; Gammaproteobacteria), oxic surface sediment metagenome	>S1_scaffold-40974	1,226	Gammaproteobacteria
P_gene_228839|187_aa|-|3|563_S1-25384	187	Gammaproteobacteria class	WP_158523127, 52.17% id, 72% cov, *Oceanococcus atlanticus* (Gammaproteobacteria; Chromatiales), deep sea sediment isolate	>S1_scaffold-25384	6,909	Gammaproteobacteria
P_gene_264175|175_aa|-|3|527_S2-154905	175	Gammaproteobacteria class	WP_148068479, 84.48% id, 99% cov, *Parahaliea aestuarii* (Gammaproteobacteria; Cellvibrionales), surface seawater isolate	>S2_scaffold-154905	1,085	Gammaproteobacteria
gene_155764|485_aa|-|5507|6964_S2-15752	485	Deltaproteobacteria class	MBJ21504, 83.12% id, 98% cov, Deltaproteobacteria bacterium bin SP3084 (Deltaproteobacteria class), marine metagenome	>S2_scaffold-15752	28,594	Bacteria
gene_184893|477_aa|+|622|2055_S2-35065	477	Deltaproteobacteria class	KPK53749, 61.67% id, 97% cov, Myxococcales bacterium SG8_38_1 (Proteobacteria; Deltaproteobacteria), sediment metagenome	>S2_scaffold-35065	3,275	Bacteria
P_gene_251745|360_aa|-|1|1080_S2-113968	360	Deltaproteobacteria class	RLB43410, 63.69% id, 99% cov, Deltaproteobacteria bacterium bin B9_G4 (Deltaproteobacteria class), marine sediment metagenome	>S2_scaffold-113968	1,153	Pseudomonadota
P_gene_283037|301_aa|+|1|906_S1-63491	301	Deltaproteobacteria class	MBB84471, 72.69% id, 86% cov, Deltaproteobacteria bacterium bin SP124 (Deltaproteobacteria class), marine metagenome	>S1_scaffold-63491	1,389	Bacteria
P_gene_259276|144_aa|+|611|1042_S1-41935	144	Deltaproteobacteria class	MBB84471, 81.25% id, 100% cov, Deltaproteobacteria bacterium bin SP124 (Deltaproteobacteria class), marine metagenome	>S1_scaffold-41935	1,043	Bacteria

P_, partial sequence. The taxonomic binning of GH30 homolog sequences was performed using the lowest common ancestor (LCA) algorithm in Megan6 (Huson et al. 2016, PLoS Comp Biol 12:e1004957). The taxonomic assignment of scaffolds was obtained from a consensus between the assignment using PhylopythiaS (Patil et al. 2012, PloS one 7:e38581.) and the assignment of all the sequences in the scaffold using Megan6. Blastp analyses: September 21, 2021; id, identity; cov, coverage.
