# Supplementary material for: High wax ester and triacylglycerol biosynthesis potential in coastal sediments of Antarctic and Subantarctic environments
Source: PLoS One. 2023 Jul 17;18(7):e0288509. doi: 10.1371/journal.pone.0288509 (PMC10351704; doi:10.1371/journal.pone.0288509)
Supplement: S7 Table — (PDF) [file pone.0288509.s007.pdf]

S7 Table. Pfam domains identified in the deduced amino acid sequences of genes located in the analyzed genomic contexts.

**Neutral lipid synthesis**		
**Reference**	**Predicted gene function**	**PFAM**
	WS/DGAT	PF03007
	1-acyl-sn-glycerol-3-phosphate acyltransferase / hydrolase	PF01553, PF12710
	Acyl-CoA ligase	PF00501, PF13193
	1-acyl-sn-glycerol-3-phosphate acyltransferase	PF01553
**Fatty acid synthesis and degradation**		
**Reference**	**Predicted gene function**	**PFAM**
	Short-chain dehydrogenase, possible enoyl-(ACP) reductase	PF00106, PF13561
	Acyl-CoA dehydrogenase	PF00441, PF02771, PF02770, PF08028
	3-ketoacyl-CoA thiolase	PF00108, PF02803
	Enoyl-Coa hydratase	PF00378, PF16113
**Others**		
**Reference**	**Predicted gene function**	**PFAM**
	Diacylglycerol kinase	PF00781
	Thioesterase	PF02089
	Hydrolase, possible lipase	PF00561
	Transcriptional regulator	PF12840, PF00126, PF13560, PF01022, PF00440, PF17754, PF01037, PF13404, PF13412, PF00392, PF13305, PF00072
	Glycerol-3-phosphate dehydrogenase	PF07479, PF01210
	Alcohol dehydrogenase	PF00107, PF08240
	Glyceraldehyde-3-phosphate dehydrogenase	PF00044, PF02800
