# Supplementary material for: High wax ester and triacylglycerol biosynthesis potential in coastal sediments of Antarctic and Subantarctic environments
Source: PLoS One. 2023 Jul 17;18(7):e0288509. doi: 10.1371/journal.pone.0288509 (PMC10351704; doi:10.1371/journal.pone.0288509)
Supplement: S1 Fig — (A) Relative abundance of putative WS/DGAT sequences in different environmental matrices. On top of each bar, the number of analyzed metagenomes is indicated, and the bars indicate the average value and the standard deviation of the ratio between the relative abundance of WS/DGAT homolog sequences and the relative abundance of twelve single-copy genes. (B) Relative abundance of putative WS/DGAT sequences in deep ocean samples (2000–4000 m depth) from the Global Malaspina Expedition (Salazar et al. 2016), and water temperature. The metagenome IDs (IMG/M system, https://img.jgi.doe.gov/) used in the analysis are indicated in S4 Table. (PDF) [file pone.0288509.s008.pdf]

A

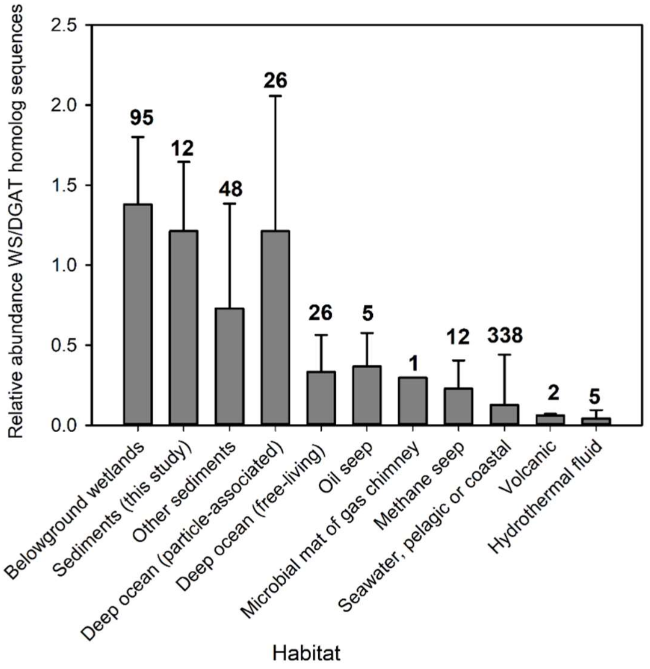

B

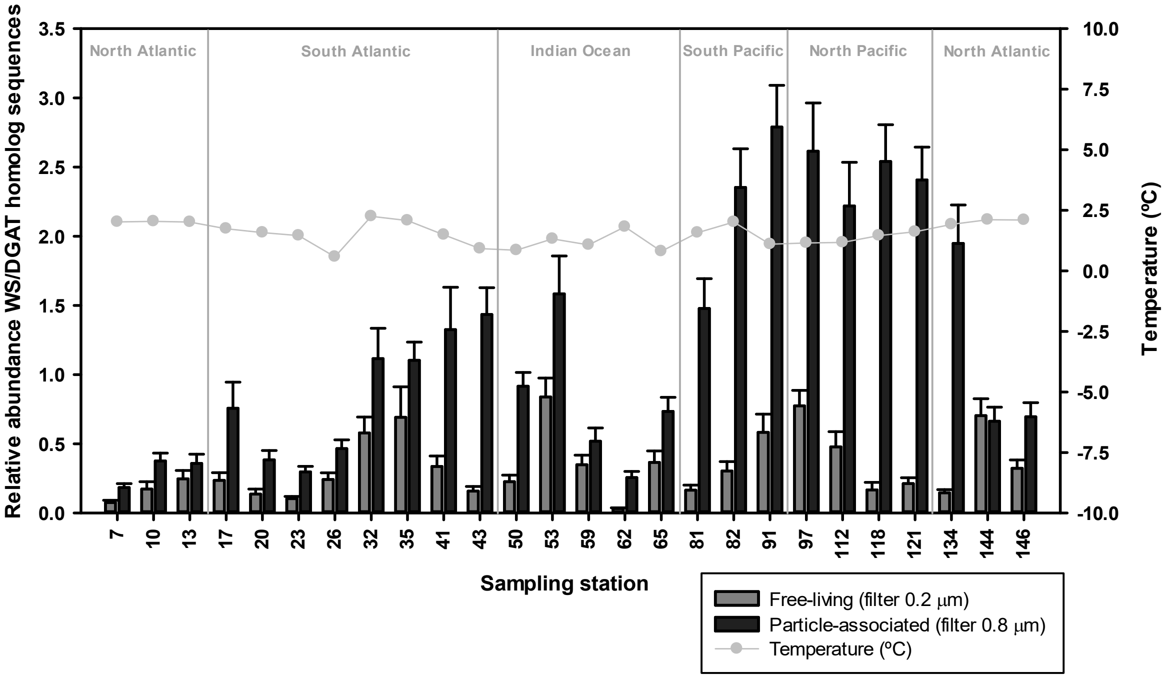

S1 Fig. Relative abundance of putative WS/DGAT sequences in marine metagenomes. (A) Relative abundance of putative WS/DGAT sequences in different environmental matrices. On top of each bar, the number of analyzed metagenomes is indicated, and the bars indicate the average value and the standard deviation of the ratio between the relative abundance of WS/DGAT homolog sequences and the relative abundance of twelve single-copy genes. **(B)** Relative abundance of putative WS/DGAT sequences in deep ocean samples (2000 - 4000 m depth) from the Global Malaspina Expedition (Salazar et al. 2016), and water temperature. The metagenome IDs (IMG/M system, https://img.jgi.doe.gov/) used in the analysis are indicated in Table S4.
