# Supplementary material for: High wax ester and triacylglycerol biosynthesis potential in coastal sediments of Antarctic and Subantarctic environments
Source: PLoS One. 2023 Jul 17;18(7):e0288509. doi: 10.1371/journal.pone.0288509 (PMC10351704; doi:10.1371/journal.pone.0288509)
Supplement: S2 Fig — (A) Gammaproteobacteria class. (B) Actinobacteria phylum. The sequences were analyzed using blastp (nr, 100 hits), and Megan6 LCA algorithm was used to classify each sequence at the class level. In the case of sequences from the assembled metagenome of ARG01-ARG06, values were corrected based on gene copy number, as indicated in the IMG/M system for sequences of the assembled metagenomes. (PDF) [file pone.0288509.s009.pdf]

A

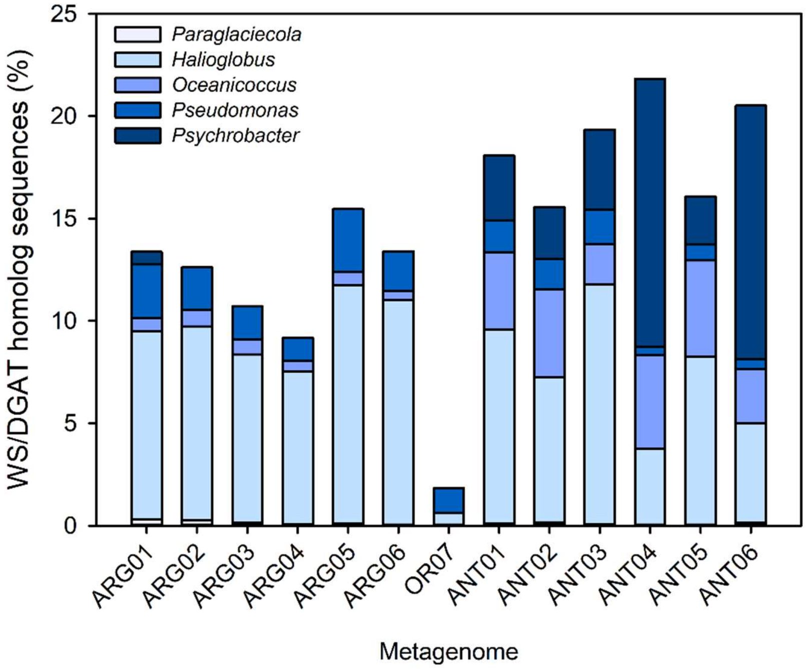

B

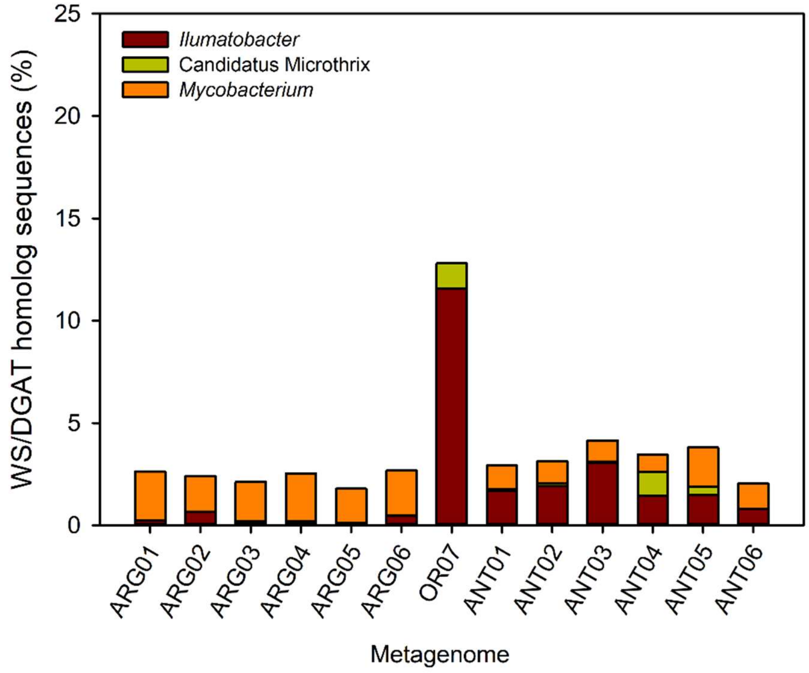

S2 Fig. Relative abundance of the most abundant taxonomic assignment at the genus level of WS/DGAT homolog sequences (estimated sequences). (A) Gammaproteobacteria class. **(B)** Actinomycetota phylum. The sequences were analyzed using blastp (nr, 100 hits), and Megan6 LCA algorithm was used to classify each sequence at the class level. In the case of sequences from the assembled metagenome of ARG01-ARG06, values were corrected based on gene copy number, as indicated in the IMG/M system for sequences of the assembled metagenomes.
