# Supplementary material for: High wax ester and triacylglycerol biosynthesis potential in coastal sediments of Antarctic and Subantarctic environments
Source: PLoS One. 2023 Jul 17;18(7):e0288509. doi: 10.1371/journal.pone.0288509 (PMC10351704; doi:10.1371/journal.pone.0288509)
Supplement: S3 Fig — The dataset contained 1,022 OPUs containing ≥ 10 sequences. Scale is arbitrary and goes from light (low relative abundance) to dark (high relative abundance). On the right, phylum or class of the representative sequence in the cluster is indicated. The symbol # next to the OPU number indicates clusters showing significantly differences between Antarctic and Subantarctic sediment metagenomes (Wilcoxon Rank Sum test corrected for multiple testing, as implemented in the R-script ANCOM). (PDF) [file pone.0288509.s010.pdf]

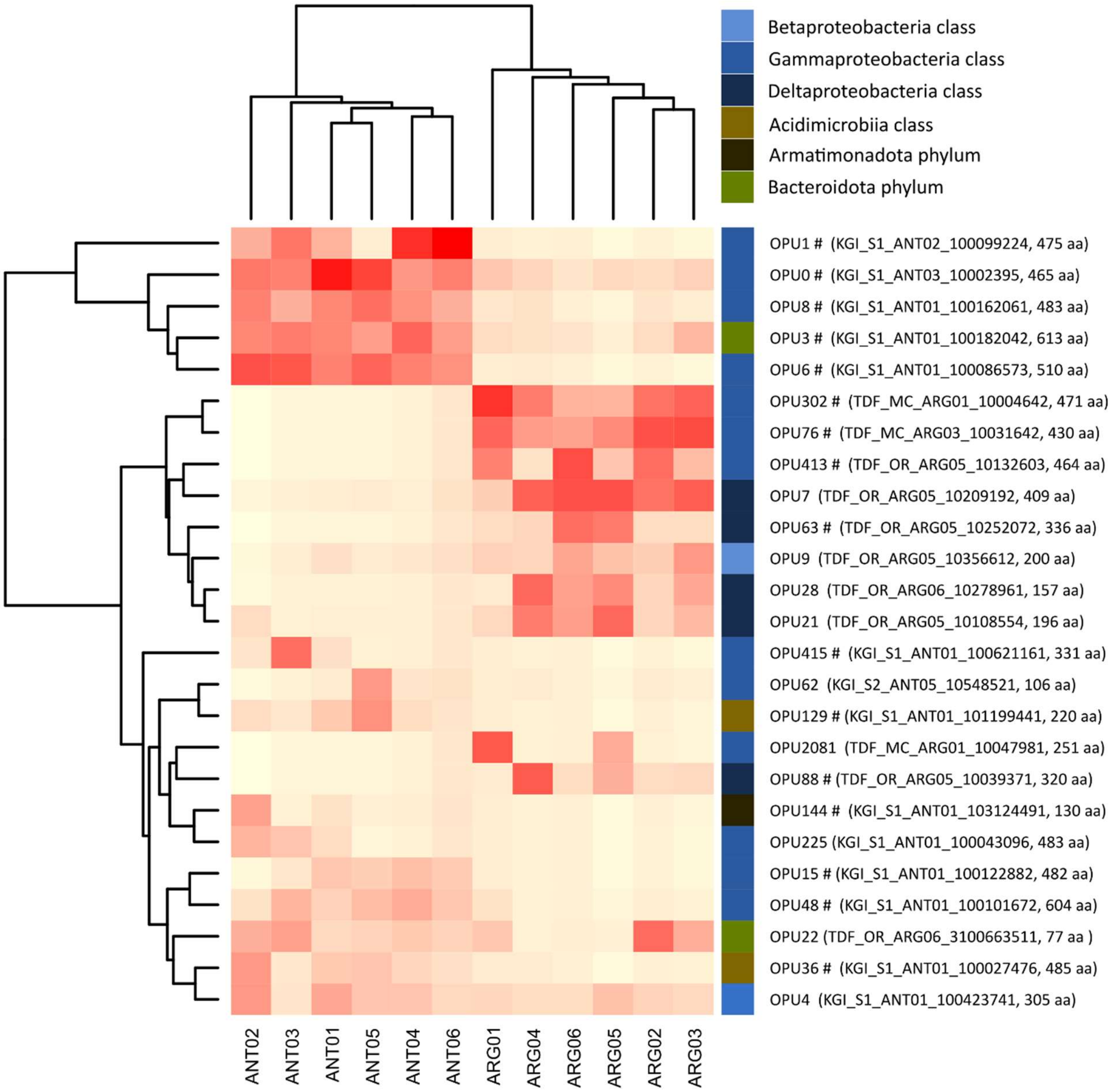

S3 Fig. Heatmap showing the 25 most abundant OPUs in the dataset (estimated values) including metagenomes from Subantarctic and Antarctic sediments. The dataset contained 1,022 OPUs containing ≥ 10 sequences. Scale is arbitrary and goes from light (low relative abundance) to dark (high relative abundance). On the right, phylum or class of the representative sequence in the cluster and its length is indicated. The symbol # next to the OPU number indicates clusters showing significantly differences between Antarctic and Subantarctic sediment metagenomes (Wilcoxon Rank Sum test corrected for multiple testing, as implemented in the R-script ANCOM).
