# Supplementary material for: High wax ester and triacylglycerol biosynthesis potential in coastal sediments of Antarctic and Subantarctic environments
Source: PLoS One. 2023 Jul 17;18(7):e0288509. doi: 10.1371/journal.pone.0288509 (PMC10351704; doi:10.1371/journal.pone.0288509)
Supplement: S4 Fig — ANT01-06, gray; ARG01-06, black. Only clusters containing more than 10 estimated sequences and including sequences identified in intertidal sediments metagenome (OR07, right) are shown. Asterisk indicates > 1 sequence from OR07 in the cluster. On the right of the boxplot, colors indicate phylum or class, which are also indicated next to the sequence name: Alph cl, Alphaproteobacteria class; Bet cl, Betaproteobacteria class; Gam cl, Gammaproteobacteria class; Del cl, Deltaproteobacteria class; Act ph, Actinomycetota phylum; Act cl, Actinomycetes class; Aci cl, Acidimicrobiia class; Bac ph, Bacteroidota phylum. (PDF) [file pone.0288509.s011.pdf]

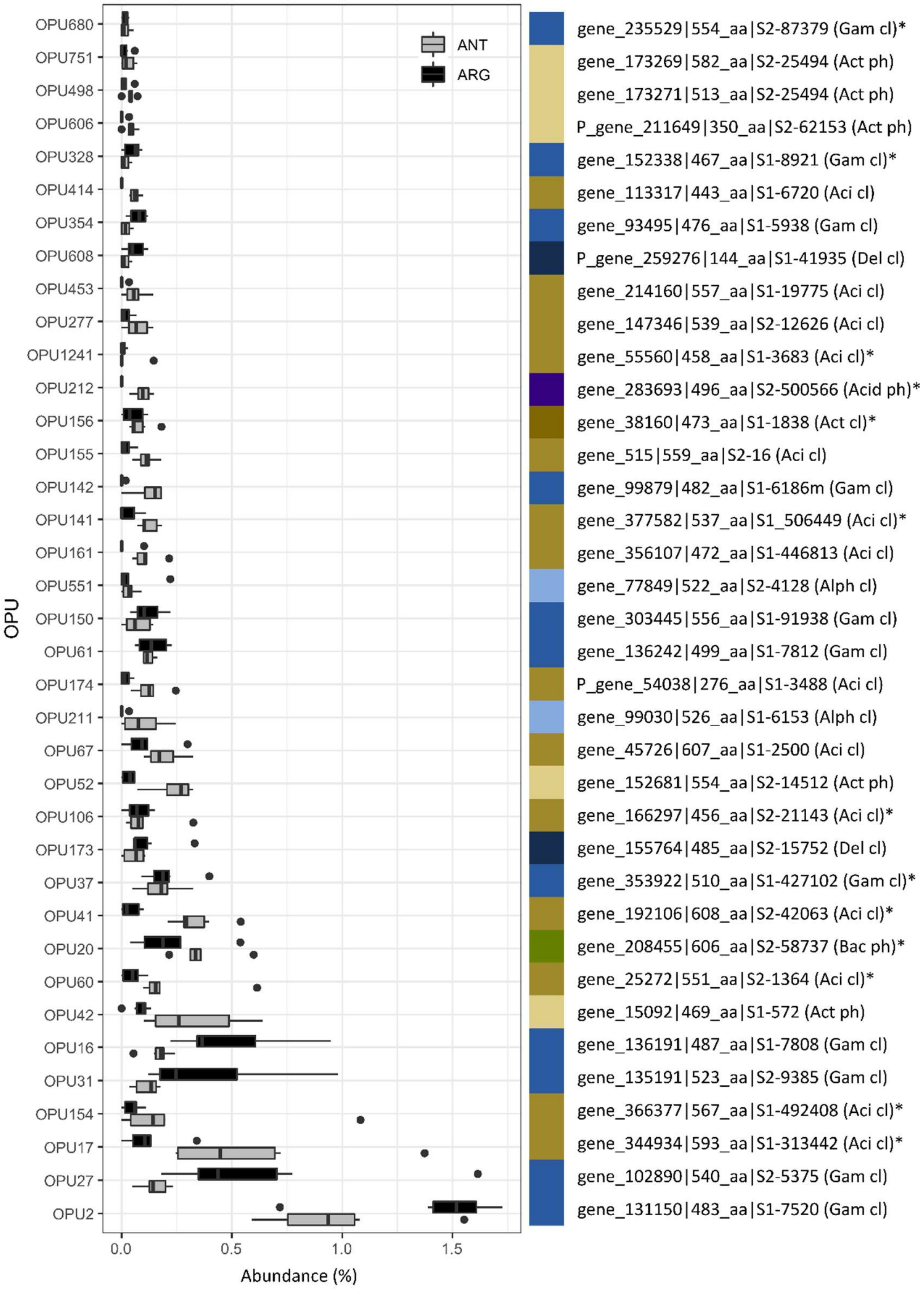

S4 Fig. Boxplot showing the relative abundance of clusters containing sequences identified in metagenomes of subtidal sediments. ANT01-06, gray; ARG01-06, black. Only clusters containing more than 10 estimated sequences and including sequences identified in intertidal sediments metagenome (OR07, right) are shown. Asterisk indicates > 1 sequence from OR07 metagenome in the cluster. On the right of the boxplot, colors indicate phylum or class, which are also indicated next to the sequence name: Alph cl, Alphaproteobacteria class; Bet cl, Betaproteobacteria class; Gam cl, Gammaproteobacteria class; Del cl, Deltaproteobacteria class; Act ph, Actinomycetota phylum; Act cl, Actinomycetes class; Aci cl, Acidimicrobiia class; Bac ph, Bacteroidota phylum.
