# Supplementary material for: High wax ester and triacylglycerol biosynthesis potential in coastal sediments of Antarctic and Subantarctic environments
Source: PLoS One. 2023 Jul 17;18(7):e0288509. doi: 10.1371/journal.pone.0288509 (PMC10351704; doi:10.1371/journal.pone.0288509)
Supplement: S5 Fig — Maximum likelihood tree of WS/DGAT homolog sequences assigned to the Actinomycetota phylum identified in the metagenomic dataset of intertidal sediments of Ushuaia Bay (OR07). Names of metagenomic sequences are shown in red, and related sequences from the IMG/M and NCBI databases, in black. The tree was built in MEGA X (Kumar et al. 2018). Bootstrap values are percent of 100 replications. (PDF) [file pone.0288509.s012.pdf]

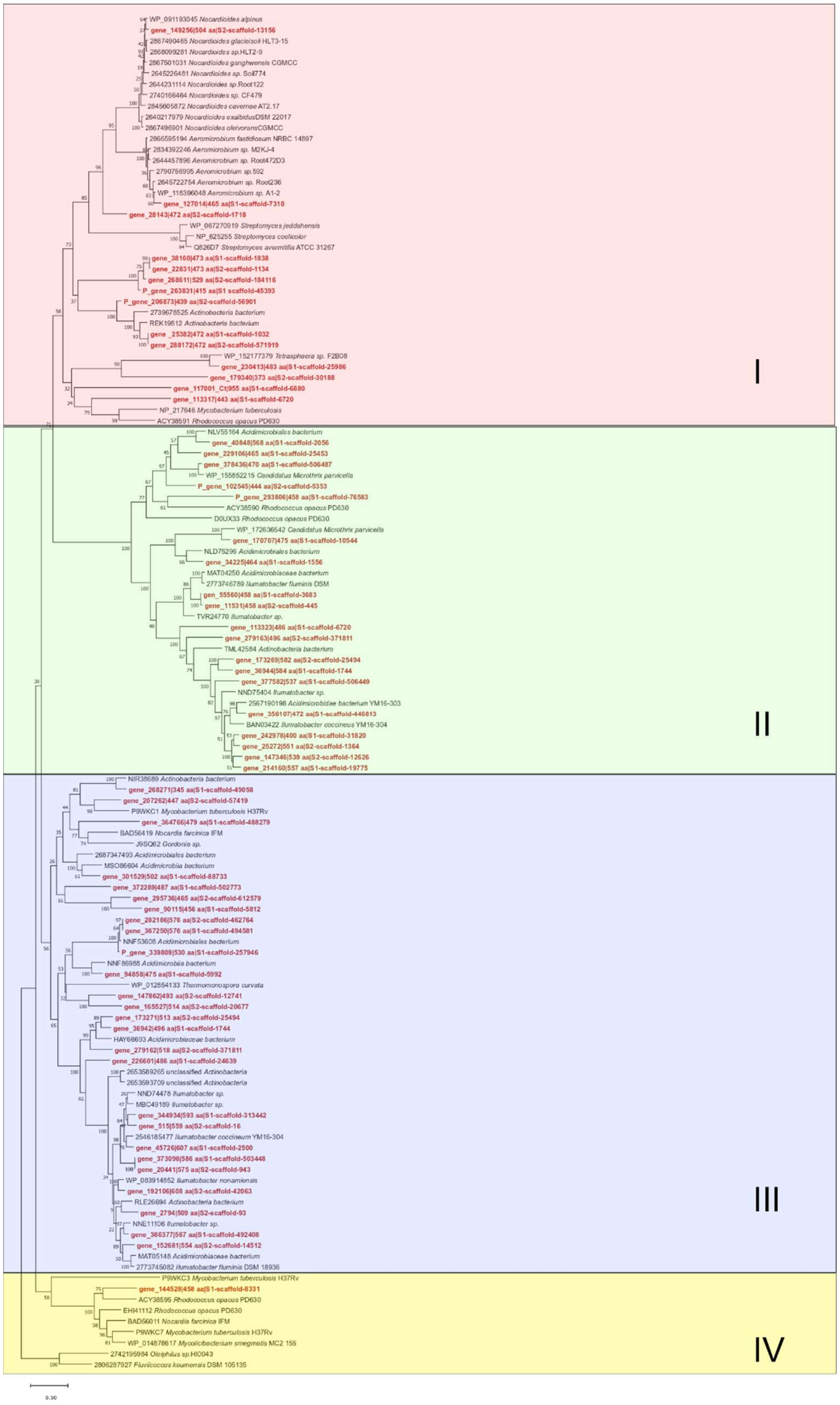

S5 Fig. Phylogenetic analysis of sequences assigned to the Actinomycetota phylum. Maximum likelihood tree of WS/DGAT homolog sequences assigned to the Actinomycetota phylum identified in the metagenomic dataset of intertidal sediments of Ushuaia Bay (OR07). Names of metagenomic sequences are shown in red, and related sequences from the IMG/M and NCBI databases, in black. The tree was built in MEGA X (Kumar et al. 2018, Nat Biotechnol 39:499-509). Bootstrap values are based on 100 replicates.
