# Supplementary material for: High wax ester and triacylglycerol biosynthesis potential in coastal sediments of Antarctic and Subantarctic environments
Source: PLoS One. 2023 Jul 17;18(7):e0288509. doi: 10.1371/journal.pone.0288509 (PMC10351704; doi:10.1371/journal.pone.0288509)
Supplement: S6 Fig — (A) Maximum-Likelihood tree of WS/DGAT homolog sequences assigned to the Betaproteobacteria class, identified in the metagenomic dataset of intertidal sediments (OR07, in red) and related sequences from public databases (in black). GEN, sequence identified in a genome; MAG, sequence identified in a metagenome-assembled genome. Bootstrap values higher than 50% based on 100 replicates are shown. The box indicates the sequences for which the genomic context and shared synteny is shown below. (B) Genomic context and shared synteny of the cluster indicated above. Gene clusters, including WS/DGAT homolog sequences and other putative genes related to fatty acid metabolism pathway are shown. Gray gradient represents % identity at the nucleotide level between scaffolds. (PDF) [file pone.0288509.s013.pdf]

A

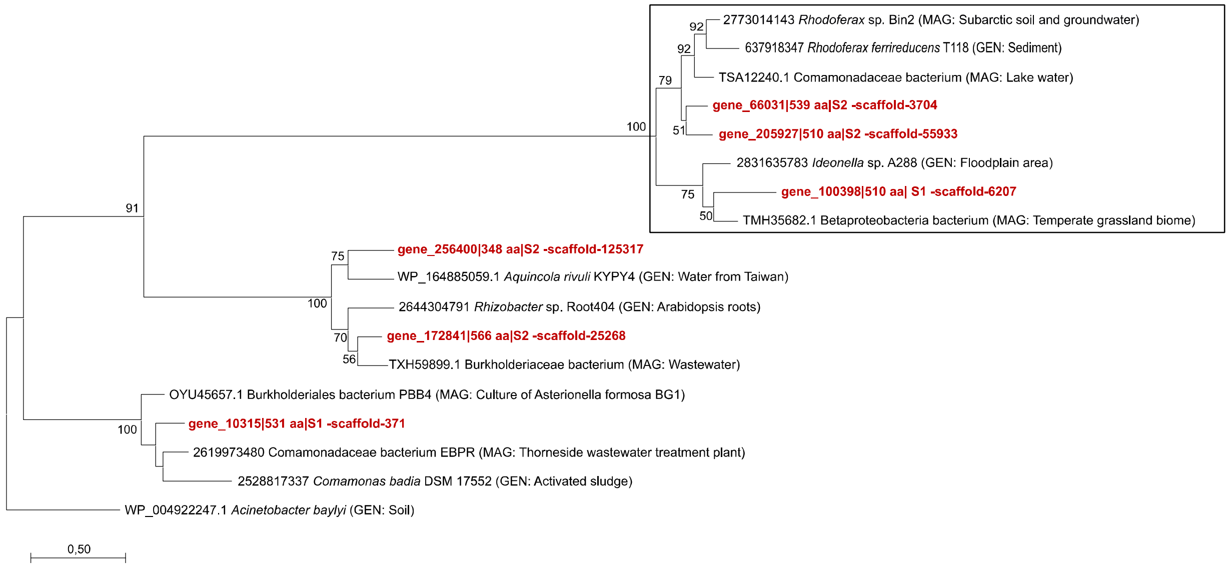

B

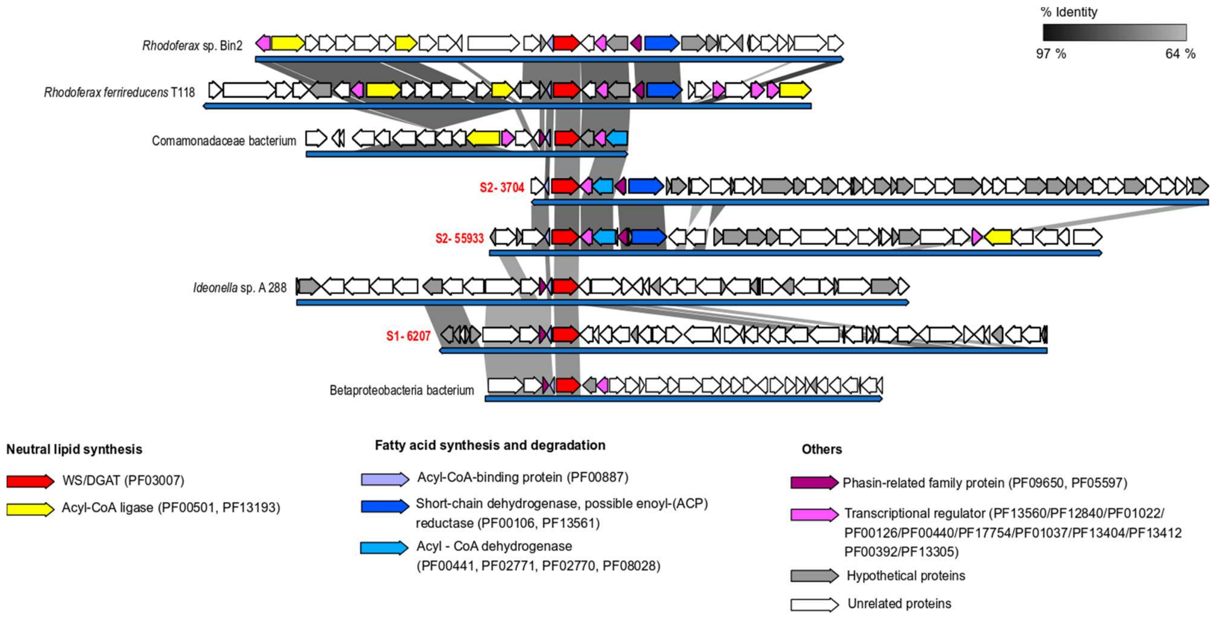

S6 Fig. Phylogenetic analysis and genomic context of sequences assigned to the Betaproteobacteria class. (A) Maximum-Likelihood tree of WS/DGAT homolog sequences assigned to the Betaproteobacteria class, identified in the metagenomic dataset of intertidal sediments (OR07, in red) and related sequences from public databases (in black). GEN, sequence identified in a genome; MAG, sequence identified in a metagenome-assembled genome. Bootstrap values (≥ 50%) are based on 100 replicates. The box indicates the sequences for which the genomic context and shared synteny is shown below. **(B)** Genomic context and shared synteny of the cluster indicated above. Gene clusters, including WS/DGAT homolog sequences and other putative genes related to fatty acid metabolism pathway are shown. Gray gradient represents % identity at the nucleotide level between scaffolds.
