# Supplementary material for: High wax ester and triacylglycerol biosynthesis potential in coastal sediments of Antarctic and Subantarctic environments
Source: PLoS One. 2023 Jul 17;18(7):e0288509. doi: 10.1371/journal.pone.0288509 (PMC10351704; doi:10.1371/journal.pone.0288509)
Supplement: S7 Fig — (A) Maximum-Likelihood tree of WS/DGAT homolog sequences assigned to Gammaproteobacteria class, identified in the metagenomic dataset of intertidal sediments (OR07, in red) and related sequences from public databases (in black). GEN, sequence identified in a genome; MAG, sequence identified in a metagenome-assembled genome. Bootstrap values higher than 50% based on 100 replicates are shown. The box indicates the sequences for which the shared synteny is shown below. (B) Genomic context and shared synteny of the cluster indicated above. Gene clusters, including WS/DGAT homolog sequences and other putative enzymes of the Kennedy pathway are shown. (C) Representative gene clusters including WS/DGAT homolog sequences and other putative genes related to fatty acid metabolism pathway, and their shared synteny. Gray gradient represents percent identity at the nucleotide level between scaffolds. (PDF) [file pone.0288509.s014.pdf]

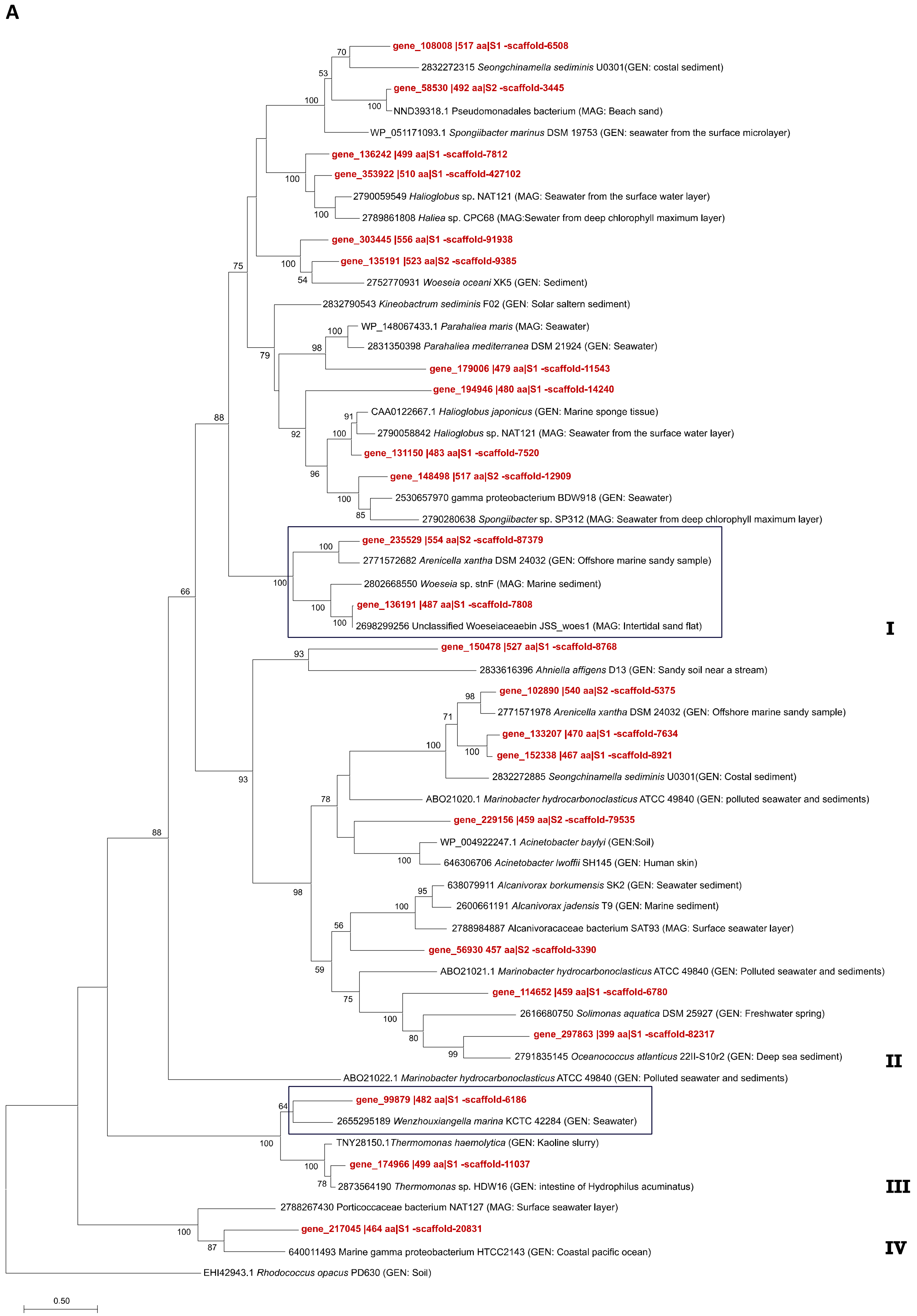
A
gene_108008 |517 aa|S1 -scaffold-6508
2832272315 Seongchinamella sediminis U0301(GEN: costal sediment)
gene_58530 |492 aa|S2 -scaffold-3445
NND39318.1 Pseudomonadales bacterium (MAG: Beach sand)
WP_051171093.1 Spongiibacter marinus DSM 19753 (GEN: seawater from the surface microlayer)
gene_136242 |499 aa|S1 -scaffold-7812
gene_353922 |510 aa|S1 -scaffold-427102
2790059549 Halioglobus sp. NAT121 (MAG: Seawater from the surface water layer)
2789861808 Haliea sp. CPC68 (MAG:Sewater from deep chlorophyll maximum layer)
gene_303445 |556 aa|S1 -scaffold-91938
gene_135191 |523 aa|S2 -scaffold-9385
2752770931 Woeseia oceani XK5 (GEN: Sediment)
2832790543 Kineobactrum sediminis F02 (GEN: Solar saltern sediment)
WP_148067433.1 Parahaliea maris (MAG: Seawater)
2831350398 Parahaliea mediterranea DSM 21924 (GEN: Seawater)
gene_179006 |479 aa|S1 -scaffold-11543
gene_194946 |480 aa|S1 -scaffold-14240
CAA0122667.1 Halioglobus japonicus (GEN: Marine sponge tissue)
2790058842 Halioglobus sp. NAT121 (MAG: Seawater from the surface water layer)
gene_131150 |483 aa|S1 -scaffold-7520
gene_148498 |517 aa|S2 -scaffold-12909
2530657970 gamma proteobacterium BDW918 (GEN: Seawater)
2790280638 Spongiibacter sp. SP312 (MAG: Seawater from deep chlorophyll maximum layer)
gene_235529 |554 aa|S2 -scaffold-87379
2771572682 Arenicella xantha DSM 24032 (GEN: Offshore marine sandy sample)
2802668550 Woeseia sp. stnF (MAG: Marine sediment)
gene_136191 |487 aa|S1 -scaffold-7808
2698299256 Unclassified Woeseiaceaebin JSS_woes1 (MAG: Intertidal sand flat)
I
gene_150478 |527 aa|S1 -scaffold-8768
2833616396 Ahniella affigens D13 (GEN: Sandy soil near a stream)
gene_102890 |540 aa|S2 -scaffold-5375
2771571978 Arenicella xantha DSM 24032 (GEN: Offshore marine sandy sample)
gene_133207 |470 aa|S1 -scaffold-7634
gene_152338 |467 aa|S1 -scaffold-8921
2832272885 Seongchinamella sediminis U0301(GEN: Costal sediment)
ABO21020.1 Marinobacter hydrocarbonoclasticus ATCC 49840 (GEN: polluted seawater and sediments)
gene_229156 |459 aa|S2 -scaffold-79535
WP_004922247.1 Acinetobacter baylyi (GEN:Soil)
646306706 Acinetobacter lwoffii SH145 (GEN: Human skin)
638079911 Alcanivorax borkumensis SK2 (GEN: Seawater sediment)
2600661191 Alcanivorax jadensis T9 (GEN: Marine sediment)
2788984887 Alcanivoracaceae bacterium SAT93 (MAG: Surface seawater layer)
gene_56930 457 aa|S2 -scaffold-3390
ABO21021.1 Marinobacter hydrocarbonoclasticus ATCC 49840 (GEN: Polluted seawater and sediments)
gene_114652 |459 aa|S1 -scaffold-6780
2616680750 Solimonas aquatica DSM 25927 (GEN: Freshwater spring)
gene_297863 |399 aa|S1 -scaffold-82317
2791835145 Oceanococcus atlanticus 22II-S10r2 (GEN: Deep sea sediment)
II
ABO21022.1 Marinobacter hydrocarbonoclasticus ATCC 49840 (GEN: Polluted seawater and sediments)
gene_99879 |482 aa|S1 -scaffold-6186
2655295189 Wenzhouxiangella marina KCTC 42284 (GEN: Seawater)
TNY28150.1Thermomonas haemolytica (GEN: Kaoline slurry)
gene_174966 |499 aa|S1 -scaffold-11037
2873564190 Thermomonas sp. HDW16 (GEN: intestine of Hydrophilus acuminatus)
III
2788267430 Porticoccaceae bacterium NAT127 (MAG: Surface seawater layer)
gene_217045 |464 aa|S1 -scaffold-20831
640011493 Marine gamma proteobacterium HTCC2143 (GEN: Coastal pacific ocean)
IV
EHI42943.1 Rhodococcus opacus PD630 (GEN: Soil)
0.50

B

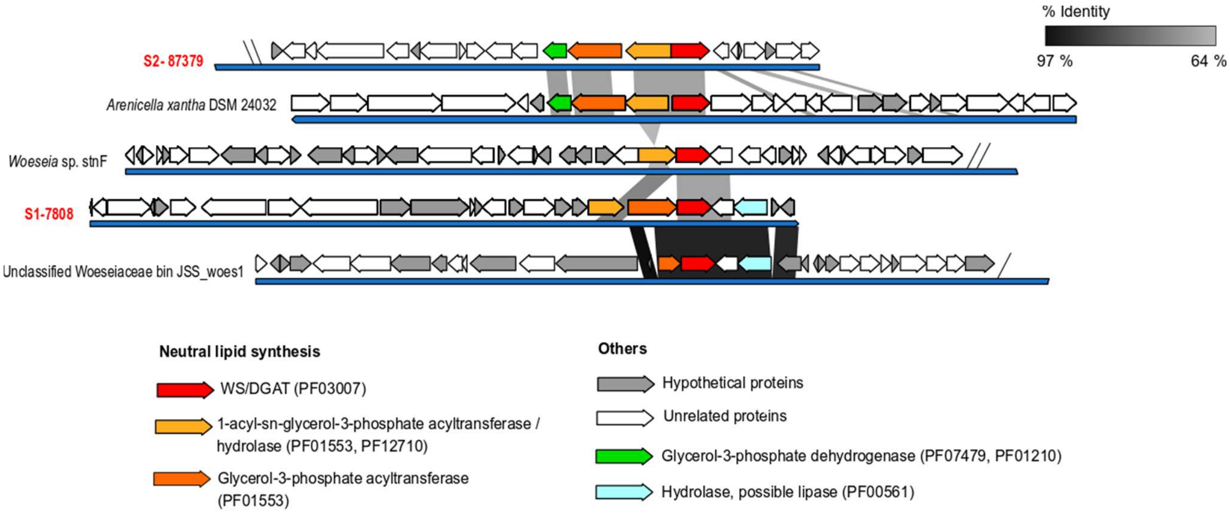

C

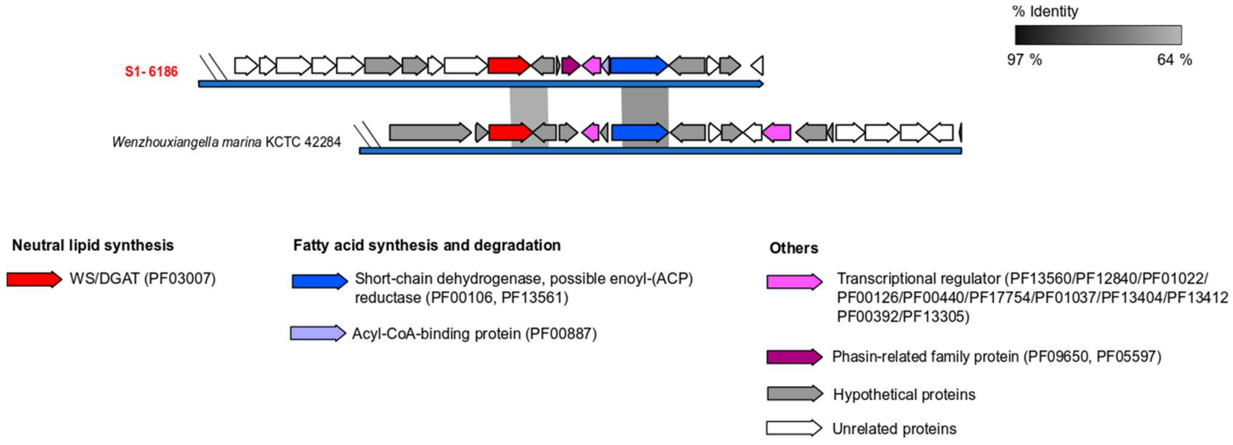

S7 Fig. Phylogenetic analysis and genomic context of sequences assigned to the Gammaproteobacteria class. (A) Maximum-Likelihood tree of WS/DGAT homolog sequences assigned to Gammaproteobacteria class, identified in the metagenomic dataset of intertidal sediments (OR07, in red) and related sequences from public databases (in black). GEN, sequence identified in a genome; MAG, sequence identified in a metagenome-assembled genome. Bootstrap values (> 50%) are based on 100 replicates. The box indicates the sequences for which the shared synteny is shown below. **(B)** Genomic context and shared synteny of the cluster indicated above. Gene clusters, including WS/DGAT homolog sequences and other putative enzymes of the Kennedy pathway are shown. **(C)** Representative gene clusters including WS/DGAT homolog sequences and other putative genes related to fatty acid metabolism pathway, and their shared synteny. Gray gradient represents percent identity at the nucleotide level between scaffolds.
