# Supplementary material for: High wax ester and triacylglycerol biosynthesis potential in coastal sediments of Antarctic and Subantarctic environments
Source: PLoS One. 2023 Jul 17;18(7):e0288509. doi: 10.1371/journal.pone.0288509 (PMC10351704; doi:10.1371/journal.pone.0288509)
Supplement: S8 Fig — Maximum-Likelihood tree of WS/DGAT homolog sequences assigned to Deltaproteobacteria class, identified in the metagenomic dataset of intertidal sediments (OR07, in red) and related sequences from public databases (in black). GEN, sequence identified in a genome; MAG, sequence identified in a metagenome assembled genome. Bootstrap values higher than 50% based on 100 replicates are shown. (PDF) [file pone.0288509.s015.pdf]

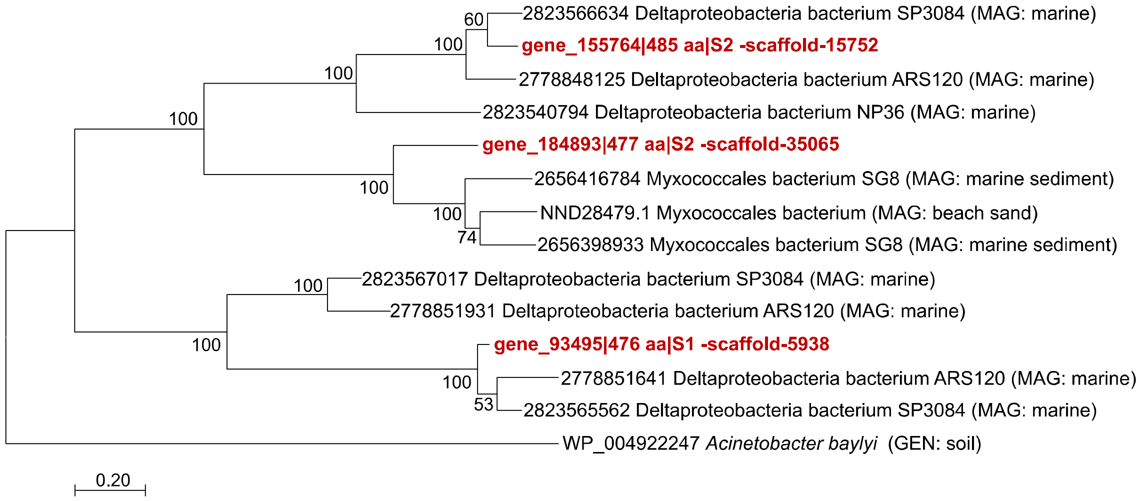

S8 Fig. Phylogenetic analysis of sequences assigned to the Deltaproteobacteria class. Maximum-Likelihood tree of WS/DGAT homolog sequences assigned to Deltaproteobacteria class, identified in the metagenomic dataset of intertidal sediments (OR07, in red) and related sequences from public databases (in black). GEN, sequence identified in a genome; MAG, sequence identified in a metagenome assembled genome. Bootstrap values (> 50%) are based on 100 replicates.
