# Supplementary material for: High wax ester and triacylglycerol biosynthesis potential in coastal sediments of Antarctic and Subantarctic environments
Source: PLoS One. 2023 Jul 17;18(7):e0288509. doi: 10.1371/journal.pone.0288509 (PMC10351704; doi:10.1371/journal.pone.0288509)
Supplement: S9 Fig — (A) Maximum-Likelihood phylogenetic tree of WS/DGAT homolog sequences assigned to Bacteroidota identified in the metagenomic dataset of intertidal sediments (OR07, in red) and sequences from public databases from genomes of members of the Bacteroidota phylum containing a putative SCP-2 domain at the C-term (in black; ID with numbers, IMG/M; numbers and letters, NCBI). Only unique sequences are included in the tree. IS: isolate; MAG: metagenome assembled genome; Red: sequences identified in this study. Bootstrap values higher than 50% based on 100 replicates are shown. (B) Genomic context and shared synteny of scaffolds containing the WS/DGAT homologs included in the rectangle in the phylogenetic tree. Gray/black regions correspond to the percentage identity at the nucleotide level shown on the right. (PDF) [file pone.0288509.s016.pdf]

A

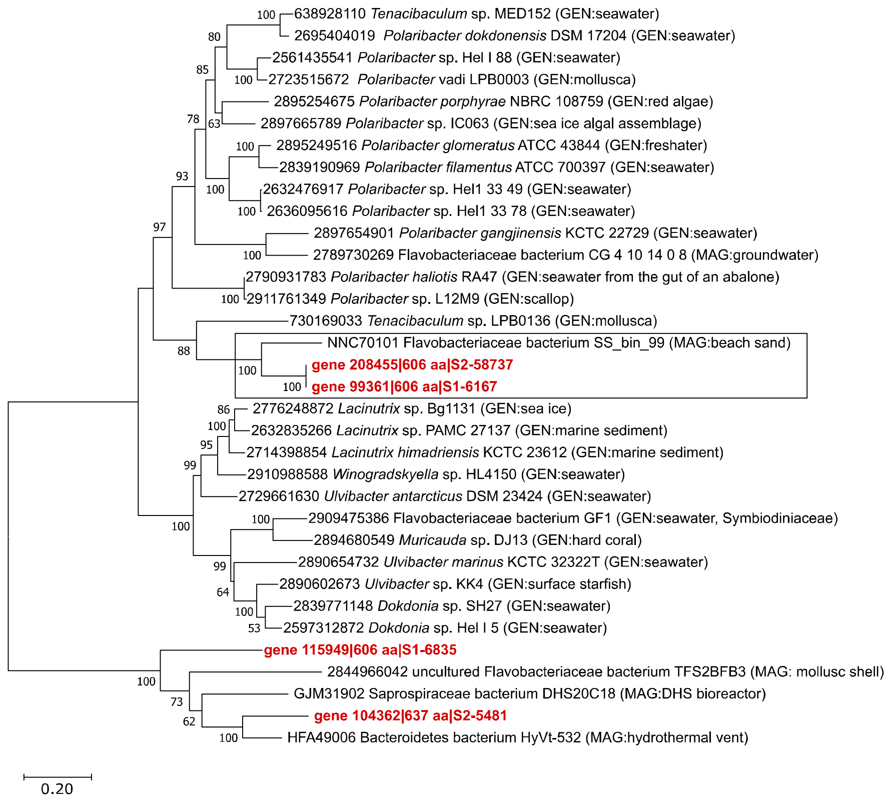

B

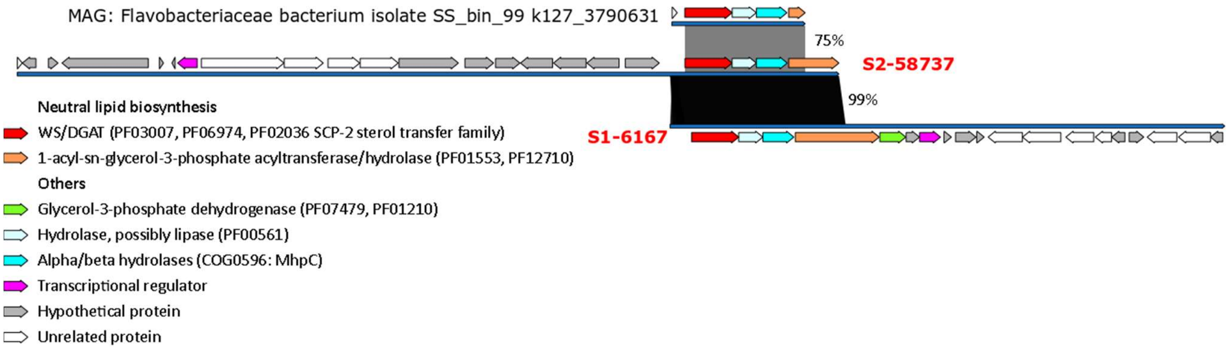

S9 Fig. Phylogenetic analysis and genomic context of sequences assigned to the Bacteroidota phylum. (A) Maximum-Likelihood phylogenetic tree of WS/DGAT homolog sequences assigned to Bacteroidetes identified in the metagenomic dataset of intertidal sediments (OR07, in red) and sequences from public databases from Bacteroidetes genomes containing a putative SCP-2 domain at the C-term (in black; ID with numbers, IMG/M; numbers and letters, NCBI). Only unique sequences are included in the tree. IS: isolate; MAG: metagenome assembled genome; Red: sequences identified in this study. Bootstrap values (> 50%) are based on 100 replicates. **(B)** Genomic context and shared synteny of scaffolds containing the WS/DGAT homologs included in the rectangle in the phylogenetic tree. Gray/black regions correspond to the percentage identity at the nucleotide level shown on the right.
